# Supplementary material for: SEMA3C drives cancer growth by transactivating multiple receptor tyrosine kinases via Plexin B1
Source: EMBO Mol Med. 2018 Jan 18;10(2):219–38. doi: 10.15252/emmm.201707689 (PMC5801490; doi:10.15252/emmm.201707689)
Supplement: Supplementary file 1 — Appendix [file EMMM-10-219-s001.pdf]

Table of Contents

Table of Contents 1
Appendix Supplemental Materials and Methods 2
Appendix Figure. S1 7
Appendix Figure. S2 9
Appendix Figure. S3 11
Appendix Figure. S4 12
Appendix Figure. S5 13
Appendix Figure. S6 15
Appendix Table S1 17
Appendix Table S2 18

Appendix Supplemental Materials and Methods:

Cell Lines

LNCaP (< passage 50), DU145 and CWR22Rv1, cell lines were purchased from American Type Culture Collection (ATCC, Manassas, VA, USA). BPH-1, courtesy Dr S. Howard (Vanderbilt University), ENZ-resistant cells (MR49F) derived from LNCaP cells were established and maintained in ENZ (10 μM) as described previously (Kuruma et al, 2013). C4-2 cells were kindly provided by Dr. Leland W.K. Chung (1992, MD Anderson Cancer Center, Houston, TX), tested and authenticated by whole-genome and whole-transcriptome sequencing on Illumina Genome Analyzer IIx platform in 2009. All other cell-lines used in this study with the exception of BPH-1 were authenticated by IDEXX Bioresearch-case 20201/21100-2014, (Columbia, MO). HEK293T, Caki-1, Caki-2, ACHN, T24, and U-87MG were purchased from ATCC (Manassas, VA), cells were obtained from the European Collection of Cell cultures and CHO-S cells were purchased from Invitrogen (Burlington, ON). Cell-lines were maintained as recommended. $LNCaP_{empty}$ and $LNCaP_{SEMA3C\text{-}FL}$ cells were derived by transduction with LB-U (Empty) or SEMA3C-FL lentivirus. Stable cell-lines were maintained in media supplemented with blasticidin (5μg/ml) from Invitrogen (Burlington, ON).

Antibodies, chemicals and reagents

Antibodies to the following were used at 1:1000 dilution: pEGFR (Y1068 or Y1148), pHER2/ErB2 (Y877 or Y1248), HER2/ErbB2 (29D8), pSRC (Y416), SRC (L4A1), pSHC (Y239/240), p42/44 pMAPK (T202/Y204) (1:200 for IHC), p42/44 MAPK (L34F12, 1:2000), pMET (Y1234/1235), MET (25H2), pGab1 (Y627, C32H2),Gab1(3232) cleaved PARP (Asp214), (9541), PARP (9542),cleaved caspase-3 (5A1E, 9664), (Cell Signaling, Inc., Beverly MA), pAKT (Ser473), pan-AKT(1:2000), (BioSource International, Camarillo,CA), EGFR(C-2) 1:500, SEMA3C (N-20) 1:2000, 1:200 for IHC, Plexin B1 (H300),1:1000, Plexin D1 (H-70), 1:1000, SHC,1:2000, Androgen receptor (N20) (Sc-816), 1:1000, PSA(A67-B3, Sc7316),1:1000, 1:50 for IHC (Santa Cruz Biotechnology Santa Cruz,CA), CD31, 1:100 for IHC (BD transduction Laboratories, Lexington, KY), Actin (A2066),1:2000, Vinculin,1:5000, anti-Human IgG(Fc specific),1:5,000, clone GG-7, (Sigma-Aldrich, St Louis, MO), Ki67,(1:500) and TUNEL, (Roche diagnostics, Laval, PQ), GAPDH (Novus Biologicals, Littleton, CO). Peroxidase conjugated Goat anti-rabbit, (1:3000) (DAKO, Mississauga,On), LICOR secondary antibodies, (1:10,000), anti-Rabbit IR680 and IR800, anti-mouse, IR680 and IR800 (Rockland, Gilbertsville, PA) and anti-Sheep IR800, anti-Goat IR680 (Pierce Biotechnology, Rockford,IL). Antibodies were used according to manufacturer's instructions. The following chemicals and reagents were used: Human recombinant EGF (GF316), Furin Inhibitor1 (344930), (Millipore, Darmstadt, Germany), Human recombinant IgG_1Fc, Human NRP-2 (AF2215, 1:1000), Human Plexin B1 (MAB3749, 1:1000), Human Plexin D1 (MAB41601, 1:1000), (R&D systems, Inc., Minneapolis, MN), Human NRP-1 antibody (1:1000) (Origene Technologies, Inc., Rockville, MD), PlexinB1 (c-terminal region, PP1841 (1:1000), ECM Biosciences, Versialles, KY), enzalutamide (Haoyuan Chemexpress Co. Ltd., China) R1881 (NLP-005; Perkin-Elmer, Waltham, MA), Docetaxel (Sanofi-Aventis, Bridgewater,NJ), EGF inhibitor, Erlotinib Hydrochloride, (Santa Cruz Biotechnology Santa Cruz,CA), Met inhibitor (Su11274), Selleckchem (Houston, TX).

DNA cloning

SEMA3C:Fc, SEMA3C-FL and SEMA3C-AP and B1SP, D1SP and B1R4 were cloned into a modified lentiviral expression plasmid (pLB-U) previously described (Peacock et al, 2009). The human SEMA3C sequence Gly21 - Ser738 with the following amino acid substitutions (Arg551Ala, Arg552Ala, Arg611Ala and Arg612Ala) fused in frame with human IgG1e1-Fc (InvivoGen, San Diego, CA) was synthesized using flanking 5'-Xba1- attB1 and 3'-BamH1- attB2 sequences and ligated to pUC57 Genscript Corporation, (Piscataway, NJ). The SEMA3C:Fc sequence Full-length C-terminal 6-HIS tagged Human SEMA3C (NM_006379) was amplified by PCR using SEMA3C cDNA clone (SC116160; Origene Technologies, Rockville, MD) as template and attB1- and attB2-containing forward, (5'-GGGGACAAGTTTGTACAAAAAAGCAGGTTTCACCATGGCATTCCGGACAATTGC-3') and reverse, (5'-GGGGACCACTTTGTACAAGAAAGCTGGGTCCTAGTGATGGTGATGGTGATGC CCACCCCCTGACTCTGGCAACTGATTCC-3'), primers respectively and gateway cloned into pLBU. SEMA3C-AP: SEMA3C was amplified with forward Nhe1- (5'-CTACGCTAGCACCATGGCATTCCGGACAATTTG-3') and reverse BglII-(5'-CGGAAGATCTTGACTCTGGCAACTGATTCCTCC-3') primers using SEMA3C cDNA as a template for PCR. The amplified PCR product was restriction digested with Nhe1 and BglII and then ligated to pAPTag-5(GenHunter Corporation, Nashville, TN). B1SP: Human Plexin B1 semadomain and adjacent PSI domain (NM002673.5), linker and hinge sequence fused to human IgG1e1-Fc was synthesized with flanking 5'-EcoRV-

attB1 and 3'-EcoRV-attB2 sites and ligated to pUC57 by Genscript Corporation (Piscataway, NJ). D1SP: Human Plexin D1 semadomain and adjacent PSI domain (AY116661), linker and hinge sequence fused to human IgG1e1-Fc was synthesized with flanking 5'-EcoRV attB1 and 3'-EcoRV attB2 sites ligated to PUC57 by Genscript Corporation (Piscataway, NJ). B1R4: consisted of an extracellular fragment of Plexin B1 comprising N-terminal sema domain, 3 PSI and 3 IPT domains up to the first proprotease convertase cleavage site at position 1302 of the Plexin B1 similarly fused to a linker and human IgG1 Fc was similarly synthesisized and subcloned to pUC57. B1SP and B1R4 were then gateway cloned to pDONR201 entry vector (Invitrogen, Burlington, ON) and then pLB-U expression vector using Invitrogen Gateway cloning technology. All constructs were confirmed by DNA sequence analysis.

Generation of high-titre lentivirus

HEK293T cells were seeded on 15 cm tissue culture plates in DMEM containing 10% FBS (no antibiotics). Cells, at 80% confluence were transfected with vector alone (pLBU, $_{\text{EMPTY}}$), SEMA3C-FL, S3C-SD, SD-ALB, or, SEMA3C:Fc and packaging construct (pCMVΔR8.9) and pseudotyped with VSV G (Naldini et al, 1996) at ratio 1:1.3:4 in OptiMEM with polyethylenimine (23966; Polysciences Inc, Warrington,PA). DNA complexes were allowed to form for 20 minutes and then added to the cells. The culture medium was replaced 16 h later with 15 ml DMEM supplemented with 10% FBS. The media (lentiviral supernatant) was then collected and filtered (0.45µm) 30 h later lentiviral particles were concentrated by ultracentrifugation (100,000 x g) for 2.5 h at 4°C, pellets were resuspended in 1/100 volume culture medium. The lentivirus titre was determined by serial dilution of lentivirus and infection of HEK293T cells in medium containing 0.8 mg/ml protamine sulphate and blasticidin. Plates were stained after 1-2 weeks, in culture with 0.5% (W/V) solution Crystal Violet (Sigma-Aldrich, Mississuaga, ON). Lentiviral titres ranged from $5x10^6$-$8x10^8$ transducing units/ml.

Production of recombinant SEMA3C:Fc and B1SP and D1SP protein

CHO-S cells were transduced with SEMA3C-FL- or D1SP, B1SP-expressing lentivirus at 100 MOI. After 48h the cells were selected in media containing blasticidin (5µg/ml). Established populations were used to generate recombinant protein from conditioned medium. For SEMA3C-FL, cells (40 million) were seeded in Cellstack (2-stack) culture chambers (Corning Incorporated, Tewksbury, MA) in a volume of 200 ml DMEM, supplemented with 5% FBS (Life technologies, Gaithersburg, MD), heparin (25µg/ml) and blasticidin (5µg/ml). Conditioned media was collected after 48h and the replenished with the same medium containing 2% FBS. The CM was collected every 48h and replenished. Harvested CM was then centrifuged at 2000 RPM for 5 min and then filtered (0.22µm). Filtrates were purified over HisTrap excel (GE Healthcare, Uppsala, Sweden). Recombinant SEMA3C:Fc was then buffer exchanged and concentrated on an Amicon Ultra-15 centrifugal filter device (MWCO, 10kDa), to 2.0 mg/ml (Merck Millipore, Carrigtwolhill, Co, Cork, IRL). The protein sample was then desalted on a PD midiTrap G-25 column as directed by the manufacturer, (GE Healthcare, Buckinghamshire, UK), and then filter sterilized using a 0.22µm filter. For the production of B1SP, CHO-S B1SP cells (200 million) were seeded and cultured in a 5L Wave bag (GE Healthcare, Uppsala, Sweden) in 75%, Freestyle CHO expression medium/25% Optimem supplemented with 8mM Glutamine and Penicillin/Streptomycin (5.0ml/L), (Life technologies, Gaithersburg, MD) at 37°C and 8% CO_2. CM was harvested three times over a period of 4 weeks. Conditioned medium was centrifuged to remove the cells and then filter sterilized on 0.22µM PES filter units (Millipore, Darmstadt, Germany). Conditioned medium was purified on HiTrap MabSelect SuRe on an Akta Explorer Purified using the manufacturer's protocol (GE Healthcare, Uppsala, Sweden). Purified B1SP was buffer exchanged by dialysis using several 1L changes of PBS using snake skin dialysis membrane (MWCO 10kDa), followed by concentration to 4.0mg/ml on Amicon Ultra-15 filters (MWCO, 10kDa), (Thermo Scientific, Rockford,IL).

Quantitative RT-PCR

Total RNA was extracted from cultured cells and tumor tissues using TRIzol reagent (Invitrogen). Total RNA (2 µg) was reversed transcribed using random hexamers (Applied Biosystems, Foster City, CA) and 20U of Moloney murine leukemia virus reverse transcriptase (Invitrogen). Real time monitoring of PCR amplification of cDNA was performed using *SEMA3C* (Hs00170762_m1) and *GAPDH* (Hs03(9)29097_g1) (Applied Biosystems) on ABI PRISM 7900 HT Sequence Detection System (Applied Biosystems) with TaqMan Gene Expression Master Mix (Applied Biosystems, Burlington, ON). Target gene expression was normalized to *GAPDH* levels in respective samples as an internal standard. The results are representative of at least three independent experiments.

Cell stimulations, immunoprecipitation and immunoblotting

LNCaP cells were seeded at $4x10^5$ cells/well in 6-well plates in RPMI medium containing 10% FBS. The next day, the medium was replaced with RPMI minus phenol-red and serum starved for 24h. DU145 cells were treated similarly but were serum starved in DMEM for 4 h. The medium was changed again just prior to stimulation to remove endogenous secreted SEMA3C. For SEMA3C stimulations: cells were treated with SEMA3C:Fc for 10 min at the indicated concentrations or time points followed by a wash with PBS. For EGF stimulations, cells were first treated with ALS at the indicated concentration for 1 h followed by stimulation with EGF for 10 min. Cells were washed once with PBS. Cells were immediately lysed with 250 µL lysis buffer (50 mM Tris pH7.5, 150mM NaCl, 1% NP-40, 10 mM NaF, 10% Glycerol) containing Complete and PhosStop phosphatase protease inhibitors (Roche, Mississauga, ON). Whole cell lysates were centrifuged at 14,000 rpm for 20 min at 4°C. The protein concentration in cleared whole cell lysates was determined using the BCA kit (Thermo Scientific, Rockville, IL). For immunoprecipitations, DU145 cells (70% confluent on 10 cm dishes) were serum starved for 4 h in DMEM and then washed once and replenished with 6 ml serum-free DMEM prior to treatment with PBS or SEMA3C (0.5 µM) for 20 min at 37°C in 5% CO_2 The cells were washed with PBS and then lysed with 500µL xTractor lysis buffer (TakaRa Bio, MountainView, CA). Whole cell lysates (1.0 mg) were then immunoprecipitated with antibodies as directed by the manufacturers and incubated in final volume of 1.0 ml lysis buffer overnight with rotation at 4°C. Immunocomplexes were incubated with 40µL protein A/G agarose beads (SantaCruz Biotechnology, SantaCruz Ca) for 1 hr with rotation at room temperature. Immuncomplexes and beads were then washed 3 times 10 min with 1.0 ml lysis buffer. The immunocomplexes were then boiled in (35µL) 2x Laemmli buffer before SDS-PAGE. Protein lysates (20-50µg) were analyzed by SDS-PAGE (8-10%) and transferred by Western Blot to nitrocellulose membranes (Bio-Rad, Hercules, CA). Membranes were blocked in PBS containing 5% BSA, or TBS Odyssey buffer (Licor, Lincoln, NE) for probing with phospho-antibodies or 5% non-fat milk for total and loading control antibodies as directed by the manufacturers. Membranes were washed 3X with TBST followed by incubation with the appropriate HRP -or IRdye -conjugated-secondary antibodies for 1h. Blots were washed again 3X for 5 min with TBST. Detection was by ECL or by image analysis using a LICOR infrared imager and Image Studio Lite version 3.1, software. Densitometry analysis of EGFR phosphorylation was performed using Syngene Gene Tools version 4.02 (Synoptics Ltd., Cambridge, UK).

Tissue Microarray, (TMA) Immunostaining. TMA's were designed by spotting two 0.6 mm tissue cores per case, obtained from formalin fixed and paraffin embedded archival tumor blocks and constructed using a Beecher microarrayer. Sections were deparaffinized and rehydrated through xylene and ethanol then transferred to the 0.02% triton-X100 for permeabilization. Slides in citrate buffer (pH 6.0) were heated in steam for 30 min. After cooling for 30 min and 3 x 5 min washes in PBS, the slides were incubated in 3 % BSA for 30 min. The slides were transferred to 3% H_2O_2 for 10 min and then incubated overnight with primary antibodies to CD31, Ki67, and TUNEL at 1:50-100, concentration in 1% BSA. The primary antibody was than washed with PBS. Specific protein staining was determined using the LSAB+kit system (Dako, Carpinteria, CA). The Chromogen Nova-red (Vector Laboratories, Burlingame, CA) was applied for 2 min and counterstaining was performed with H&E (Vector Laboratories, Burlingame, CA). After ethanol rehydrating, a coverglass was applied with Cytoseal, a xylene based mounting media (Stephen Scientific, Riverdale, NJ). Negative control slides were processed in an identical fashion to that above, with the substitution of 1% BSA for the primary antiserum. Photomicrographs were taken with a Leica DMLS microscope coupled to a digital camera (Photometrics CoolSNAP, Roper Scientific, Inc., Glenwood, IL). The staining intensity of tissue was evaluated and scored by a single pathologist (Ladan Fazli). Specimens were graded from 0 to 3- intensity, representing the range from no staining to heavy staining by visual scoring and automated quantitative image analysis by pro-plus-image software (Media Cybernetics, Carlsbad, CA). All comparisons of stain intensity were made at x200 magnification.

Plexin B1 and SEMA3C silencing

Two siRNAs targeting Plexin B1 (siPLXNB1-1 and siPLXNB1-2) were used. siPLXNB1-1 was validated (Hs_PLXNB1_6, Qiagen, Montreal,PQ). siPLXNB1-2 was previously validated by (Swiercz et al, 2008) and was synthesized (Thermo Scientific, Rockford, IL). LNCaP cells were transfected with either scramble siRNA (siScr) (AUCAAACUGUUGUCAGCGCUGUU), siPLXNB1-1 (CCGGGUGGAAUUUAUCCUUGAUU), or, siPLXNB1-2 (ACCACGGUCACCCGGAUUCUU) (10 nM) using HiPerFect reagent (Qiagen, Montreal PQ) as described by the manufacturer. Transfected cells were incubated for 72 hours and then incubated in the presence or absence of SEMA3:Fc (100nM) for 10 minutes. Cells were washed and then lysed. Plexin B1 levels were analyzed from lysates (30 µg) by PAGE. Western Blots were probed with anti-Plexin B1 and anti-phospho-SHC. Blots were reprobed with anti- vinculin and anti-SHC as loading control. DU145 cells were transfected with scramble siRNA, siPLXNB1-1 or siPLXNB1-2 (10nM) using RNAiMax reagent as directed by the manufacturer, (Invitrogen,

Missisauga,On). Following 72-hour incubation the cells were replated at 20,000 viable cells/well in a 96-well flat bottom plate and let adhere for 4 hours. The remaining cells were lysed and assayed for Plexin B1 expression by Western blot. The blot was reprobed with vinculin as loading control. Medium was replaced with DMEM without serum for 16 hours prior to the binding assay. Cells were then washed once with HBHA [20mM HEPES, 150mM NaCl, 5mM CaCl2, 1mM MgCl2], buffer containing, 0.5% BSA. The Opti-MEM1-conditioned medium from 293T cells expressing either the APTag vector alone or, the SEMA3C-AP construct was assayed in a PNPP assay to find the linear and equivalent range of AP-enzymatic activity. The SEMA3C-AP was applied by diluting an equal volume of SEMA3C-AP CM to an equal volume of HBHA buffer that gave an enzymatic activity of 0.877. The APTag was first diluted 1:4 in Opti-MEM and then an equal volume of the 1:4 diluted- APTag CM was diluted to an equal volume of HBHA. The enzymatic activity of AP-Tag CM was 1.069. The binding assay was performed as previously described (Flanagan & Leder, 1990). Endogenous phosphatase heat inactivation was done in HBHA at 65°C following the fixation step. Plate readings at 562 nm were taken initially and then every 24 hours. The data was done in quadruplet. The binding of the APTag vector CM was subtracted from the binding to SEMA3C-AP in each case. siRNA targeting SEMA3C (siSEMA3C-1, s20598, CACCAUCCUUUAGACUACAtt) and (siSEMA3C-2, s20599, GAAUCAUGCUCCUAUAACAtt) were validated siRNA sequences (Ambion, Life Technologies Corporation, Carlsbad, CA). DU145 cells were reverse transfected using RNAiMax with siRNA scramble, siSEMA3C-1 or-2 or, si-PLXND1 (Dharmacon, J-014121-10), si- PLXNB1-1 and si-NRP1(Dharmacon, J-019484-06), si-NRP2 (Dharmacon, J-017721-06), alone or in combination in 6-well plates at a cell density of 3.4 x 10^4/ml in DMEM supplemented with 5% FBS. After 24-48-hour incubation, the cells were scraped and lysed or replated at a density of 2000 cells/well on 96-well plates in DMEM medium containing 0.5% FBS and SEMA3C (100nM) for 48 hr. Cell growth was assayed using crystal violet staining. Protein lysates (30-50µg) were loaded on SDS-PAGE. Gels were transferred by Western blot and immunoblotted. For analyses, siRNA transfected cells were incubated for 48-72h. For rescue of growth inhibition by SEMA3C silencing, cells were reverse transfected with siScr, siSEMA3C-1 or siSEMA3C-2 siRNA as described in the signaling experiment. Twenty-four hours later the cells were trypsinized and 100µL cells were seeded in 96- wells (2 X 10^4 /ml) in the presence or absence of SEMA3C:Fc (1.0 µM). The media containing fresh SEMA3C:Fc was changed daily for 4 days. Cell proliferation was monitored over 4 days using the Presto Blue cell viability reagent (Life Technologies Corporation, Carlsbad, CA). Fluorescence was measured at excitation 535 nm and emission 612 nm using a Tecan F500 plate reader (Tecan, Austria, GmbH). The endogenous knock down of SEMA3C was monitored at 48 hours after transfection by Western blot.

Treatment with SEMA3C ASO

The SEMA3C ASO used in this study was modified at the 2' position of the sugar with a 2'-O-(methoxyethyl) group on the five bases at both the 5'- and 3'-termini (Isis Pharmaceuticals, Carlsbad, CA). The central 10 bases contained deoxyribose and the entire oligonucleotide backbone contained phosphorothioate linkages. We designed an antisense oligonucleotide (ASO) against nucleotide positions 1-20 of the SEMA3C coding sequence. The sequence of SEMA3C ASO used was 5'-CAAATTGTCCGGAATGCCAT-3'. The scramble oligonucleotide (Scr) used was designed not to match any mRNA in the human or mouse transcriptomes and has the sequence 5'-CCTTCCCTGAAGGTTCCTCC-3'. Oligofectamine (Invitrogen), a cationic lipid, was used as a transfection reagent. LNCaP, C4-2, and DU145 cells (7 x 10^5) were seeded on 10 cm dishes. Twenty-four hours later, cells were transfected with ASOs. Briefly, the indicated doses of ASOs were diluted in Opti-MEM I serum-free medium and Oligofectamine reagent (Invitrogen, Carlsbad, CA), mixed and incubated at room temperature for 20 min prior to addition to cells. The media was changed after 4-6 h. For Rescue of SEMA3C ASO treated cells. LNCaP cells were transfected with Scr ASO or SEMA3C ASO (100nM), 24h later cells (6000/well) were plated on 96-well plates in media containing either PBS or, SEMA3C:Fc (0.5 µM). Cell growth was assayed initially following 4 hr incubation and after 48 hr using Presto Blue proliferation reagent.

***In vitro* cell growth assays.** Cell growth of DU145 and LNCaP cells was done by direct counting using trypan blue exclusion. The cell growth C4-2, 22Rv1, LNCAP and MR49F were also assessed in 48-well plates using either CyQuant or Presto Blue (Invitrogen) according to manufacturer's protocol. The fluorescence was determined with a micro-plate reader (Fluoreskan Ascent FL, Thermo Scientific) at 485/527 nm. The results are representative of at least three independent experiments. Alternatively, LNCaP (3x 10^4/ml) were plated in 96-well flat bottom tissue culture plates. Proliferation was assessed 4 days later in response to ALS treatment in the presence or absence of SEMA3C:Fc (S3C:Fc), or R1881 using the Presto Blue proliferation viability reagent (Life Technologies Corporation, Carlsbad, CA).

Cytotoxicity assay. The cytotoxicity assay was performed as previously described (2, 3, 8). Briefly, C4-2, 22Rv1 and MR49F cells were seeded in 48-well plates, and on the following day, cells were transfected with scramble or, SEMA3C ASO or, the indicated concentrations of anticancer agents (MDV and docetaxel) were applied. After 48 h, the surviving cells were stained using the crystal violet. The absorbance was determined with a micro-plate reader (Epoch, BioTek, Winooski, VT) at 560 nm. The results are representative of at least three independent experiments.

Sub-Go/G1 DNA content assay

Flow cytometry analysis of propidium iodide-stained nuclei was performed, as described previously. Briefly, LNCaP, C4-2, 22Rv1, or $LNCaP_{empty}$ and $LNCaP_{SEMA3C\text{-}FL}$ cells were seeded in 10 cm dishes, and on the following day, cells were either treated with ASO as described above or incubated in medium containing 10% FBS alone or in the presence of vehicle, ENZ or docetaxel. The cells were trypsinized 48 h after treatment, washed in 1.0 ml PBS and then fixed in 1.0 ml 80% ethanol overnight at 4˚C. Fixed cells were incubated in DNase-free RNase A 50 µg/ml and propidium iodide (50 µg/ml) at 4°C for 1 h. The stained cells were analyzed for relative DNA content on a Becton Dickenson FACS Canto II flow cytometer (BD Biosciences, Mississauga, ON). The results are representative of at least three independent experiments.

Proximity ligation assay

The Proximity Ligation Assay (PLA) enables the detection of proximal binding of protein interactions on the cell surface. The target is detected using target-specific primary antibodies raised in different host species. Detection is performed *in situ* using secondary antibody probes linked to complementary oligonucleotide sequences of approximately 40 nucleotides that can only ligate when in close proximity. A Ligation step consisting of two oligonucleotides and Ligase results in hybridization of the probes. The oligonucleotide arm of one of the PLA probes provides the primer for rolling circle amplification incorporating a fluorescent conjugated nucleotide that generates a concatemeric product. The PLA signals are visualized as distinct fluorescent spots that are analyzed using fluorescent confocal microscopy and analyzed using Duolink Imagetool software (Olink Bioscence,Uppsala,Sweden).

For DU145 (45,000) or LNCaP (60,000) cells were seeded on 1 cm diameter coverslips in 24 well plates the day before treatment. Treatment was done with either SEMA3C:Fc (0.5 µM) or, $rhIgG_1Fc$ (0.5 µM) as control, in HBHA binding buffer containing 2% FBS for one hour on ice. Cells were then washed three times with HBHA/2% FBS buffer followed by fixation for 20 min at RT in 4% paraformaldehyde. Fixed cells were then diluted with 1.5 ml ddH_2O. Fixed cells were then permeabilized with 0.5% triton X-100 for 30 min. prior to the PLA assay. EGF treatment was done at 10 ng/ml for 20 min. incubation in 5% CO_2 at 37^{0}C.

The Proximity Ligation Assay (PLA) was performed according to the manufacturers protocol with Duolink In-Situ Fluoresence kit (Sigma-Aldrich, St Louis, MO), to analyze the interaction between Plexin B1-HER2, Plexin B1-EGFR, Plexin B1-MET, SEMA3C-NRP1, SEMA3C-Plexin B1 and Plexin D1-HER2. PLA associations were identified by confocal microscopy. PLA signals were quantified using Duolink software and analyzed on Graphpad-Prism software. Data was statistically tested using KRUSKAL WALLIS or by Mann Whitney test.

Appendix Figure S1

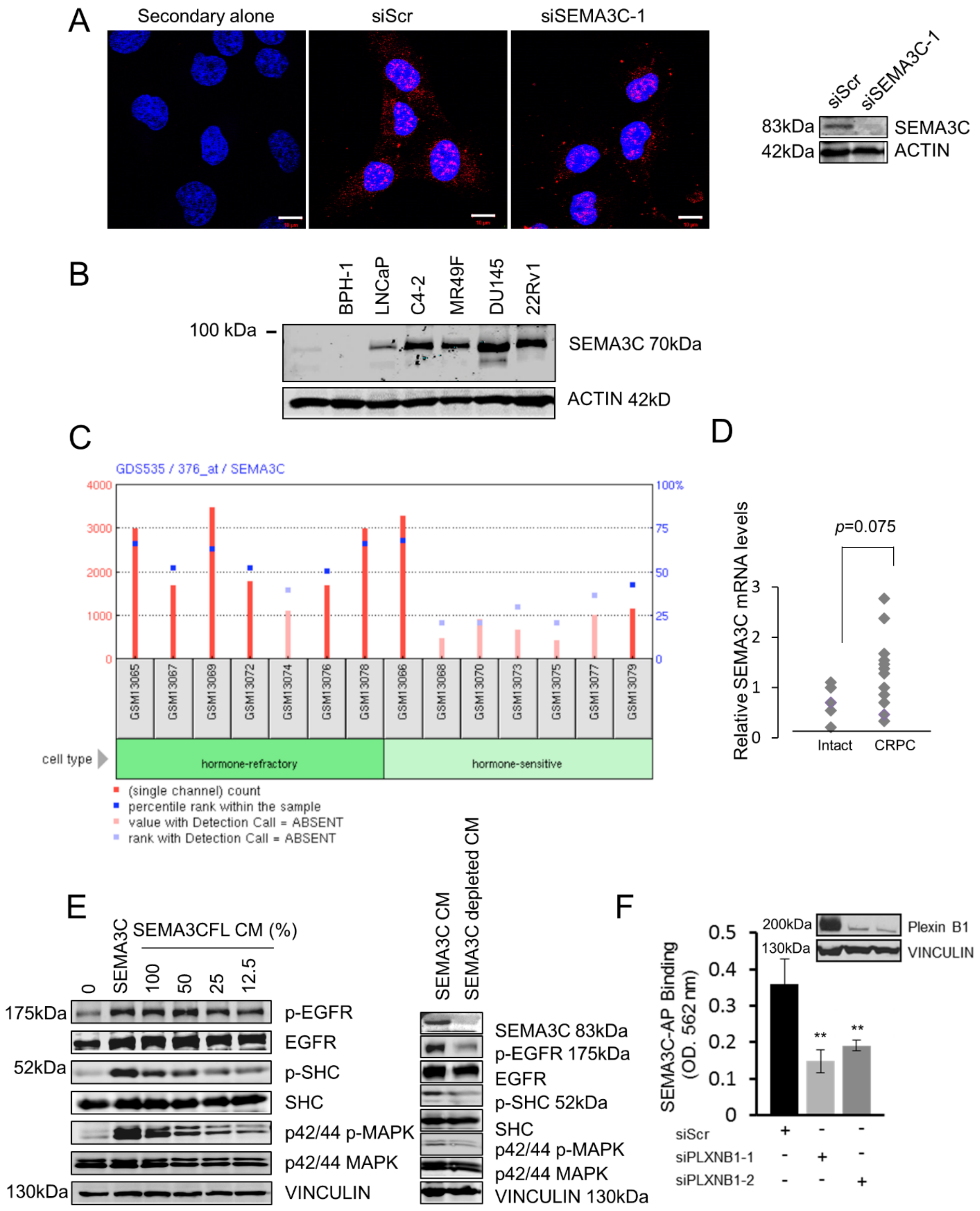

Appendix Figure. S1. High SEMA3C expression is associated with castrate resistant tumors. **(A)** Confocal images of SEMA3C expressed in DU145 cells transfected with siRNA scramble (siScr) or siSEMA3C. DU145 cells stained with secondary antibodies alone (left panel)

or SEMA3C (N20) antibodies. Knock down of SEMA3C protein levels is shown. Scale bar: 10µm. **(B)** Secreted forms of SEMA3C protein in PCa cell-lines. **(C)** High SEMA3C expression is associated with castrate resistant tumors. Chen et al, 2004 performed microarray experiments investigating differentially expressed genes in a panel of isogenic hormone-sensitive and castration resistant xenograft tumor pairs. Higher SEMA3C expression was found in castration resistant xenograft tumors as compared to isogenic hormone-sensitive pairs in 6 out of 7 cases. http://www.ncbi.nlm.nih.gov/geoprofiles?term=GDS535+AND+376_at. [Samples: GSM13065: CWR22HR, GSM13067: LAPC4HR, GSM13069: LAPC9HR, GSM13072: LUCaP23HR, GSM13074: LUCaP35HR, GSM13076: LUCaP41HR, GSM13078: LNCaP-HR, GSM13066: CWR22HS, GSM13068: LAPC4HS, GSM13070: LAPC9HS, GSM13073: LUCaP23HS, GSM13075: LUCaP35HS, GSM13077: LUCaP41HS, GSM13079: LNCaP-HS] **(D)** SEMA3C mRNA levels from intact (control) and CRPC (LNCaP) xenograft tumour tissue, p=0.075, determined by qPCR. **(E)** LNCaP cells treated with either recombinant SEMA3C-Fc fusion protein (0.5µM), or conditioned medium from HEK 293 T cells alone (0) or, mixed in the indicated proportion with conditioned medium (CM) from HEK 293T that stably overexpress and secrete natural Full-length SEMA3C. The immunoblot shows the levels of EGFR, SHC and MAPK phosphorylation after 20 minutes of treatment. Vinculin levels are shown as loading control (left panel). The right panel shows SEMA3C levels and EGFR, SHC and MAPK phosphorylation levels in DU145 cells treated with CM as above or SEMA3C immuno-depleted using anti-SEMA3C N20 (2µg/ml) CM for 20 min. Vinculin levels are shown for loading control. **(F)** SEMA3C-AP binding assay on DU145 cells transfected with siScr, siPLXNB1-1, p= 0.004, or siPLXNB1-2, p=0.007. Top panel shows Plexin B1 and vinculin protein levels. Bottom panel shows SEMA3C-AP binding to siRNA transfected DU145 (mean +/- SEM), (n=4), statistical analysis was performed using the two-tailed unpaired student's t-test.

Appendix Figure S2

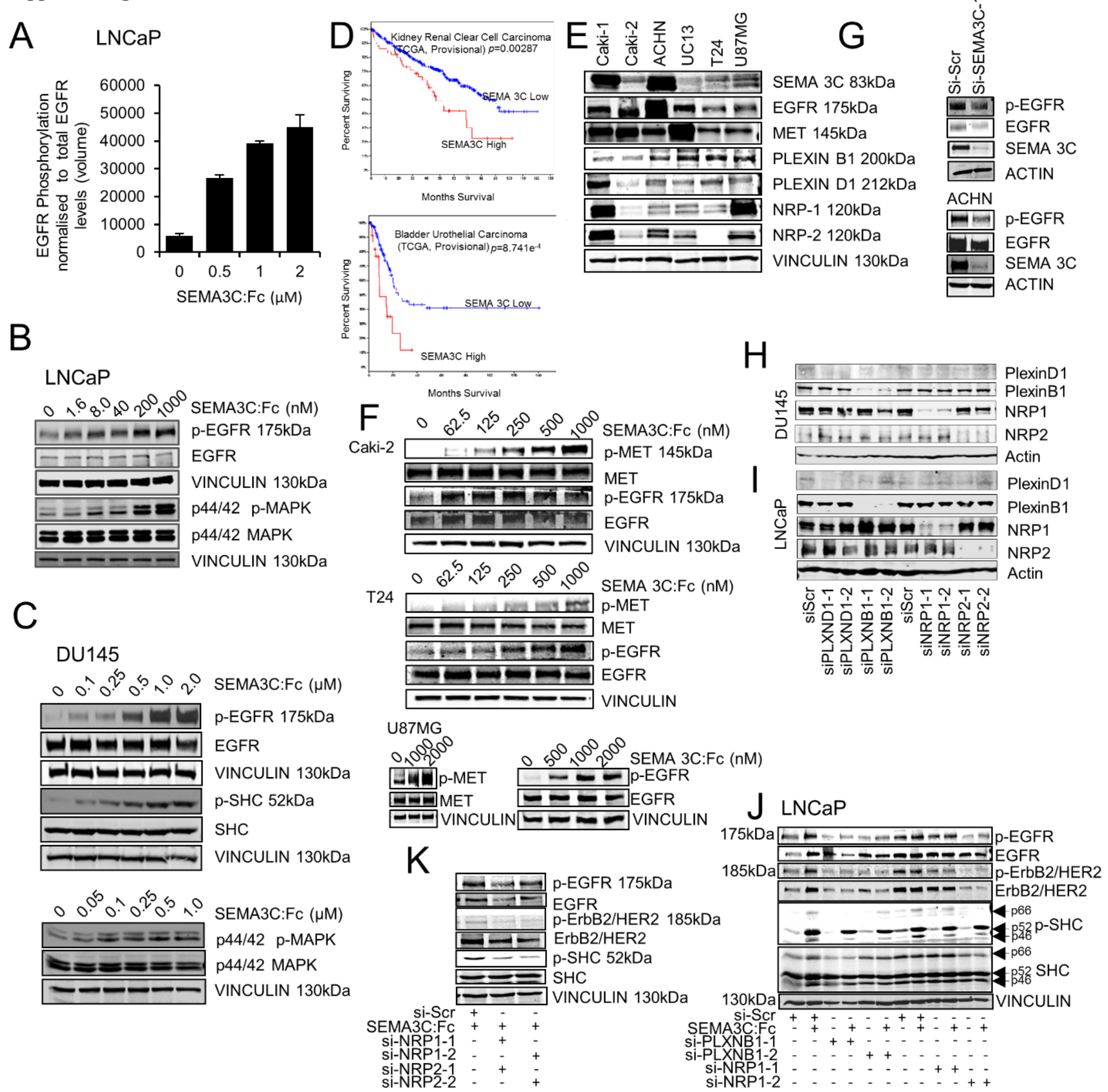

Appendix Figure. S2. SEMA3C drives EGFR and MET signaling in a spectrum of cancer cell lines. (A) Densitometric analysis of EGFR phosphorylation (mean +/- SEM) induced by SEMA3C:Fc stimulation of LNCaP cells from 3 independent experiments. Increased phosphorylation of EGFR and downstream signaling proteins SHC and/or MAPK in LNCaP (B) and DU145 (C) cells treated with varying concentrations of SEMA3C:Fc (0-2 µM) for 20 min. Levels of indicated phosphoproteins and total proteins were assessed by immunoblot analyses. Vinculin is shown as loading control. (D) SEMA3C expression survival analysis of Kidney Renal Clear Cell Carcinoma, p=0.00287 and Bladder Urothelial Carcinoma, p=8.7e^{-4}, (cBioPortal). (E) Expression levels of SEMA3C, EGFR, MET, PLEXIN B1, PLEXIN D1, NRP-1 and NRP-2 in cell lines: Kidney (Caki-1, Caki-2, ACHN), Bladder (UC13, T24) and Brain (U87MG). (F) Cell-lines, Kidney, Bladder and Glioblastoma that expressed lower levels of

SEMA3C were serum starved and then treated with SEMA3C:Fc (0-2.0µM). SEMA3C-mediated phosphorylation of EGFR and MET is shown. Blots were reprobed with EGFR, MET or Vinculin as loading control. (G) Cancer cell lines from Kidney that expressed high levels of SEMA3C were transfected with si-Scr or si-SEMA3C-1 siRNA. Western blots show the phosphorylation levels of EGFR. Specific knock down of SEMA3C is shown. Blots were reprobed with EGFR antibodies and Actin as loading controls. (H) Immunoblot showing expression levels of Plexin D1, Plexin B1, NRP1 and NRP2 in DU145 and (I) LNCaP cells following siRNA mediated silencing. (J) si-RNA knock down of Plexin B1, NRP1 and NRP2 in LNCaP cells inhibits SEMA3C-induced phosphorylation of EGFR, HER2/ErbB2 and SHC (K) siRNA knockdown of NRP1 and NRP2 together in LNCaP cells inhibits EGFR, HER2/ErbB2 and SHC phosphorylation.

Appendix Figure S3

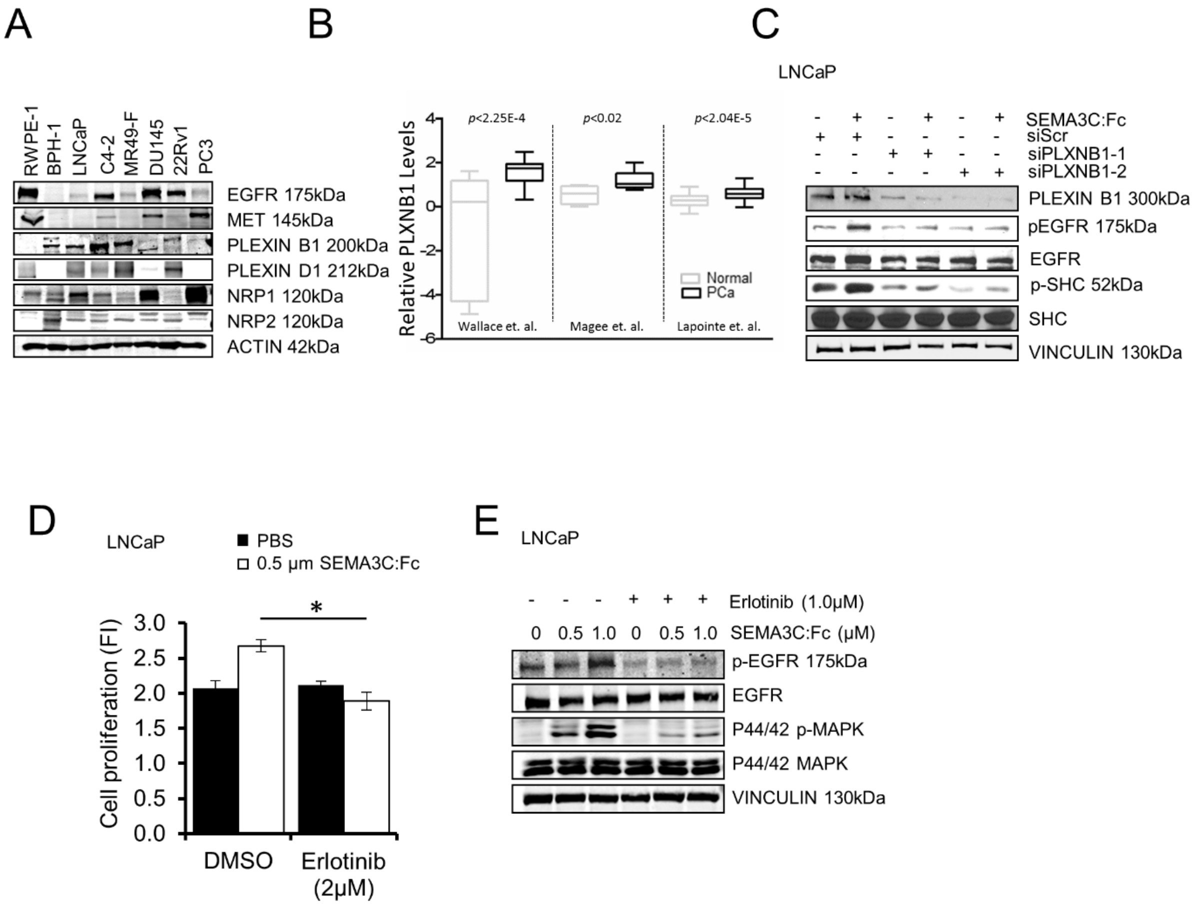

Appendix Figure. S3. **SEMA3C-induced receptor tyrosine kinase signaling is mediated through Plexin B1**. (A) Immunoblot showing the relative expression levels of EGFR, MET, PLEXIN B1, PLEXIN D1, NRP1 and NRP2. Actin levels are shown as loading control. (B) Analyses of independent microarray analyses were determined using the Oncomine resource. Relative Plexin B1 expression levels of PCa compared to normal prostate tissue and *p*-values are shown. (C) LNCaP cells transfected with two independent PLEXIN B1 siRNAs or si-scramble (siScr) control. Seventy-two hours later, cells were treated with vehicle control or SEMA3C:Fc. Immunoblots were probed with antibodies against Plexin B1, pEGFR, EGFR, pSHC and SHC. The blot was reprobed with Vinculin antibodies as loading control. (D) Cell proliferation of LNCaP treated with DMSO or Erlotinib (2 μM) and PBS or SEMA3C:Fc for 48hr. Bars represent mean, Fluorescence Intensity (FI) normalized to day 0 performed in triplicate, +/- SEM, *$p<0.05$. (E) LNCaP cells were treated with Erlotinib. Immunoblots show EGFR and MAPK phosphorylation in LNCaP cells. Blots were reprobed with EGFR and MAPK and Vinculin as loading controls.

Appendix Figure S4

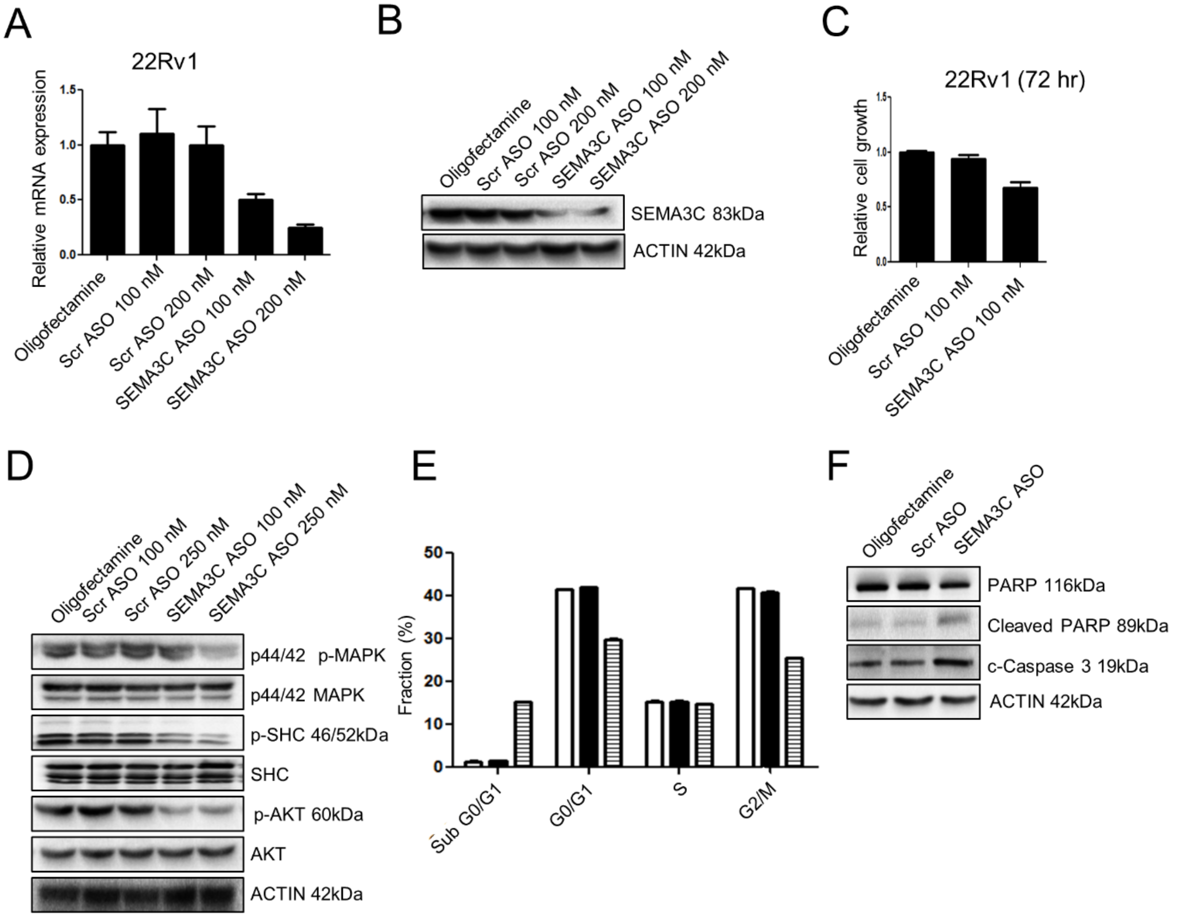

Appendix Figure. S4. SEMA3C ASO suppresses growth of castration resistant CWR22Rv1 cells. SEMA3C mRNA, (**A**) and protein levels, (**B**), were determined by qPCR and immunoblot analyses respectively in CWR22Rv1 cells transfected with varying concentrations of Scr or SEMA3C ASO at the indicated doses. (**C**) Levels of MAPK, SHC and Akt phosphorylation were determined by immunoblot analyses of cell lysates from CWR22Rv1 cells transfected with Scr or SEMA3C ASO at the indicated concentrations. (**D**) Growth of CWR22Rv1 cells treated with oligofectamine, 100 nmol/L of Scr ASO or SEMA3C ASO was determined at 72 h post-transfection by direct cell counting (**E**) Cell cycle analyses in CWR22Rv1 cells treated with oligofectamine (white bars), 100 nmol/L of Scr ASO (black bars) or SEMA3C ASO (hatched bars) was determined by propidium iodide staining and flow cytometry. The cell-cycle fraction is shown. Bars, mean ±SD, **p* < 0.05. (**F**) Levels of indicated proteins were assessed by immunoblot analyses of lysates from CWR22Rv1 cells treated with oligofectamine, 100 nmol/L Scr ASO or SEMA3C ASO.

Appendix Figure S5

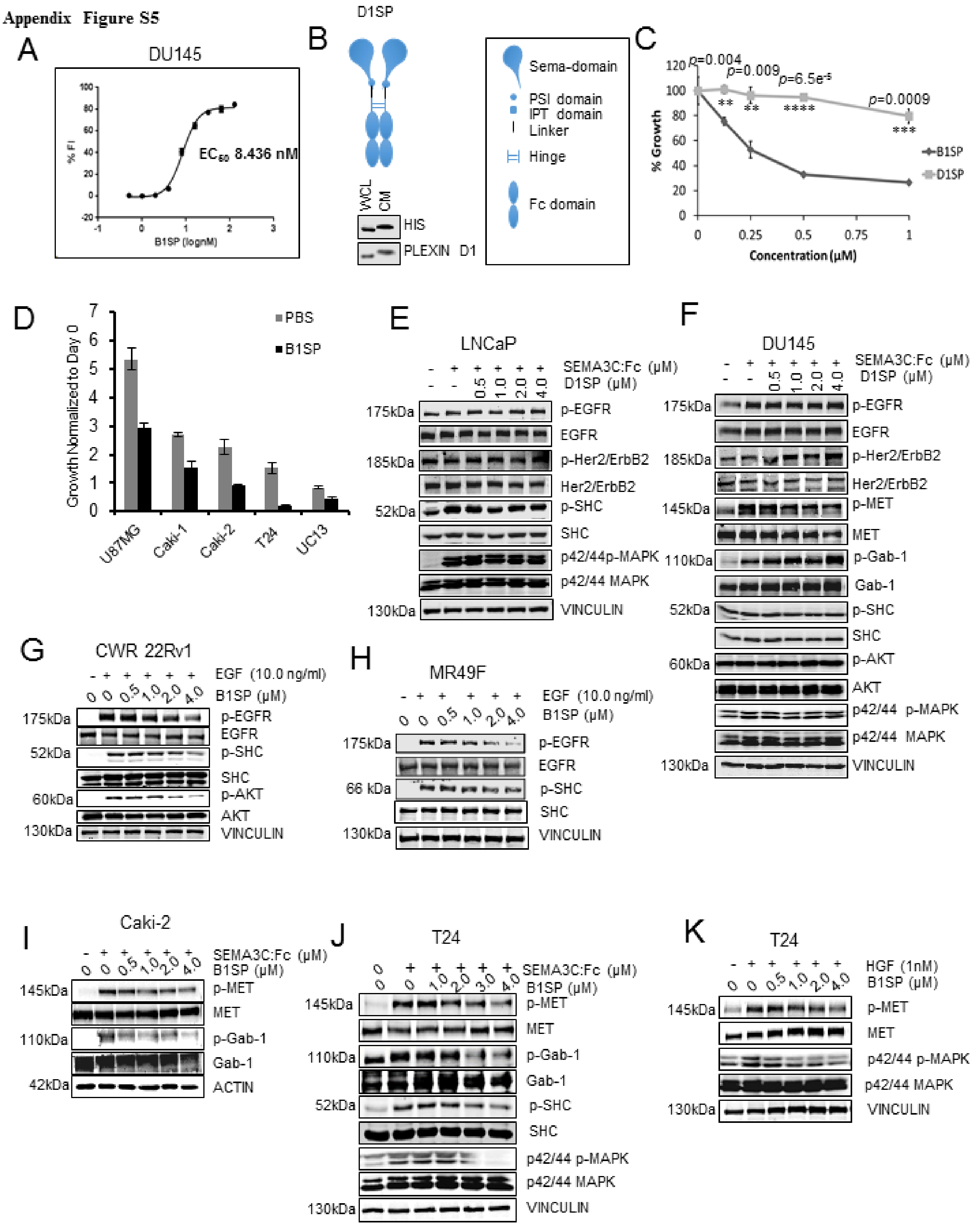

Appendix Figure. S5. Plexin B1 Decoy protein inhibits Cell Growth, Semaphorin and RTK signaling and Tumor growth *in vivo*. (A) Dosage dependent cell surface binding of PE-conjugated B1SP in DU145 cells as determined by flow cytometry. **(B)** Schematic of HIS-tagged

D1SP:Fc fusion protein and immunoblot showing the expression of D1SP from CHOs cell lysates and conditioned medium. The immunoblot was probed with anti-HIS and Plexin D1 antibodies. **(C)** Cell growth of LNCaP cells treated with either B1SP or D1SP at the indicated concentrations for four days. Cell growth was assayed using the Prestoblue cell proliferation reagent. Data represents the Mean and SEM of triplicate wells. The data is representative of three independent experiments. **$p<0.004$, ***$p=0.0009$, ****$p=6.5e^{-5}$. **(D)** Cell growth of a variety of cancer cell-lines treated with B1SP (2µM) or PBS control for 4 days. **(E)** LNCaP cells treated with D1SP (0-4.0µM) in the absence of serum for 1 hour. Cells were then washed and treated with SEMA3C:Fc (0.5µM) for 20 min. The immunoblots show the phosphorylation levels of EGFR, Her2/ErbB2, SHC and MAPK. The blots were reprobed with EGFR, HER2/ErbB2, SHC and MAPK and vinculin antibodies for loading controls. **(F)** DU145 cells treated with D1SP (0-4.0µM) in the absence of serum for 3 hours. Cells were then washed and treated with SEMA3C:Fc (0.5µM) for 20 min. The immunoblots show the phosphorylation levels of EGFR, Her2/ErbB2, MET, Gab-1, SHC, AKT and MAPK. The blots were reprobed with EGFR, HER2/ErbB2, MET, Gab-1, SHC, AKT and MAPK and vinculin antibodies for loading controls. The blots are representative of three independent experiments. **(G)** Dose dependent inhibition of EGFR signaling in CWR22Rv1 cells treated with B1SP. **(H)** Dose dependent inhibition of EGFR signaling in MR49F cells treated with B1SP. **(I)** Dose dependent inhibition of SEMA3C-induced MET activation in Caki-2 cells treated with B1SP. **(J)** Dose dependent inhibition of SEMA3C-induced MET, Gab1, SHC and MAPK phosphorylation in B1SP treated T24 cells. **(K)** Dose dependent inhibition of HGF-induced MET activation in B1SP-treated T24 cells.

Appendix Figure S6

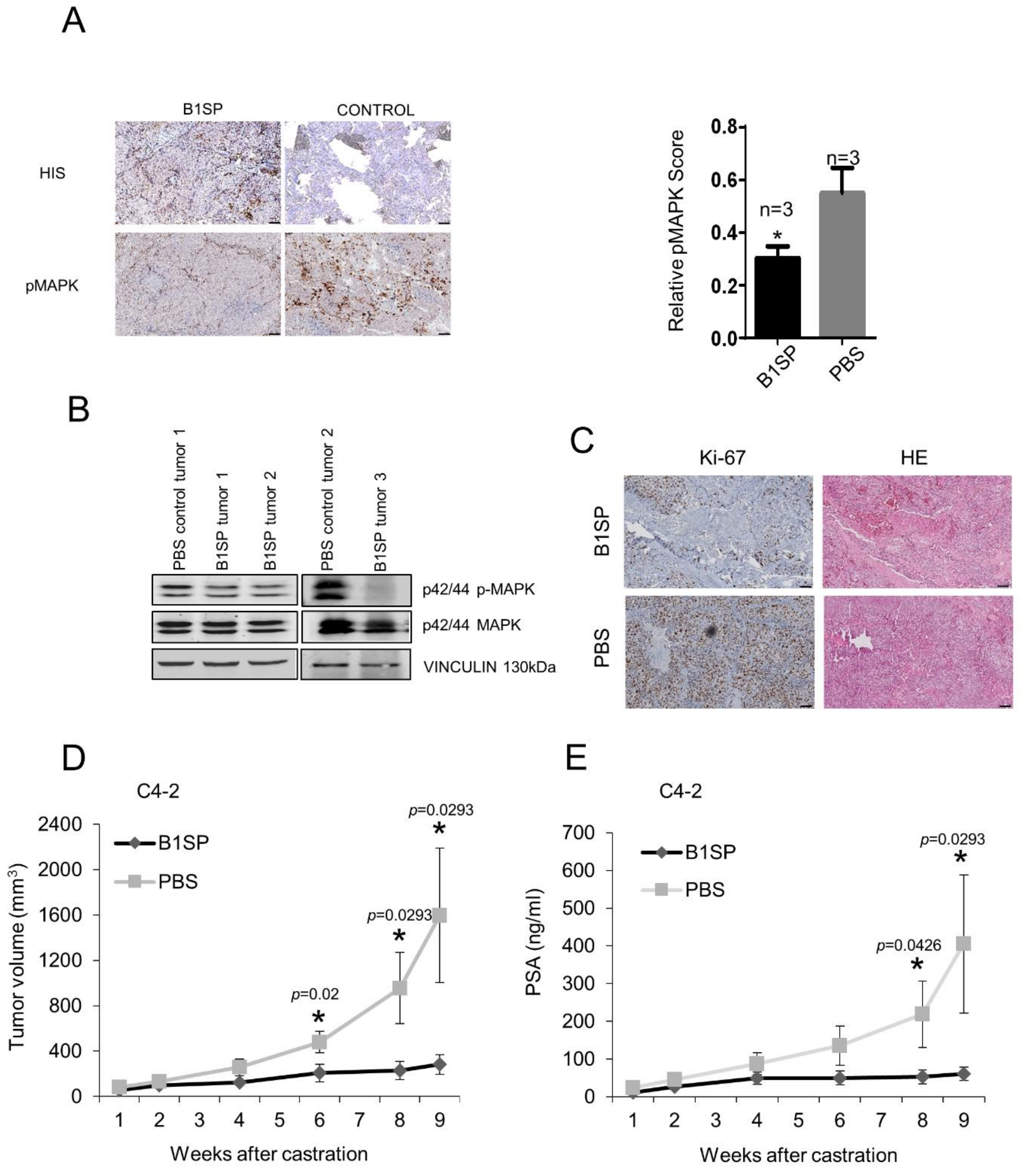

Appendix Figure. S6. (A) IHC staining of tissue sections derived from LNCaP xenograft tumors isolated from PBS and B1SP-treated mice,(n=3). B1SP was detected by staining with HIS antibodies, the phosphorylation levels of MAPK are shown, *p*=0.032. **(B)** Immunoblot showing MAPK phosphorylation levels from tissue homogenates isolated from PBS-control and B1SP-

treated mice bearing Xenograft tumors. The immunoblots were reprobed with MAPK and vinculin antibodies for loading controls. **(C)** IHC showing cellularity (H&E) and proliferation (Ki-67) in tissue sections derived from PBS- and B1SP-treated mice bearing LNCaP xenograft tumors. **(D)** Tumor volume (mm^3) and **(E)** PSA (ng/ml) of from athymic *nu*$^{-/-}$ mice bearing C42 Tumors treated with either PBS (n=5) or B1SP (n=7) post castration over a period of 10 weeks. Data are representative of Mean +/- SEM, *p<0.05. Statistical analysis was performed using the Mann Whitney test. Scale bar: 100µm.

Appendix Table S1

Ranking	Description	Phospho site human
1	PKBa (Akt1)	S473
2	CDK1/2	Y15
3	Nek2	Pan-specific
4	EGFR	Y1148
5	MEK6 (MKK6)	Pan-specific
6	Syk	Pan-specific
7	Cdc25B	Pan-specific
8	RSK1	Pan-specific
9	4E-BP1	S65
10	Caveolin 2	Pan-specific
11	Rb	S608
12	Hsc70	Pan-specific
13	IR/IGF1R	Y1189/Y1190
14	ErbB2	Y1248

Appendix Table S1. Kinex antibody microarray detection of a number of proteins and phospho-proteins that were coordinately modulated in an inverse manner with gain and loss of SEMA3C. The protein list was created by comparing protein levels as determined by immunoblot analyses and identifying top ranking proteins/phosphoproteins that were upregulated when SEMA3C was overexpressed ($LNCaP_{EMPTY}$ versus $LNCAP_{SEMA3C}$, n=2 biological replicates) and were correspondingly downregulated when SEMA3C was silenced (siScr vs siSEMA3C-1, n=2). Of interest, levels of phosphoproteins in the Erb family RTK signaling pathway (ErbB2, and EGFR), the PI3K/PTEN cell survival pathway proteins (Akt1, and 4EBP1), and the cell cycle regulatory proteins (Rb, and CDK1/2) were found to be induced by SEMA3C.

Appendix Table S2

Type of Cancer	Cell Line	IC_{50} (μM)
Prostate	LNCaP	0.2509
	C42	0.9636
	DU145	0.1455
	22RV1	0.1952
Glioblastoma	U87MG	2.965
Kidney	Caki-1	2.215
	Caki-2	0.1725
Bladder	T24	0.06164
	UC13	0.84

Appendix Table S2. The IC_{50} (μM) values of various Prostate, Kidney, Bladder and a glioblastoma cell lines. The data is representative of cell proliferation assays performed in triplicate. Each cell line was assayed in three independent experiments. IC_{50} values were determined using GraphPad Prism software.
