# Supplementary material for: SEMA3C drives cancer growth by transactivating multiple receptor tyrosine kinases via Plexin B1
Source: EMBO Mol Med. 2018 Jan 18;10(2):219–38. doi: 10.15252/emmm.201707689 (PMC5801490; doi:10.15252/emmm.201707689)

Appendix Figure S2B

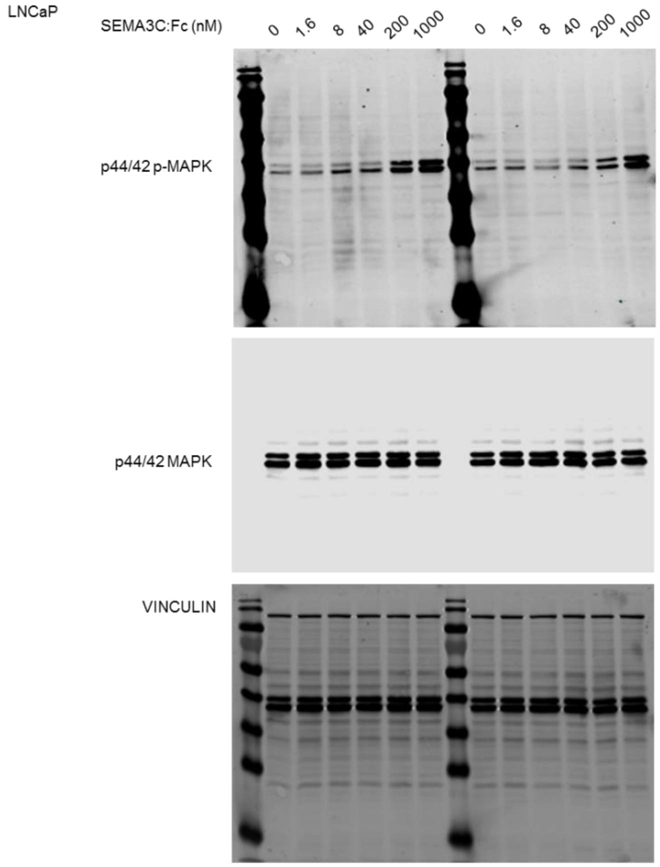

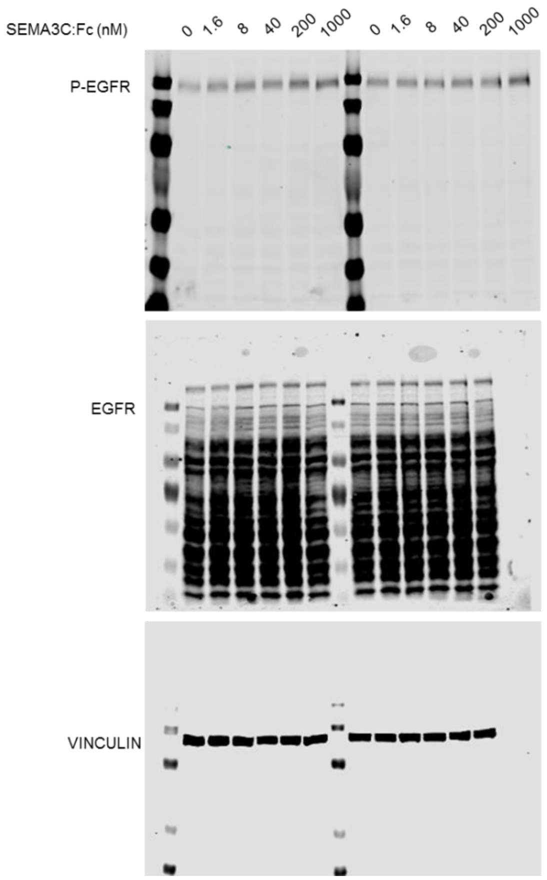

Appendix Figure S2C

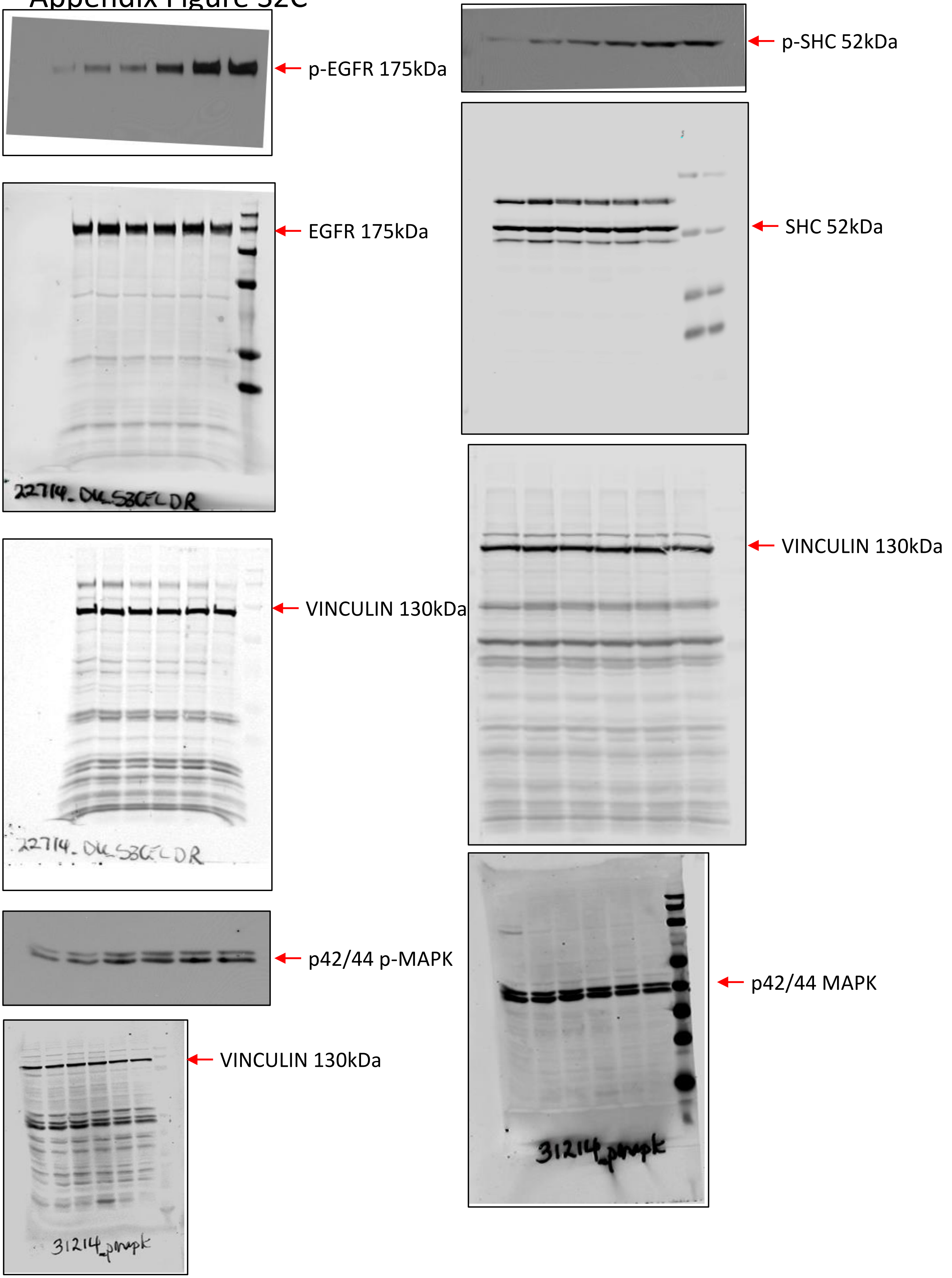

Appendix Figure S2E

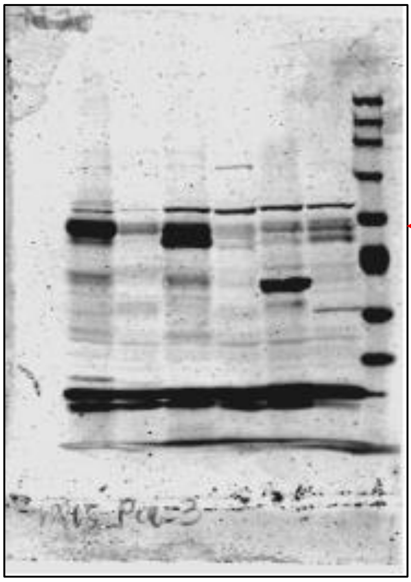

SEMA3C 83kDa

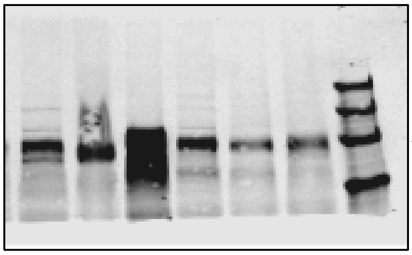

EGFR 175kDa

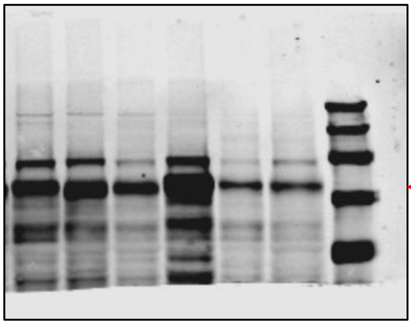

MET 145kDa

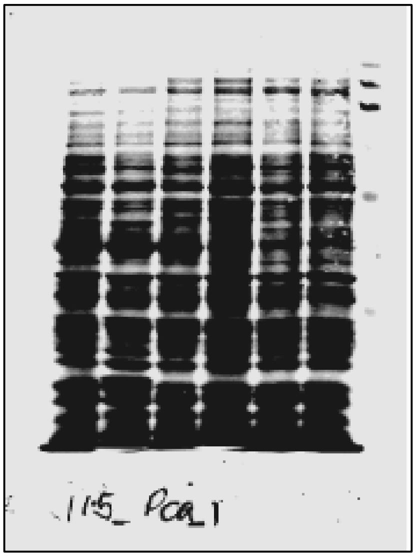

PLEXINB1 200kDa

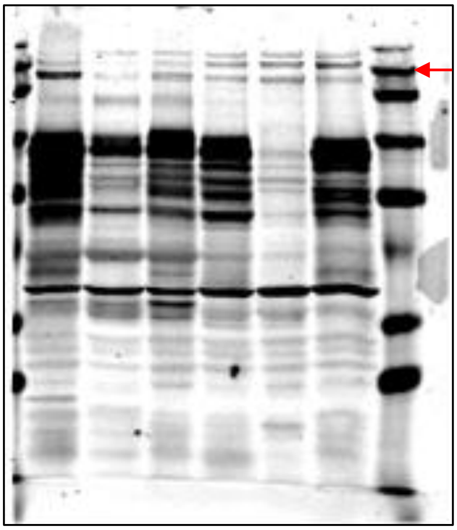

PLEXIND1 212kDa

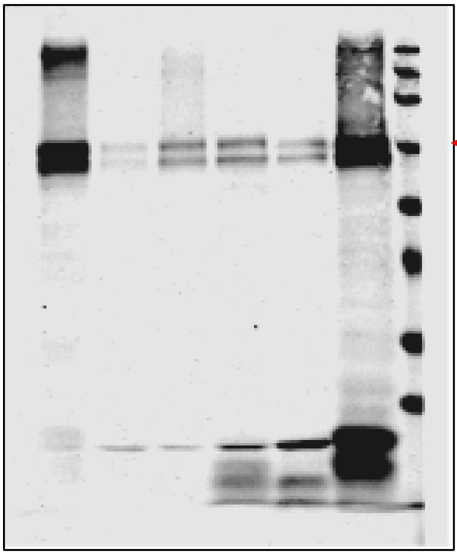

NRP1 120kDa

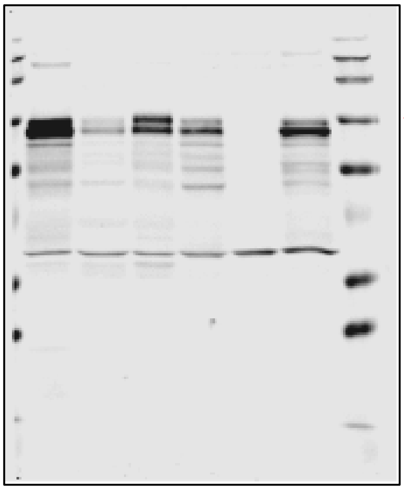

NRP2 120kDa

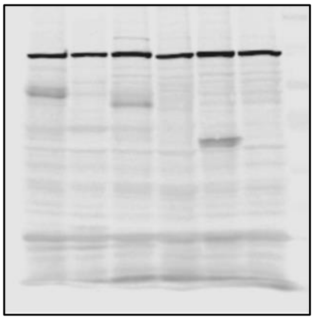

VINCULIN 130kDa

Appendix Figure S2F

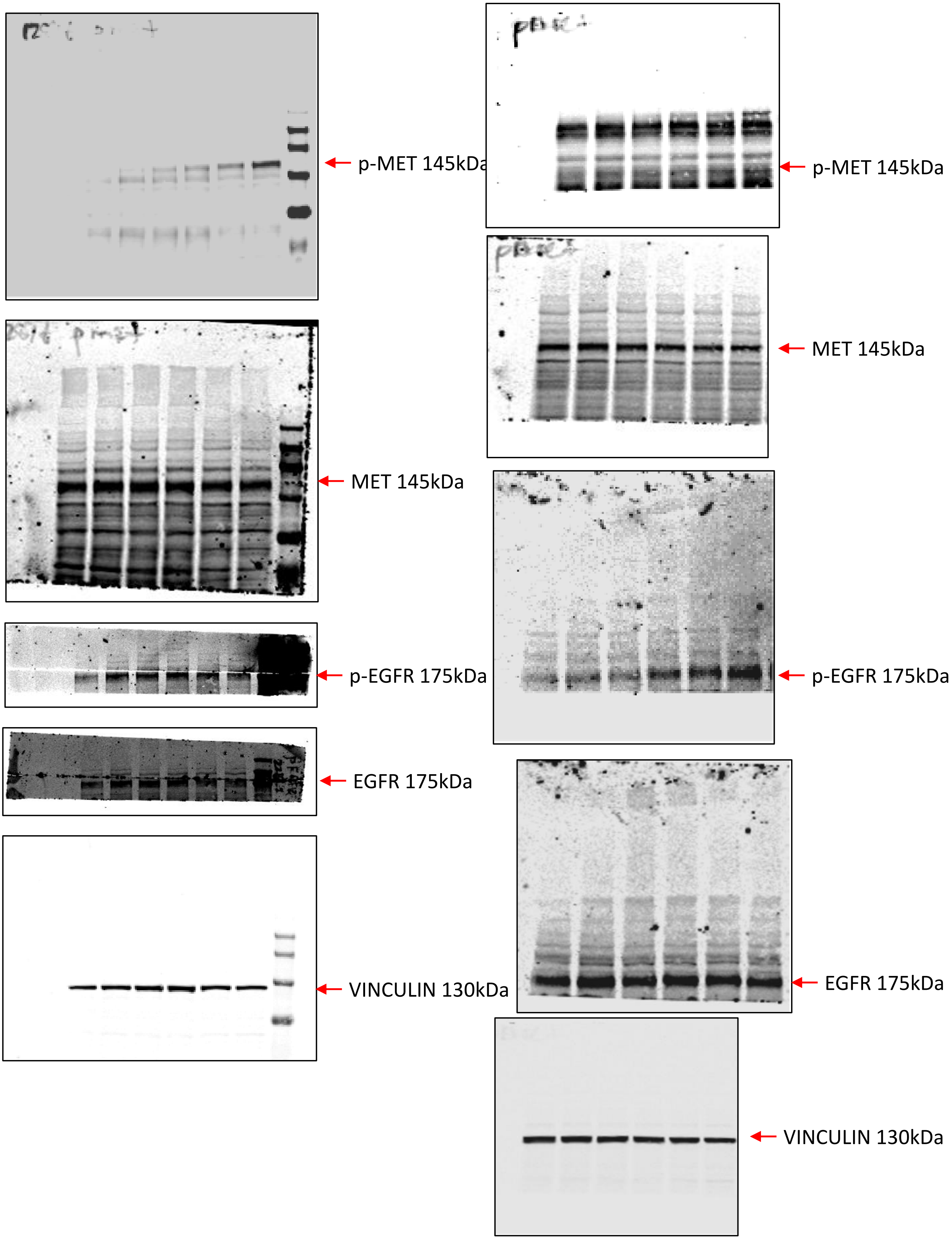

Appendix Figure S2F

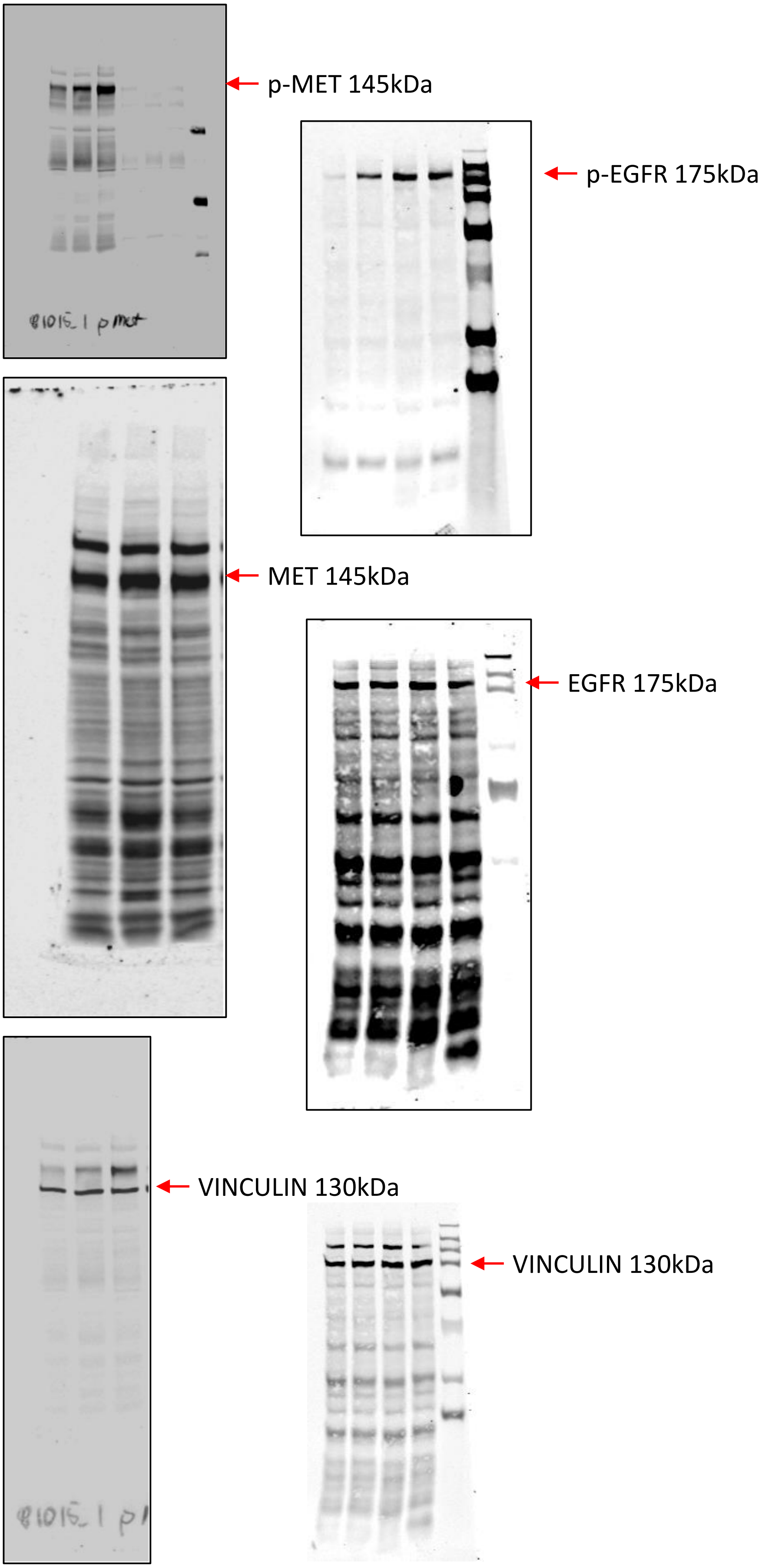

Appendix Figure S2G

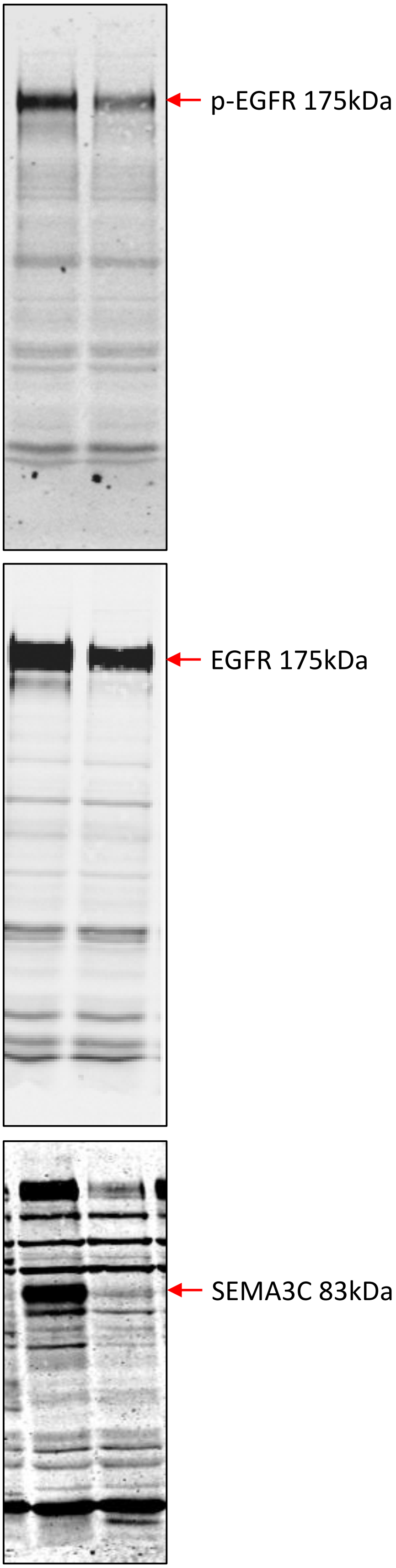

Appendix Figure S2G

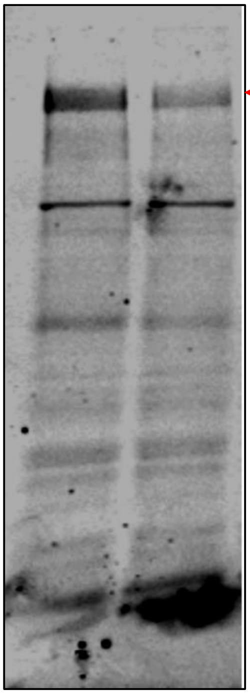

p-EGFR 175kDa

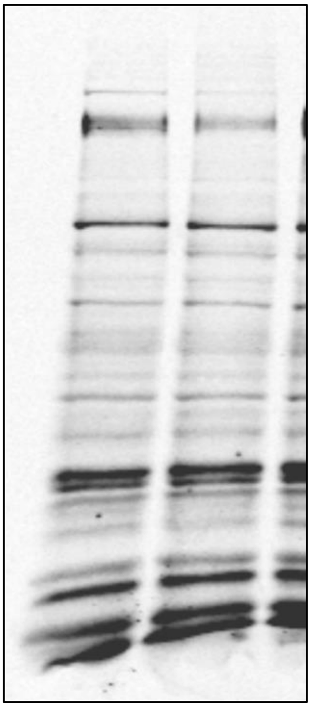

EGFR 175kDa

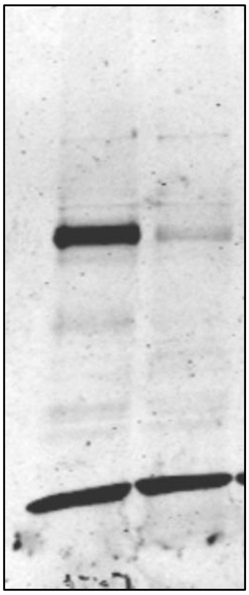

SEMA 3C 83kDa

ACTIN 42kDa

Appendix Figure S2H

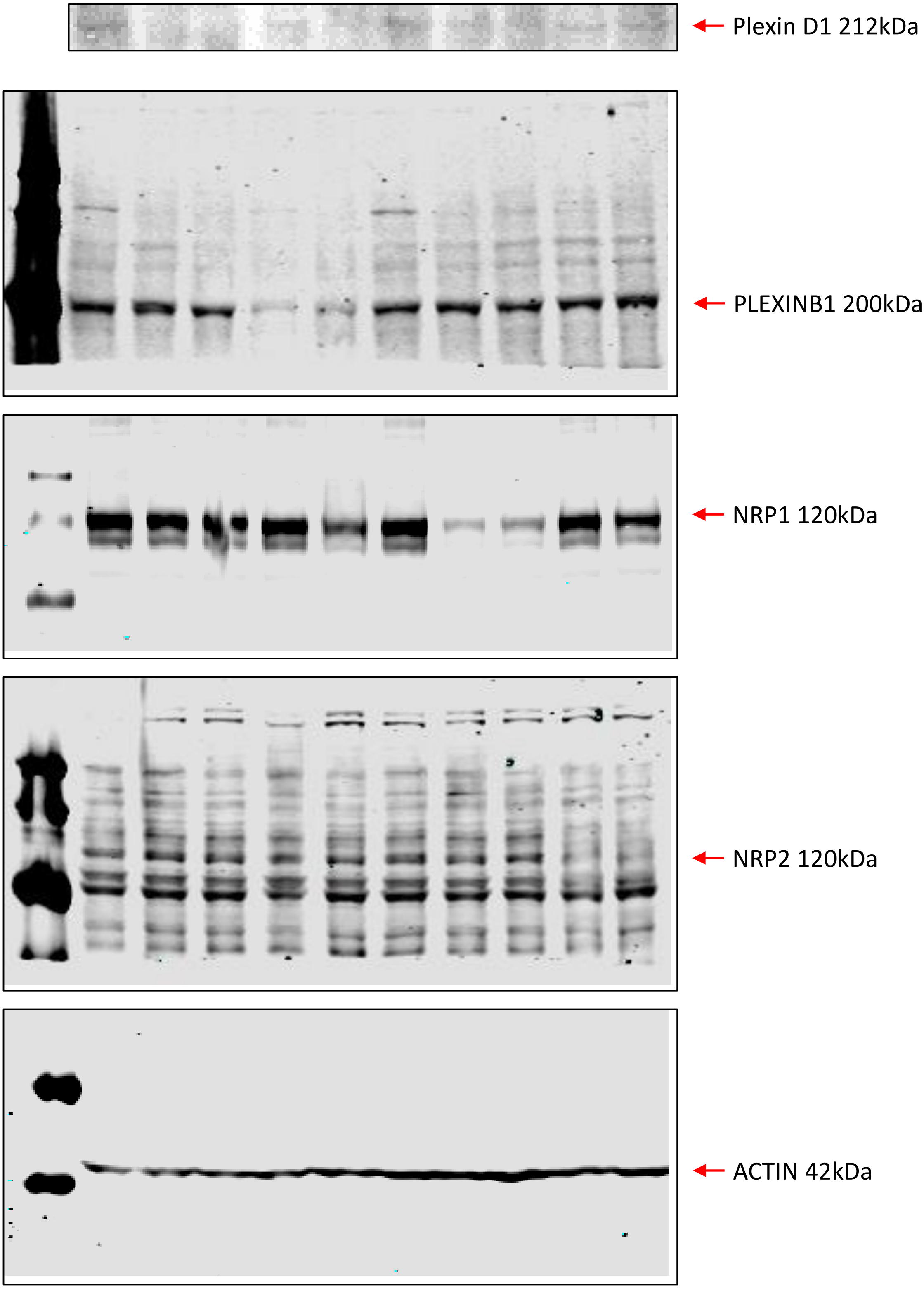

Appendix Figure 2I

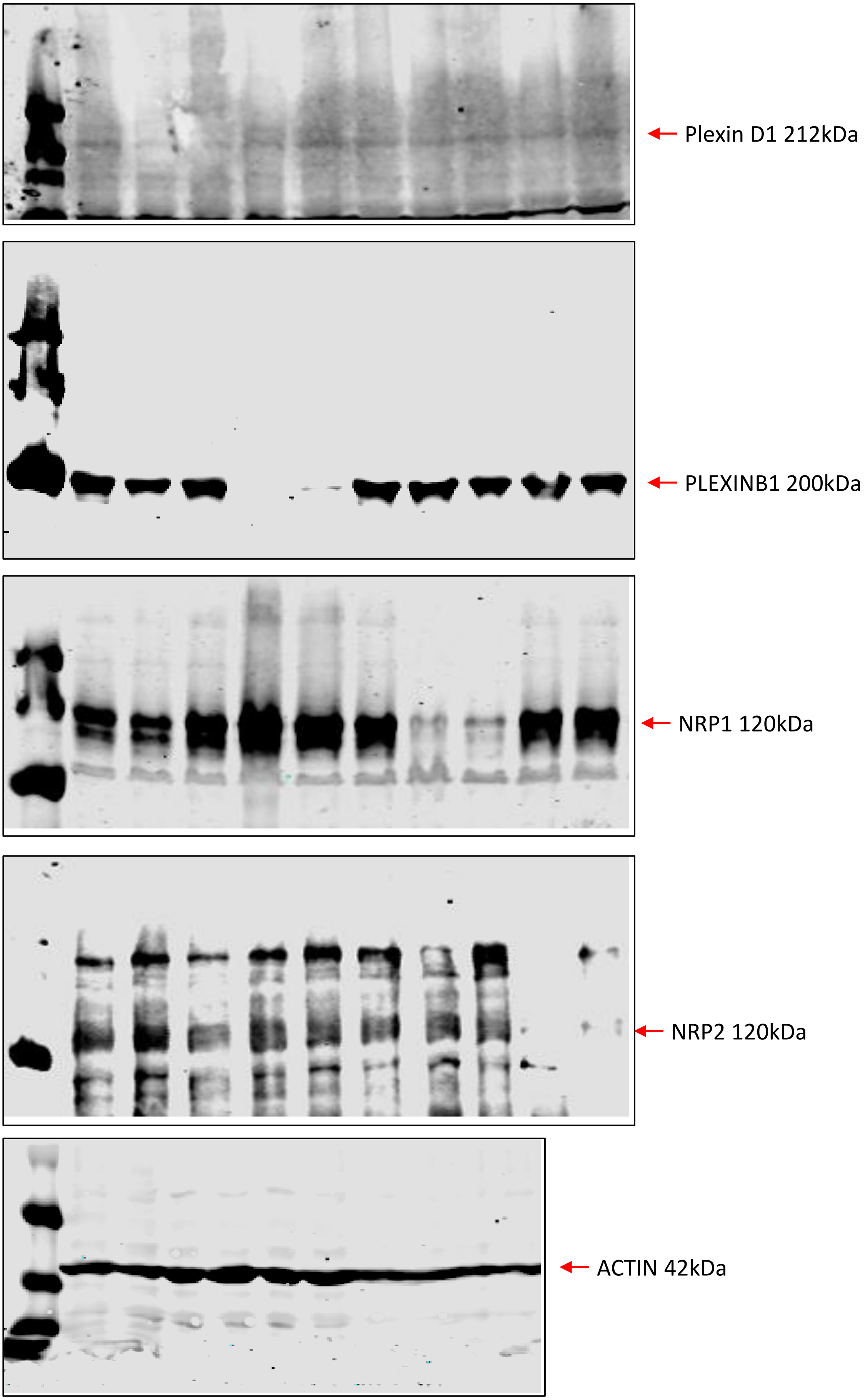

Appendix Figure S2J

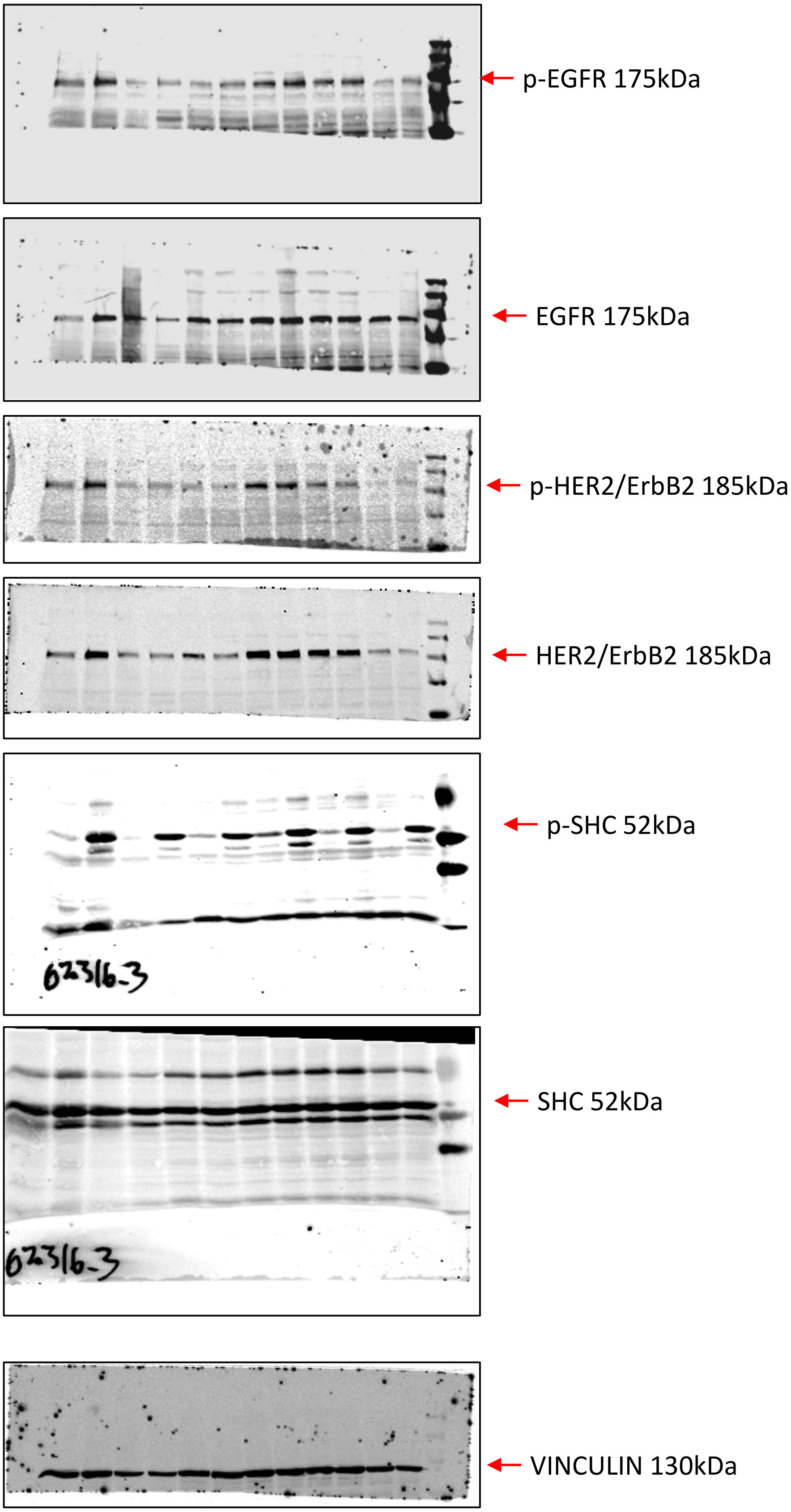

Appendix Figure S2K

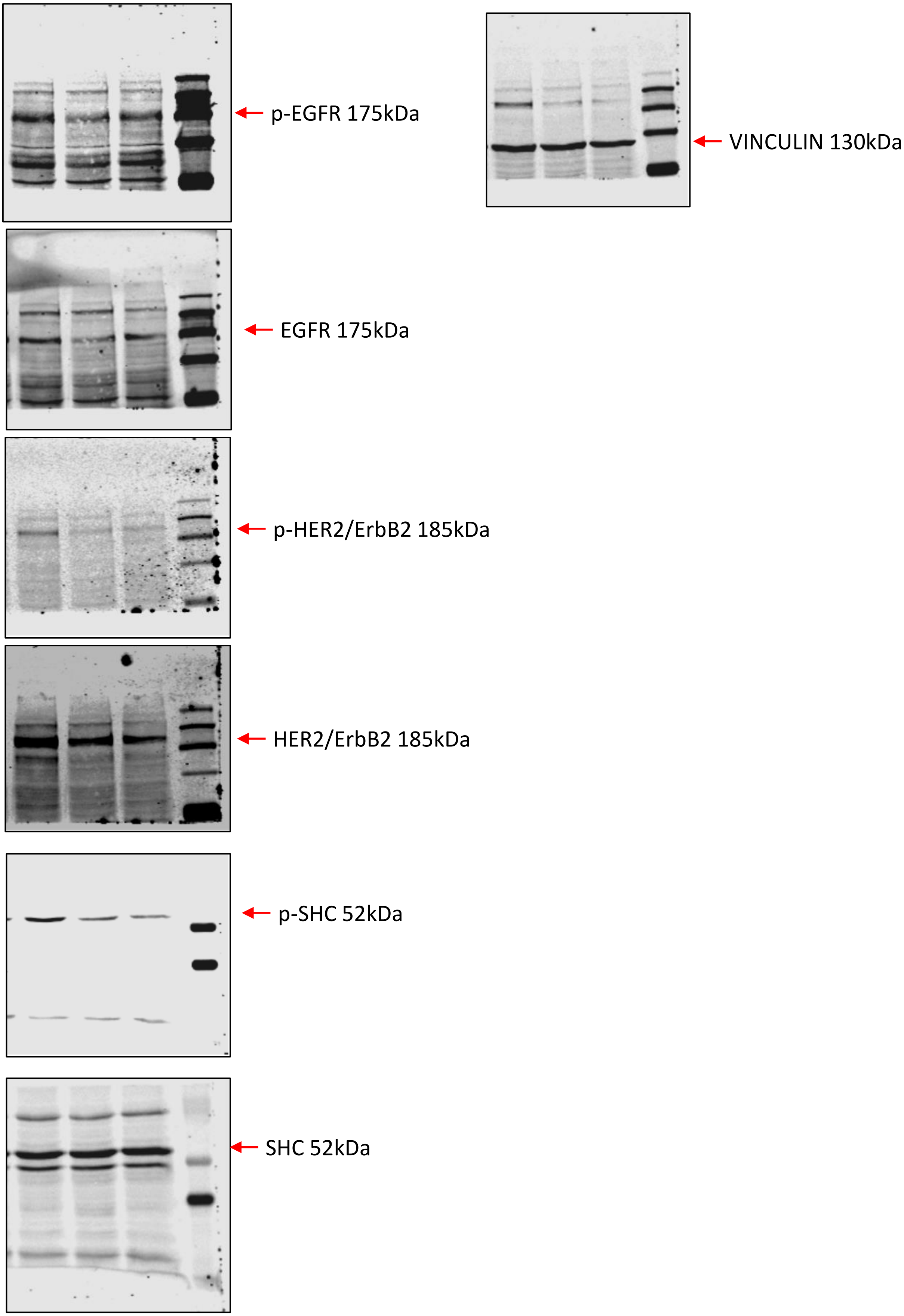

Supplement: Supplementary file 2 — Source Data for Appendix [file EMMM-10-219-s009.zip › Source_data_for_appendix_figures/SD_Appendix_Figure_S2.pdf]
