# Supplementary material for: SEMA3C drives cancer growth by transactivating multiple receptor tyrosine kinases via Plexin B1
Source: EMBO Mol Med. 2018 Jan 18;10(2):219–38. doi: 10.15252/emmm.201707689 (PMC5801490; doi:10.15252/emmm.201707689)
Supplement: Supplementary file 2 — Source Data for Appendix [file EMMM-10-219-s009.zip › Source_data_for_appendix_figures/SD_Appendix_Figure_S3.pdf]

Appendix Figure S3 A

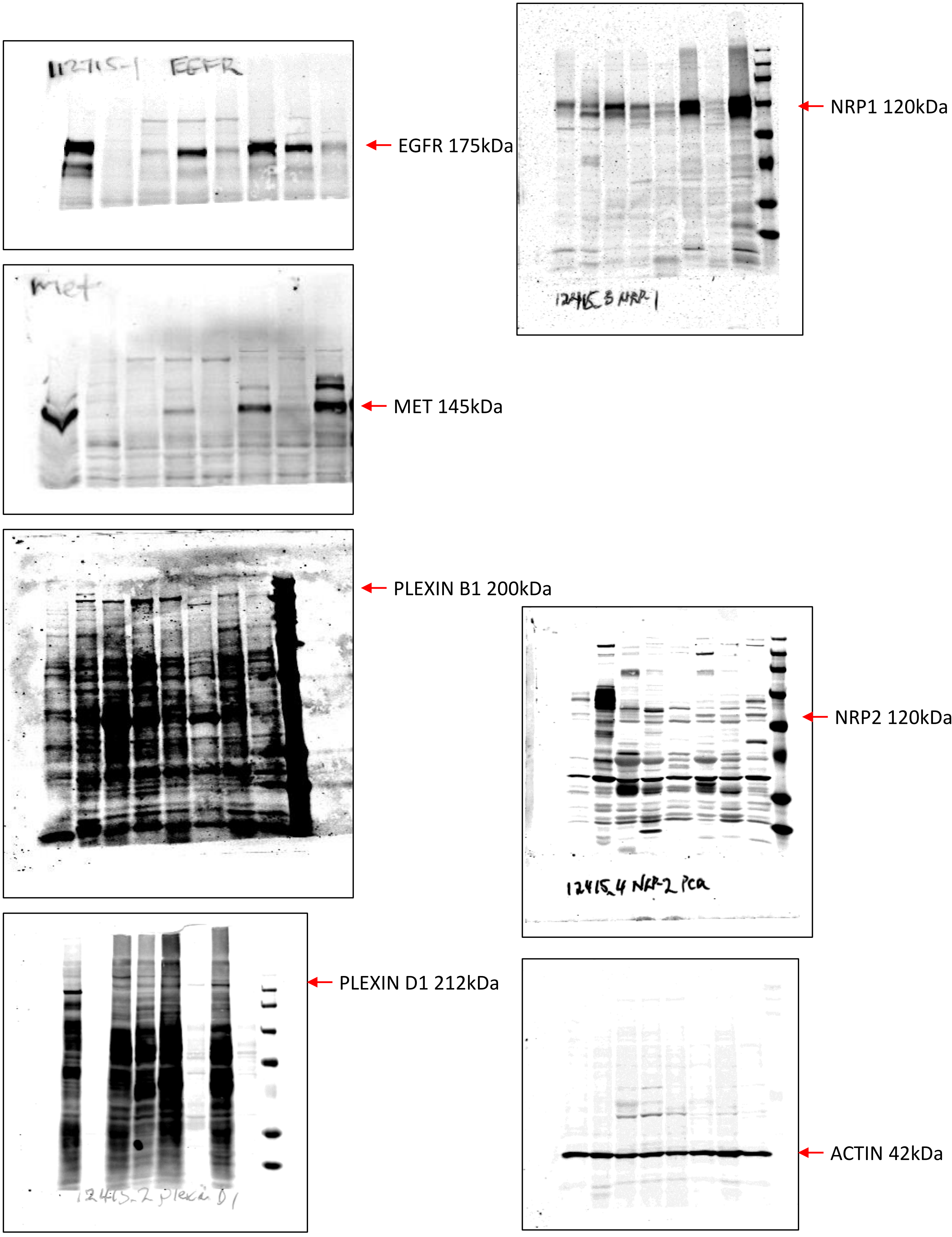

Appendix Figure S3 C

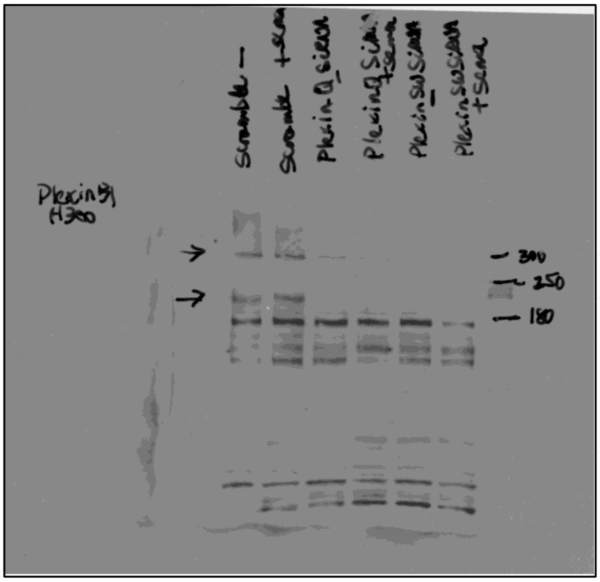

PLEXIN B1 300kDa

PLEXIN B1 200kDa

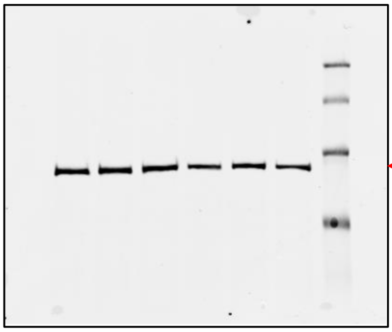

VINCULIN 130kDa

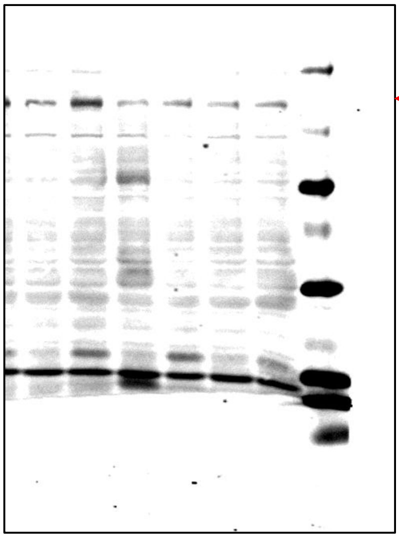

p-EGFR 175kDa

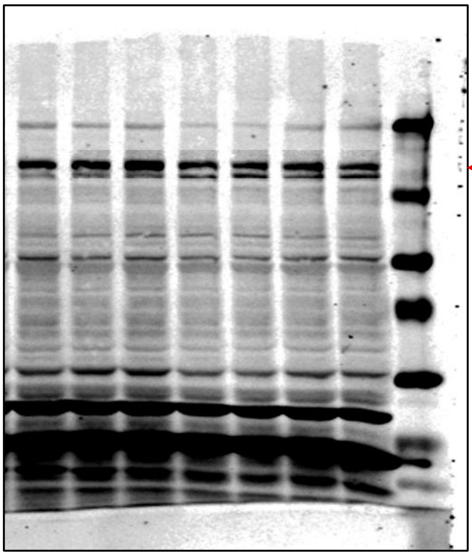

EGFR 175kDa

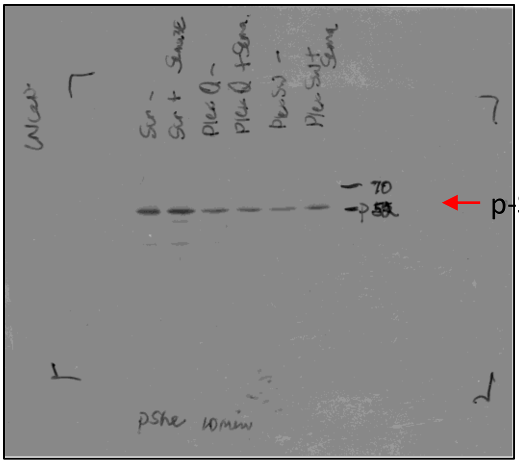

p-SHC 52kDa

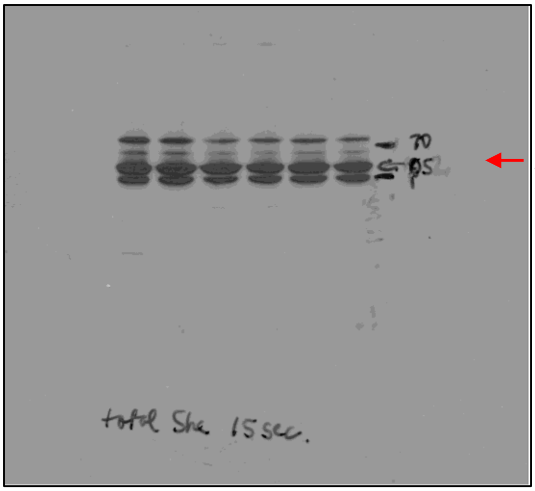

SHC 52kDa

Apppendix Figure S3E

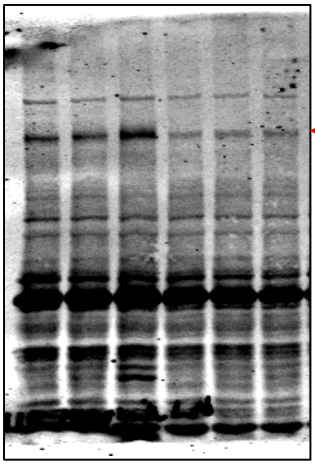

p-EGFR 175kDa

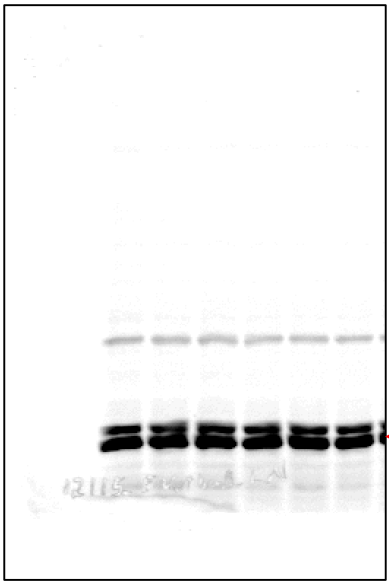

p42/44 MAPK

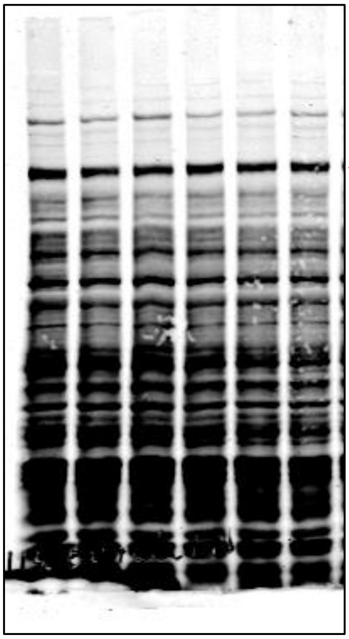

EGFR 175kDa

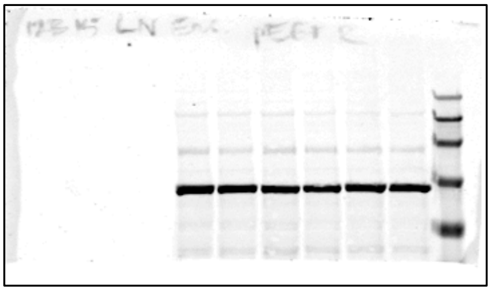

VINCULIN 130kDa

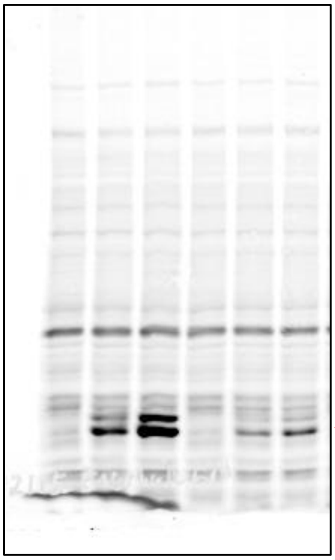

p42/44 p-MAPK
