# Supplementary material for: SEMA3C drives cancer growth by transactivating multiple receptor tyrosine kinases via Plexin B1
Source: EMBO Mol Med. 2018 Jan 18;10(2):219–38. doi: 10.15252/emmm.201707689 (PMC5801490; doi:10.15252/emmm.201707689)

Appendix Figure S5E

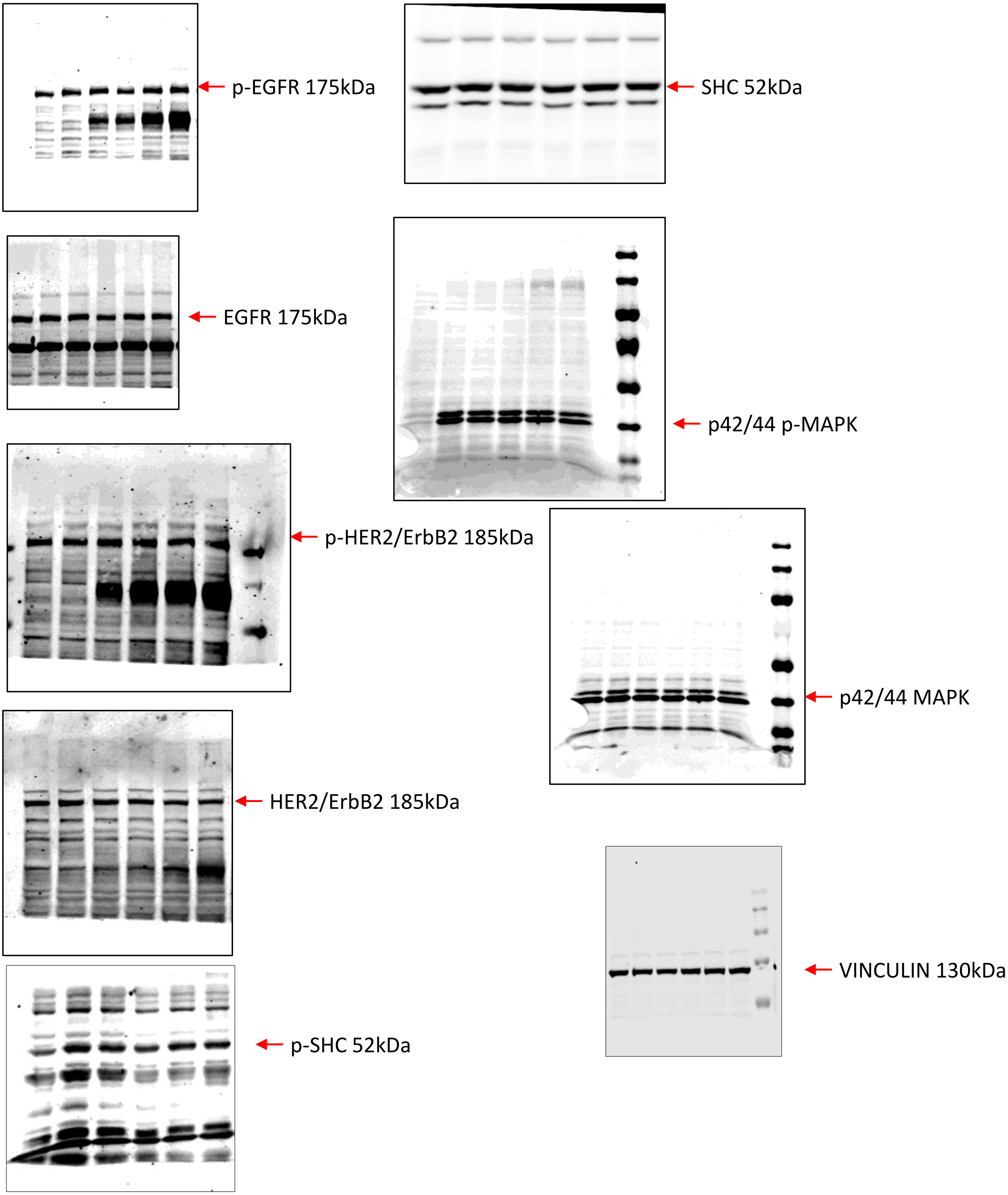

Appendix Figure S5F

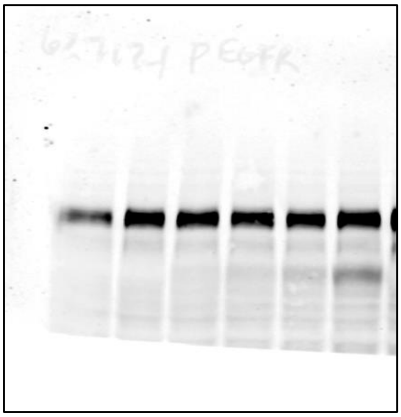

p-EGFR 175kDa

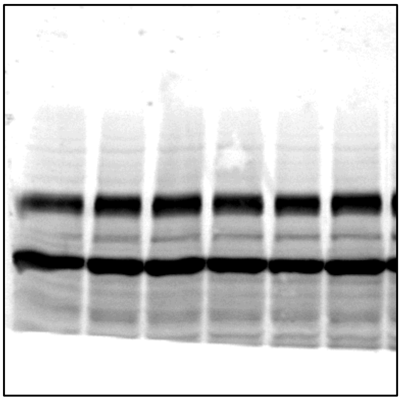

EGFR 175kDa

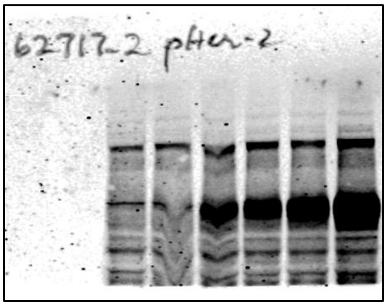

p-HER2/ErbB2 185kDa

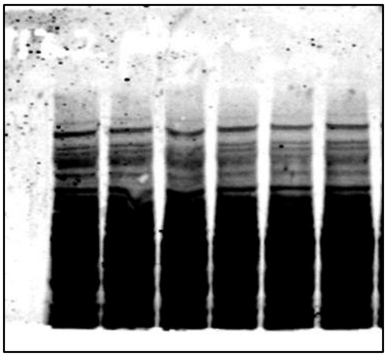

HER2/ErbB2 185kDa

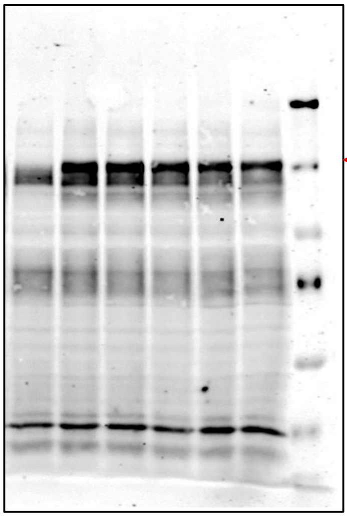

p-MET 145kDa

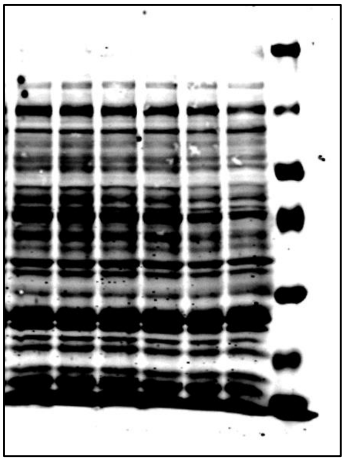

MET 145kDa

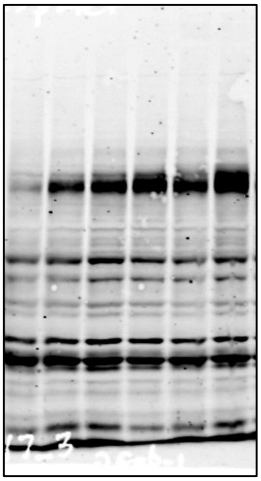

p-GAB-110kDa

Appendix Figure S5F

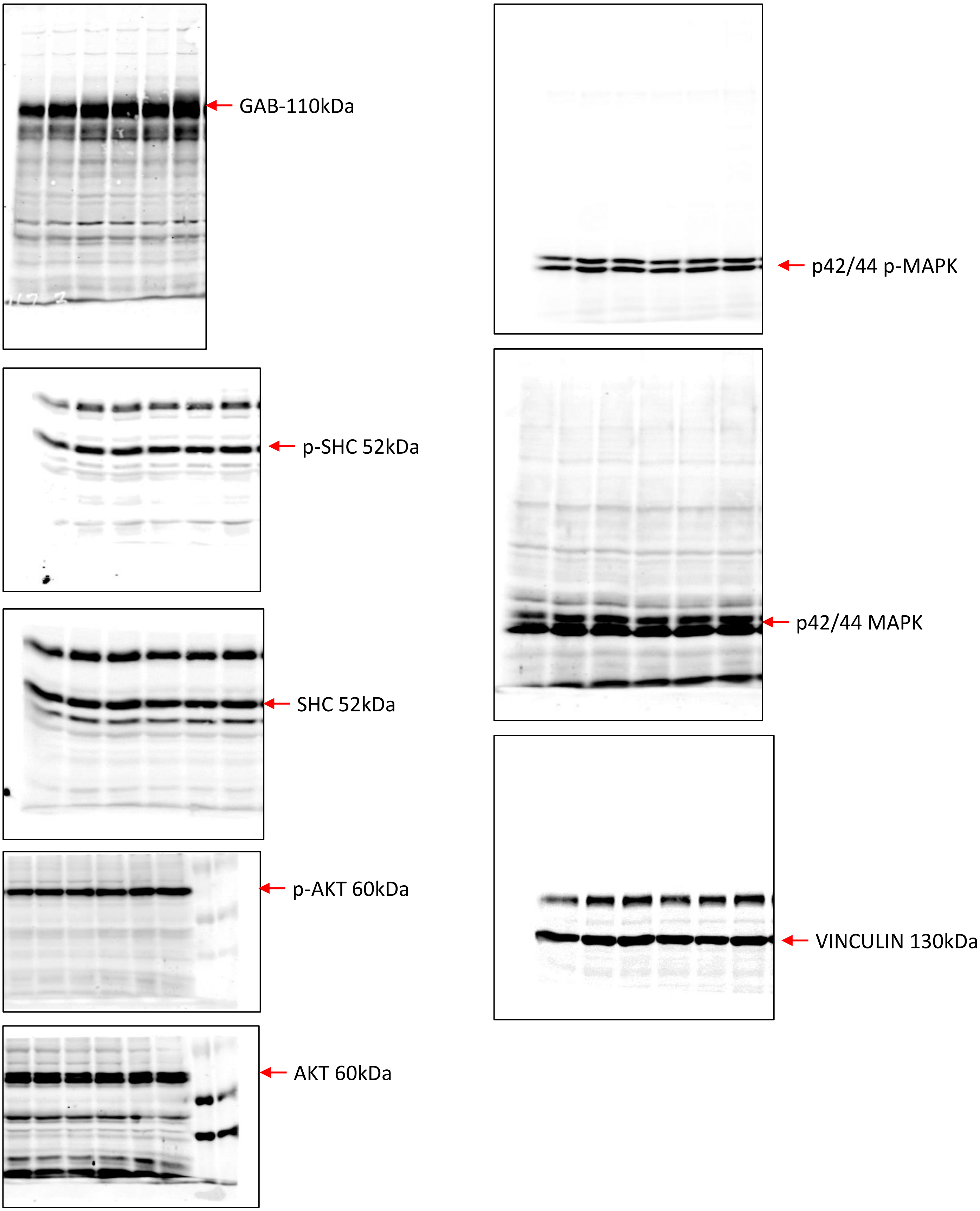

Appendix Figure S5G

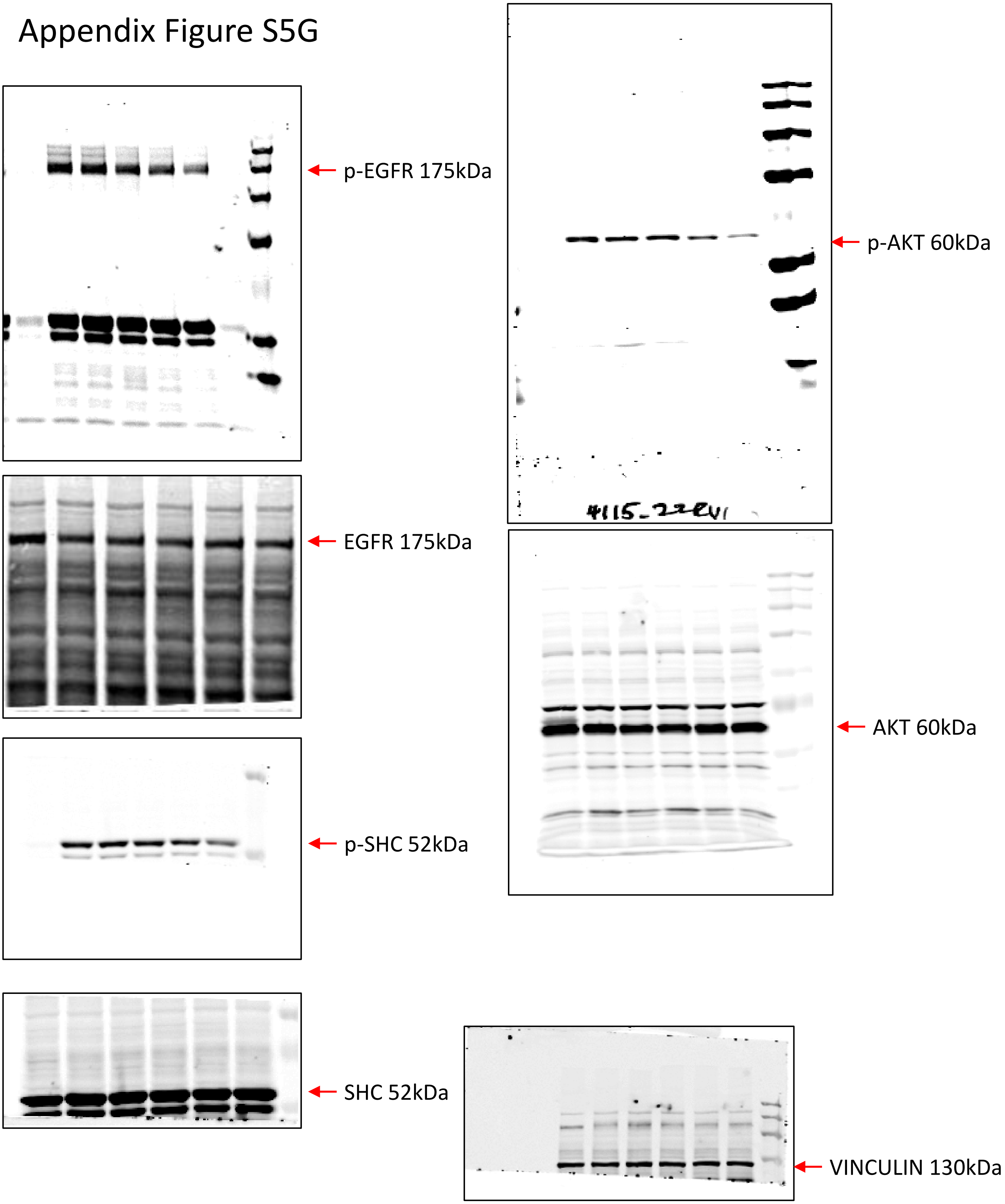

Appendix Figure S5H

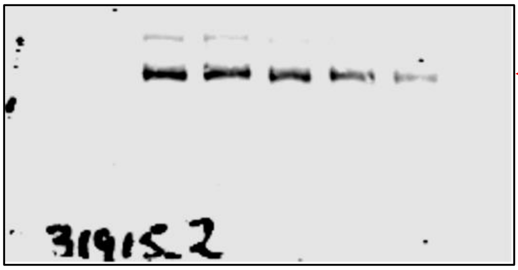

p-EGFR 175kDa

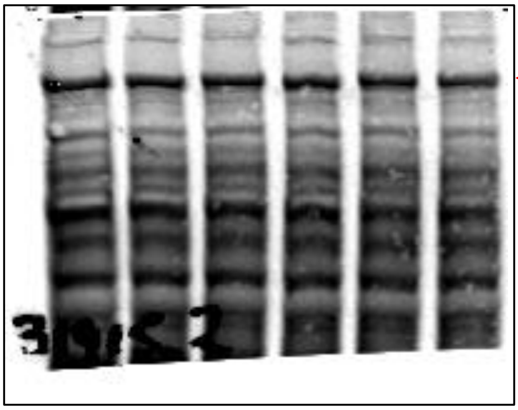

EGFR 175kDa

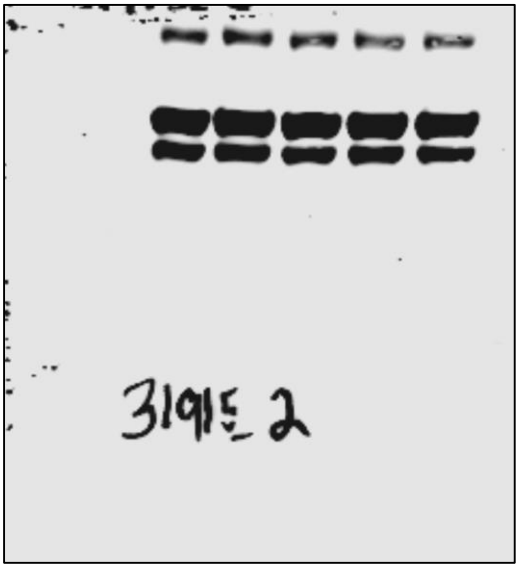

p-SHC 66kDa

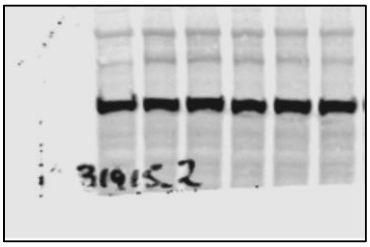

VINCULIN 130kDa

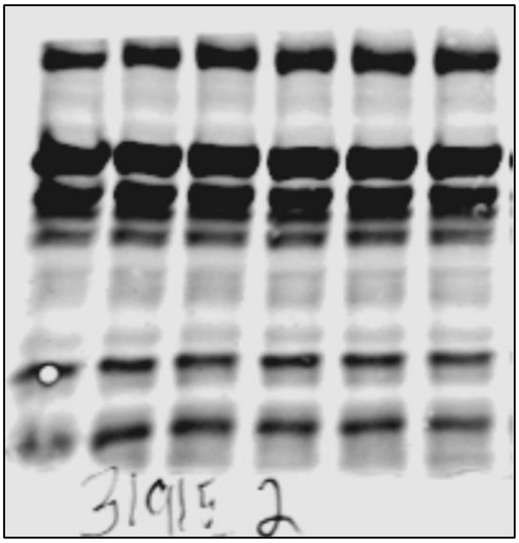

SHC 66kDa

Appendix Figure S5I

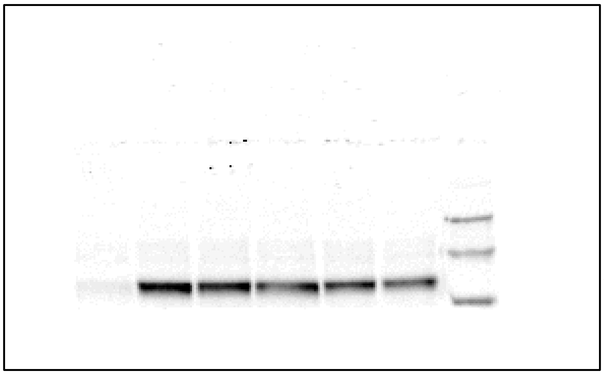

p-MET 145kDa

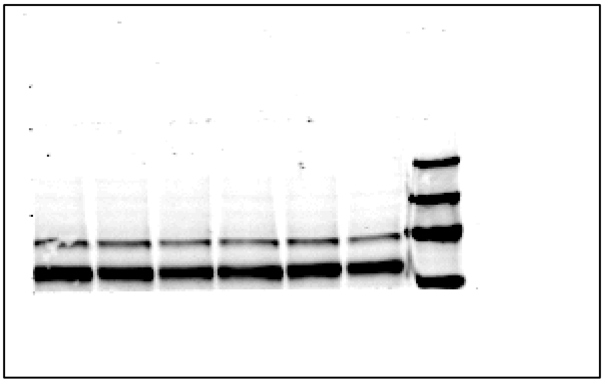

MET 145kDa

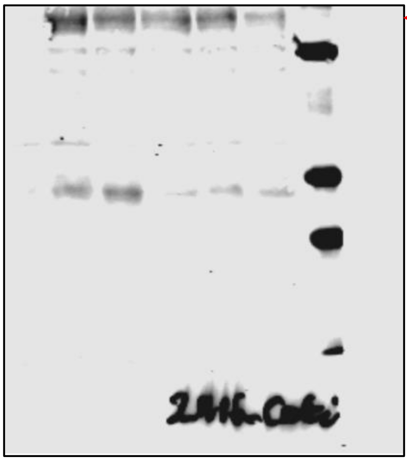

p-GAB-1 110kDa

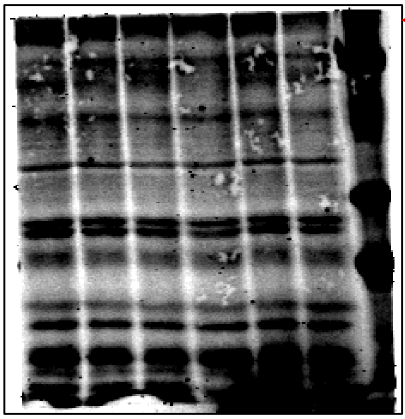

GAB-1 110kDa

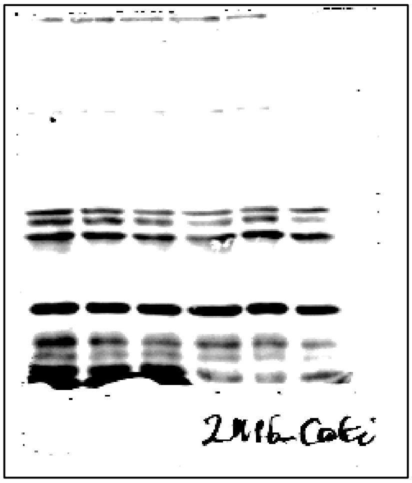

ACTIN 42kDa

Appendix Figure S5J

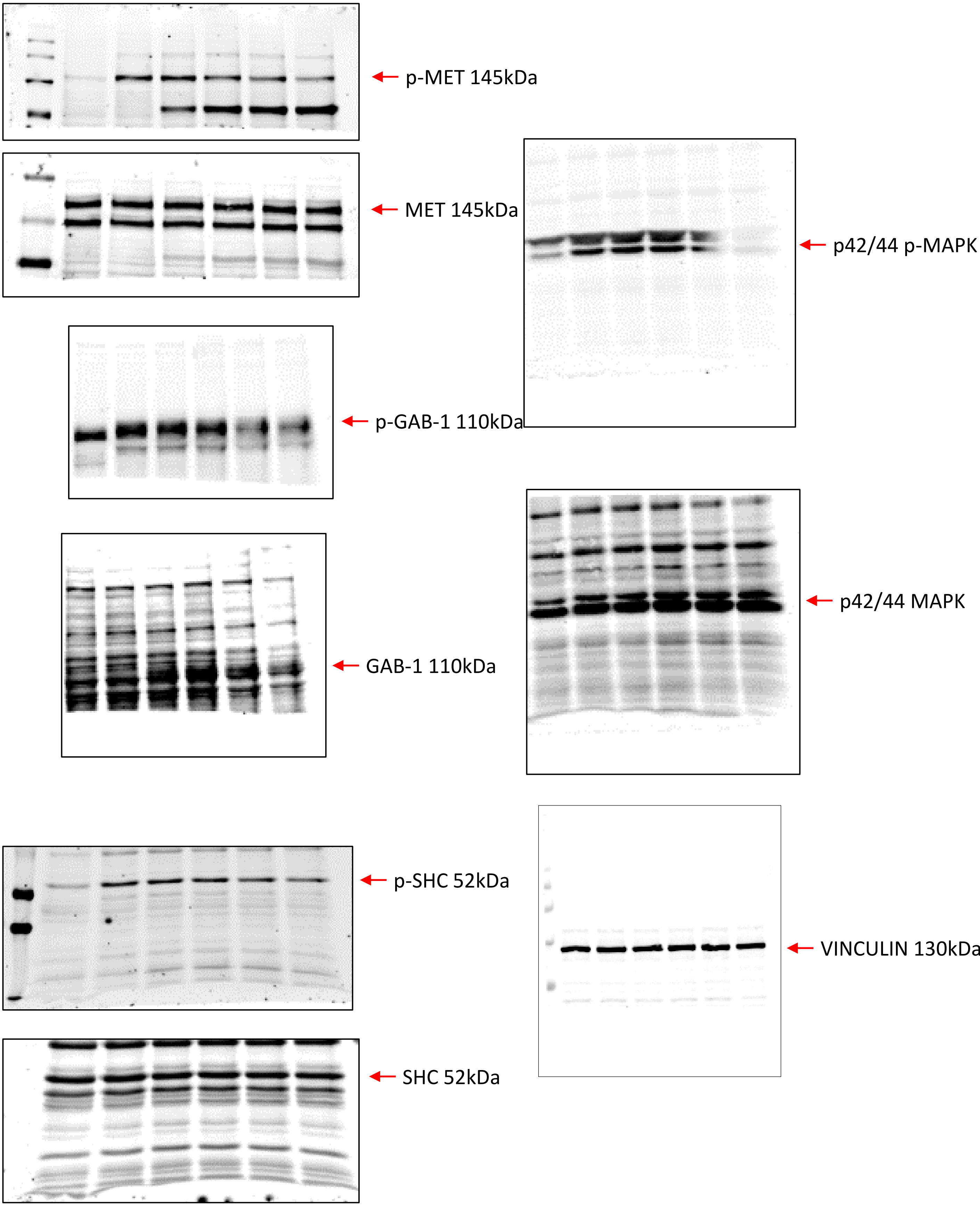

Appendix Figure S5K

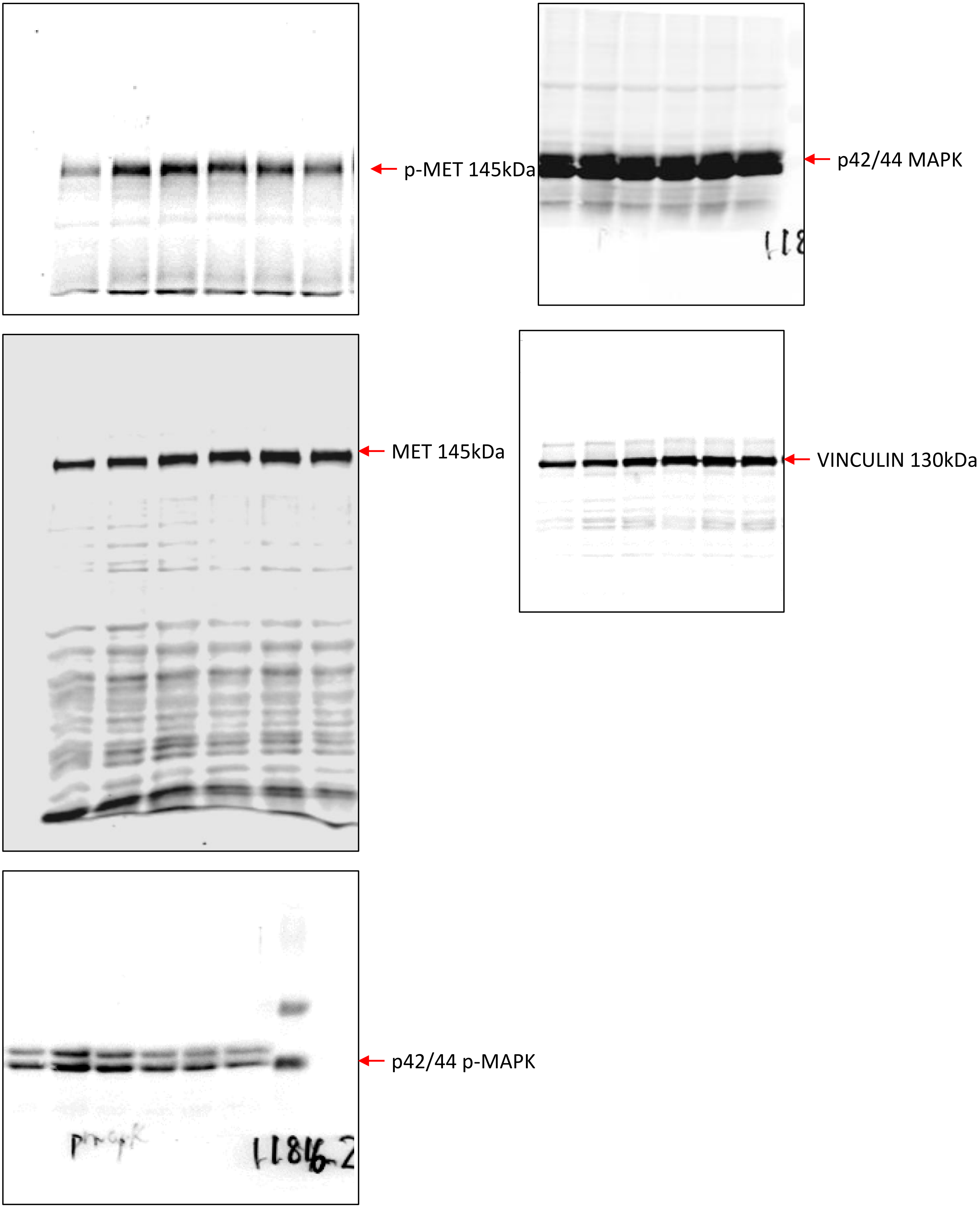

Supplement: Supplementary file 2 — Source Data for Appendix [file EMMM-10-219-s009.zip › Source_data_for_appendix_figures/SD_Appendix_Figure_S5.pdf]
