# Supplementary figures and images for: SEMA3C drives cancer growth by transactivating multiple receptor tyrosine kinases via Plexin B1
Source: EMBO Mol Med. 2018 Jan 18;10(2):219–38. doi: 10.15252/emmm.201707689 (PMC5801490; doi:10.15252/emmm.201707689)

Appendix Figure S1B

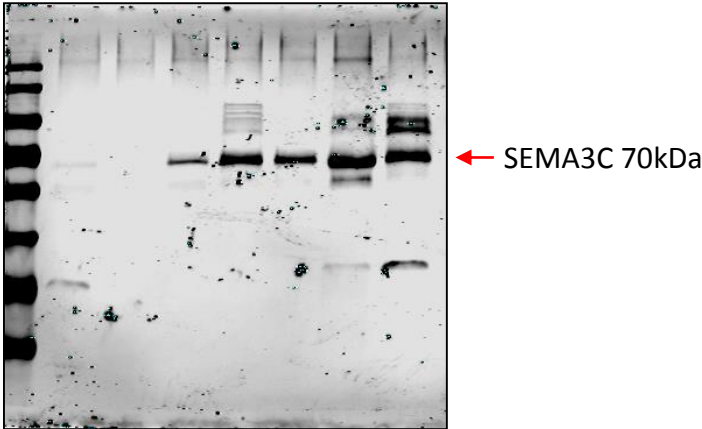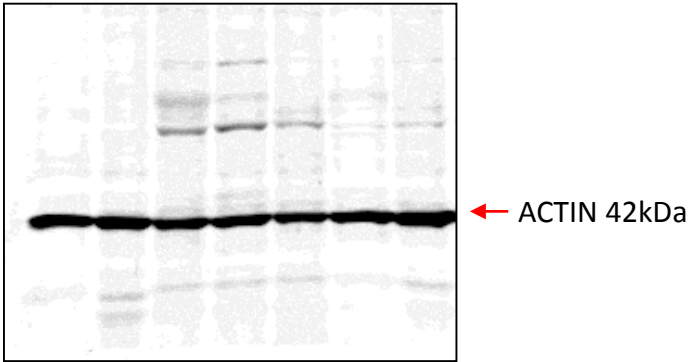

Appendix Figure S1E

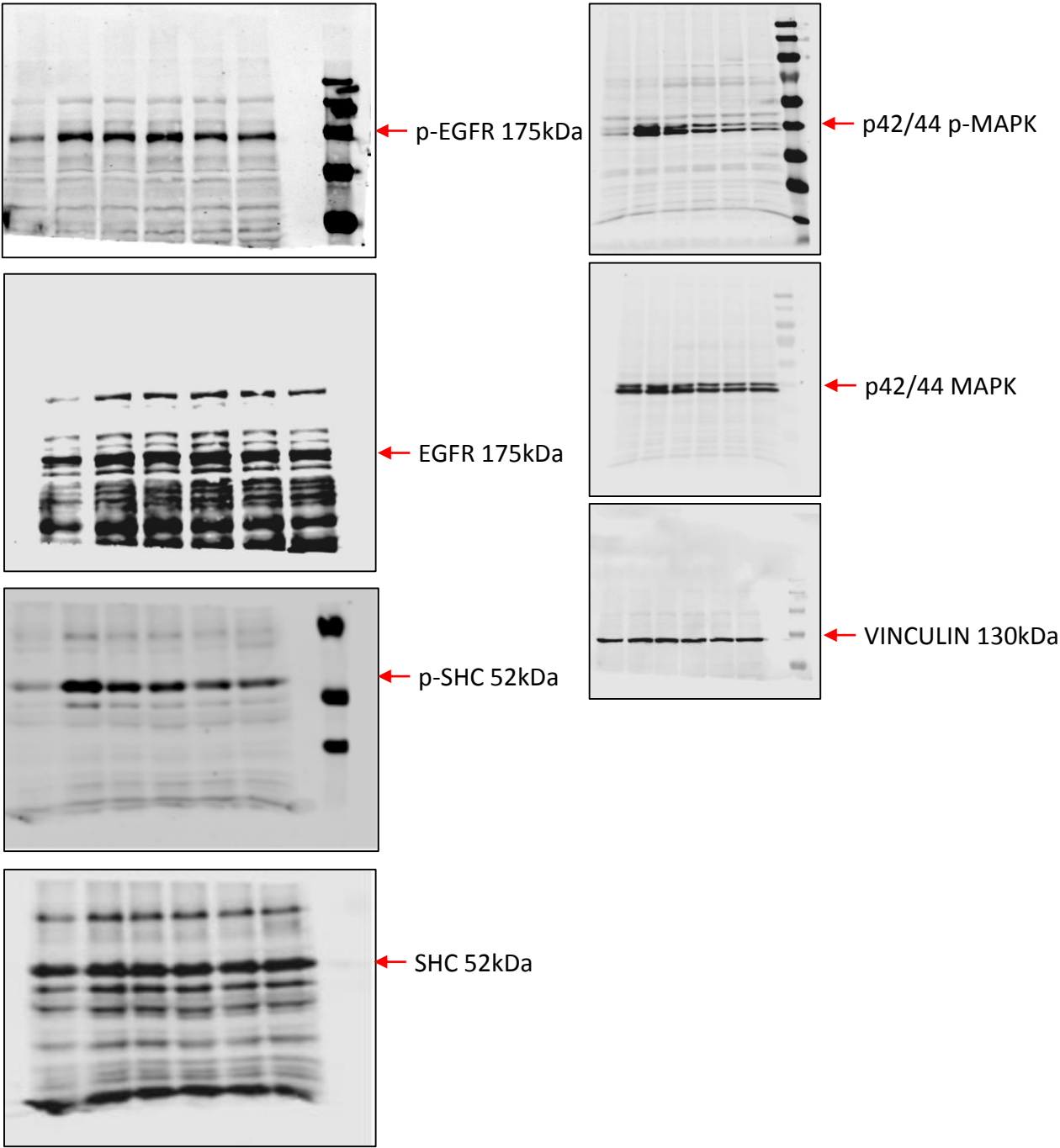

Appendix Figure S1E

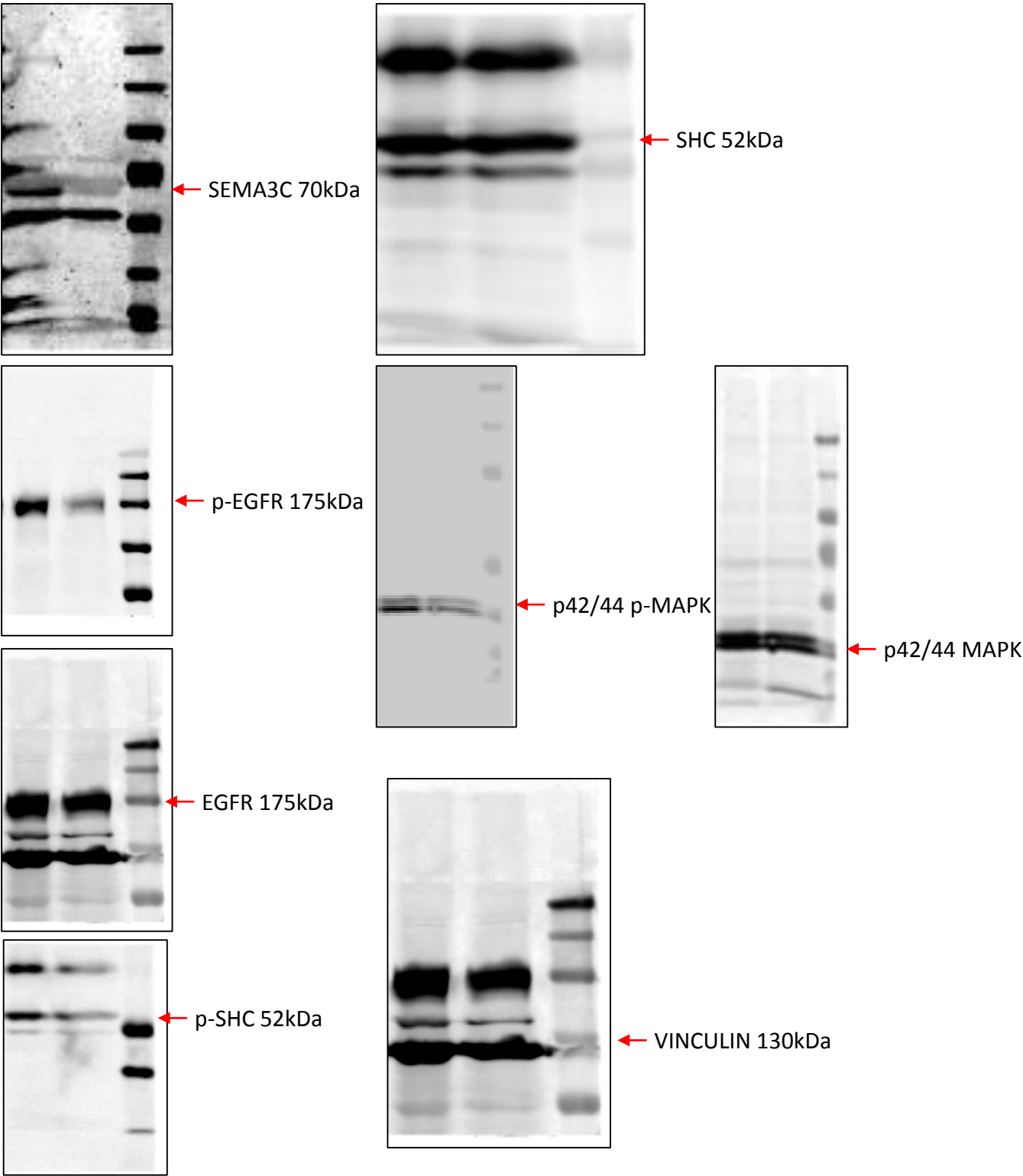

Appendix Figure S1F

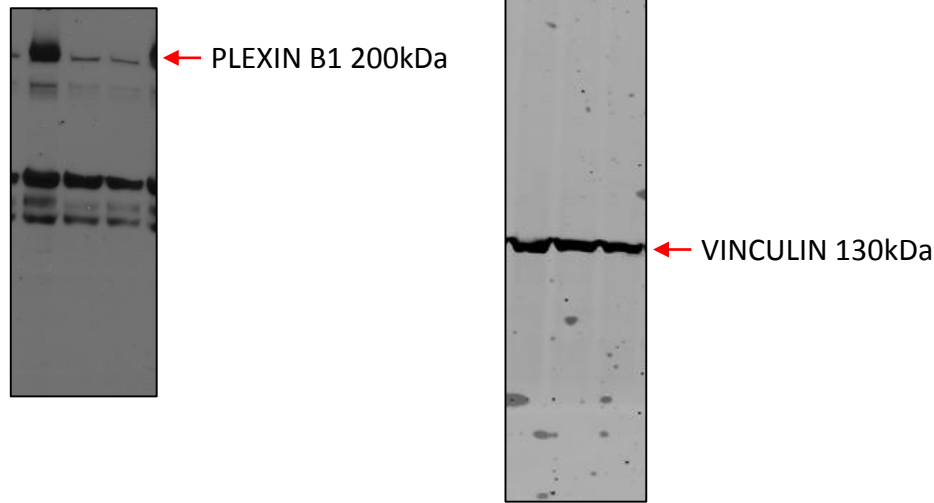

Supplement: Supplementary file 2 — Source Data for Appendix [file EMMM-10-219-s009.zip › Source_data_for_appendix_figures/SD_Appendix_Figure_S1.pdf]

Appendix Figure S4

Experiment repeated to provide source data

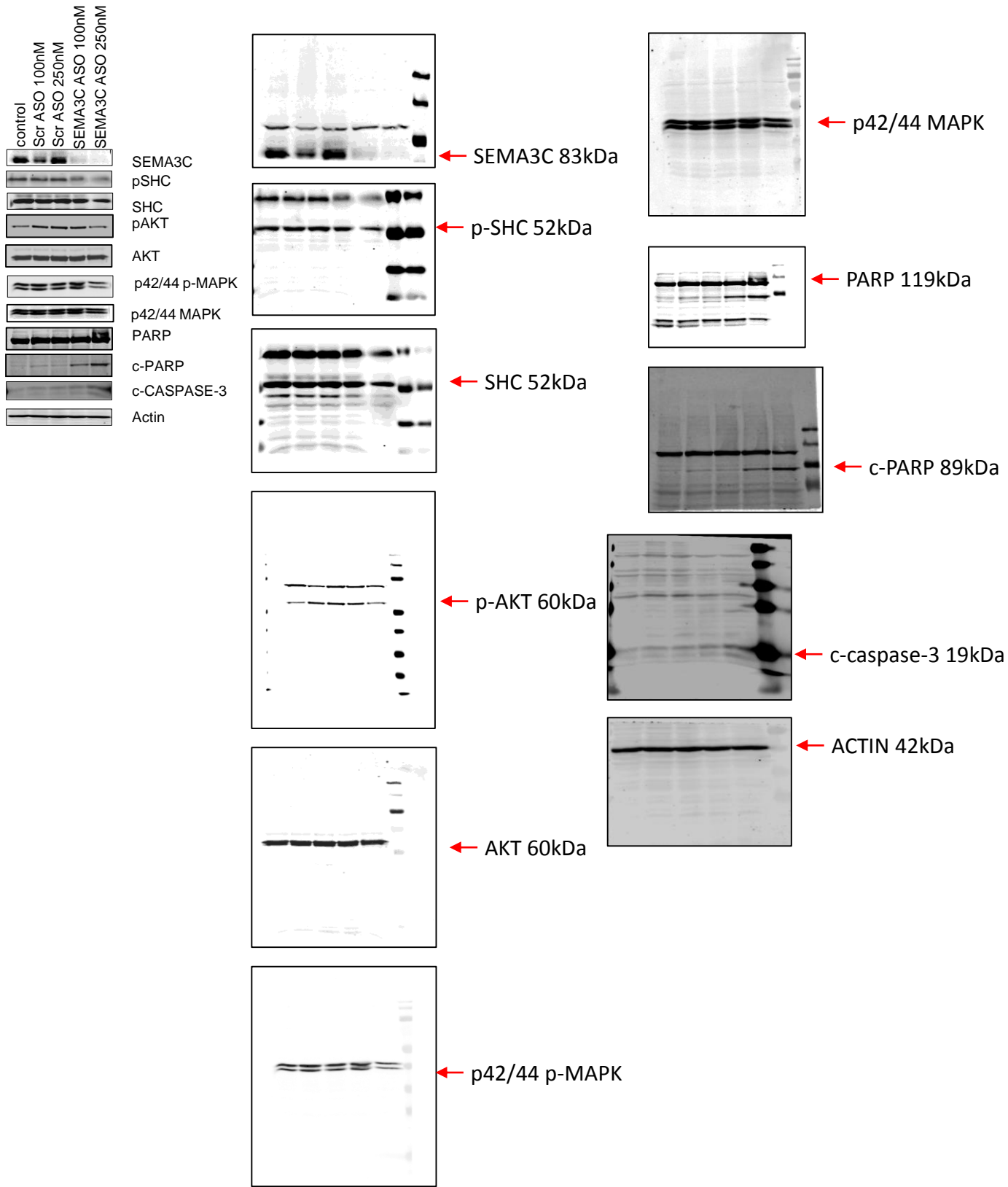

Supplement: Supplementary file 2 — Source Data for Appendix [file EMMM-10-219-s009.zip › Source_data_for_appendix_figures/SD_Appendix_Figure_S4.pdf]

Appendix Figure S6B

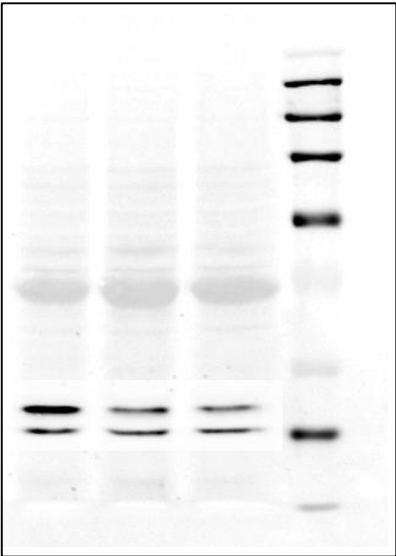

← p42/44 p-MAPK

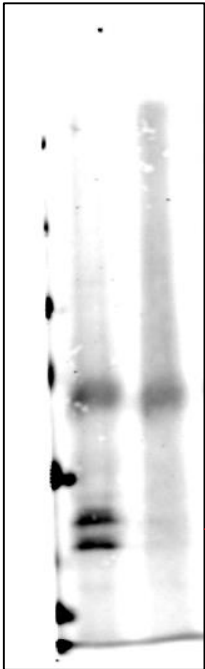

← p42/44 p-MAPK

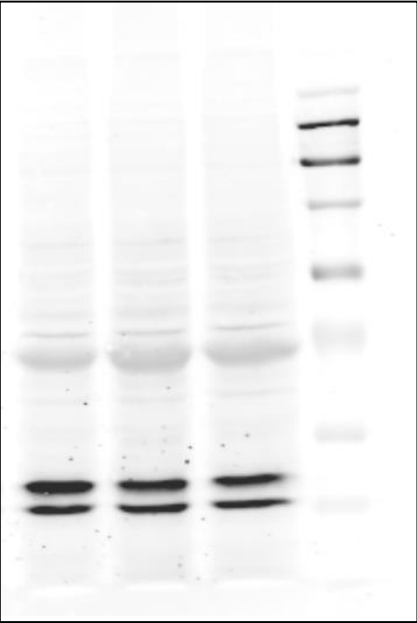

← p42/44 MAPK

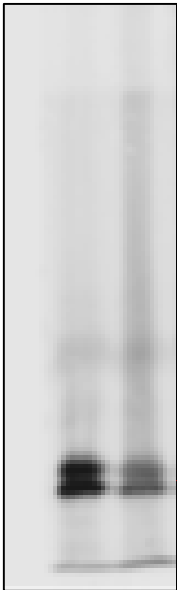

← p42/44 MAPK

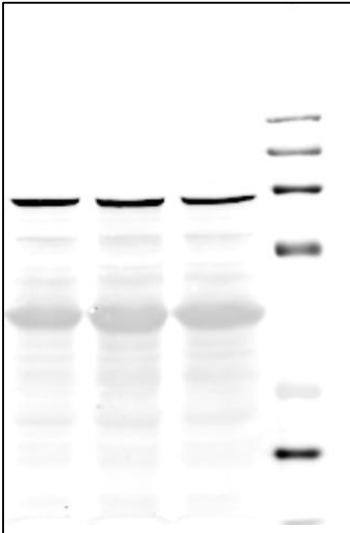

← VINCULIN 130kDa

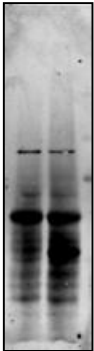

← VINCULIN 130kDa

Supplement: Supplementary file 2 — Source Data for Appendix [file EMMM-10-219-s009.zip › Source_data_for_appendix_figures/SD_Appendix_Figure_S6.pdf]

Figure 1C

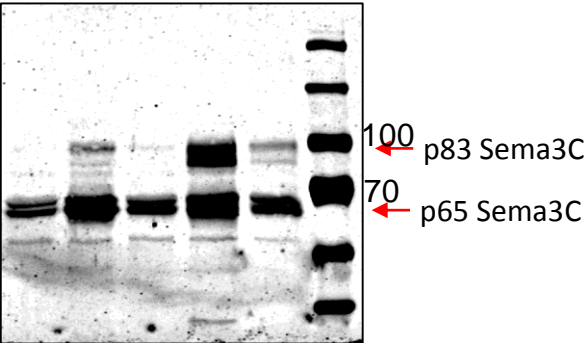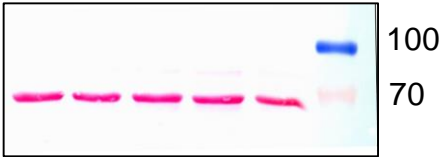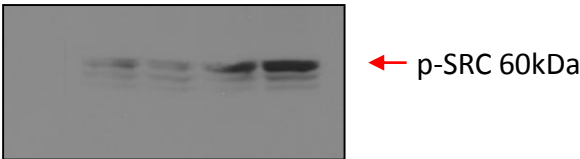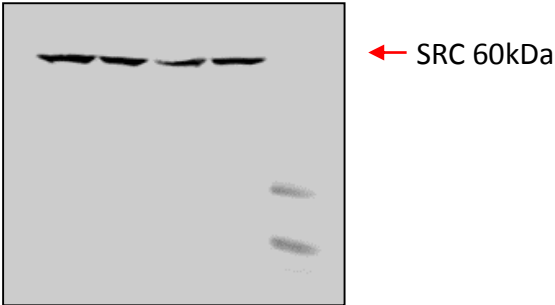

Figure 1D

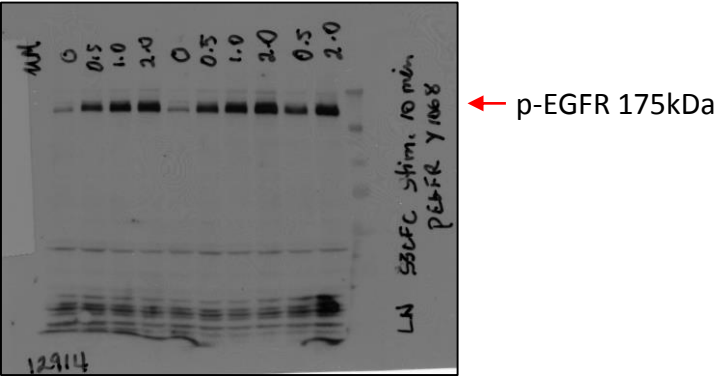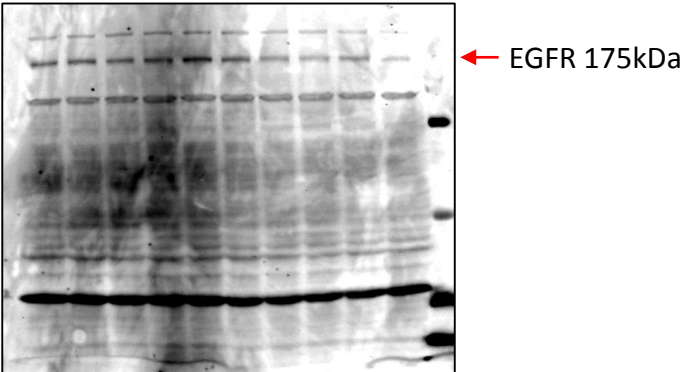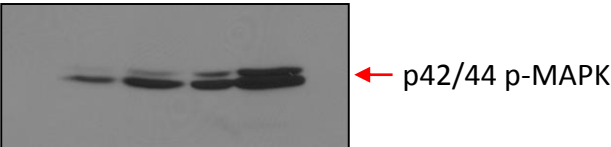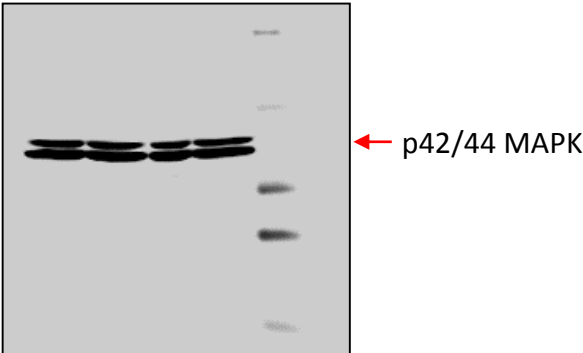

Figure1 D

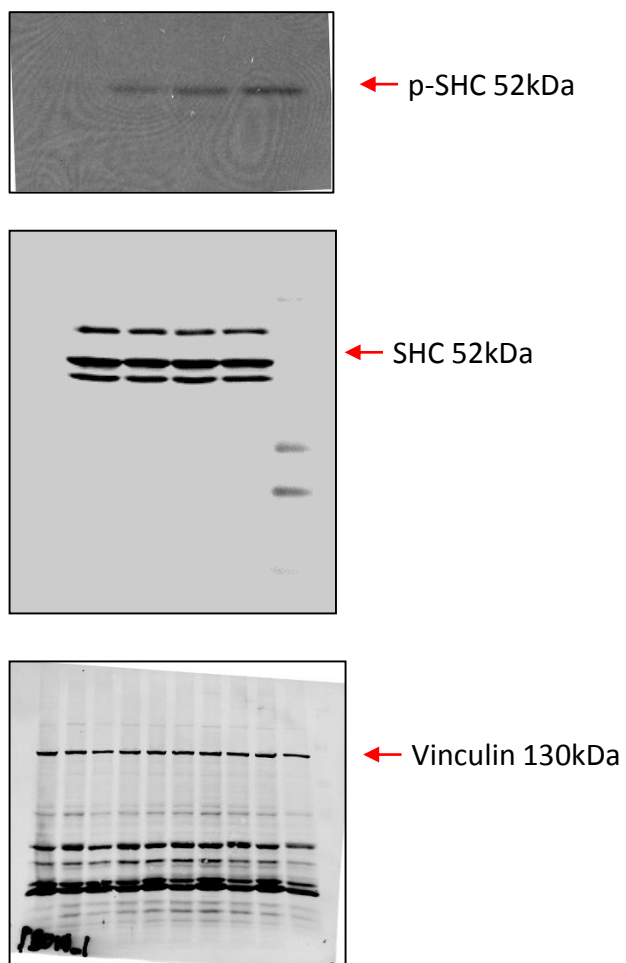

Figure1 F

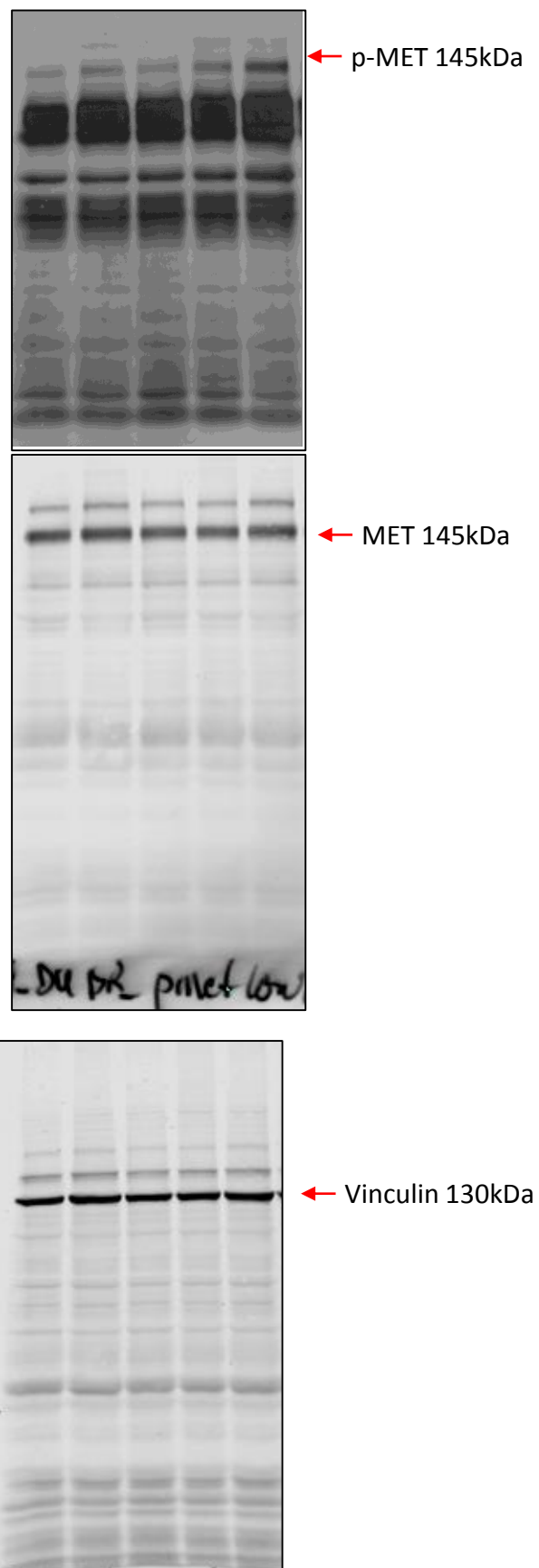

Supplement: Supplementary file 4 — Source Data for Figure 1 [file EMMM-10-219-s002.pdf]

Figure 2 E

IP

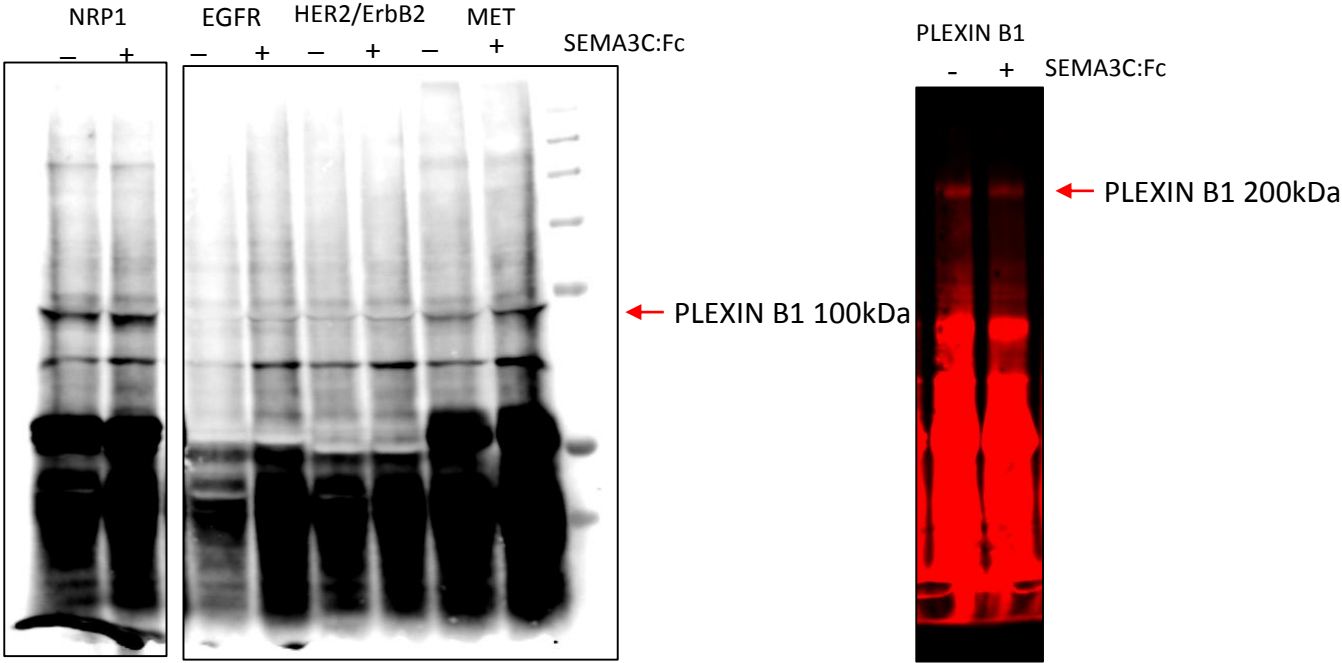

inputs

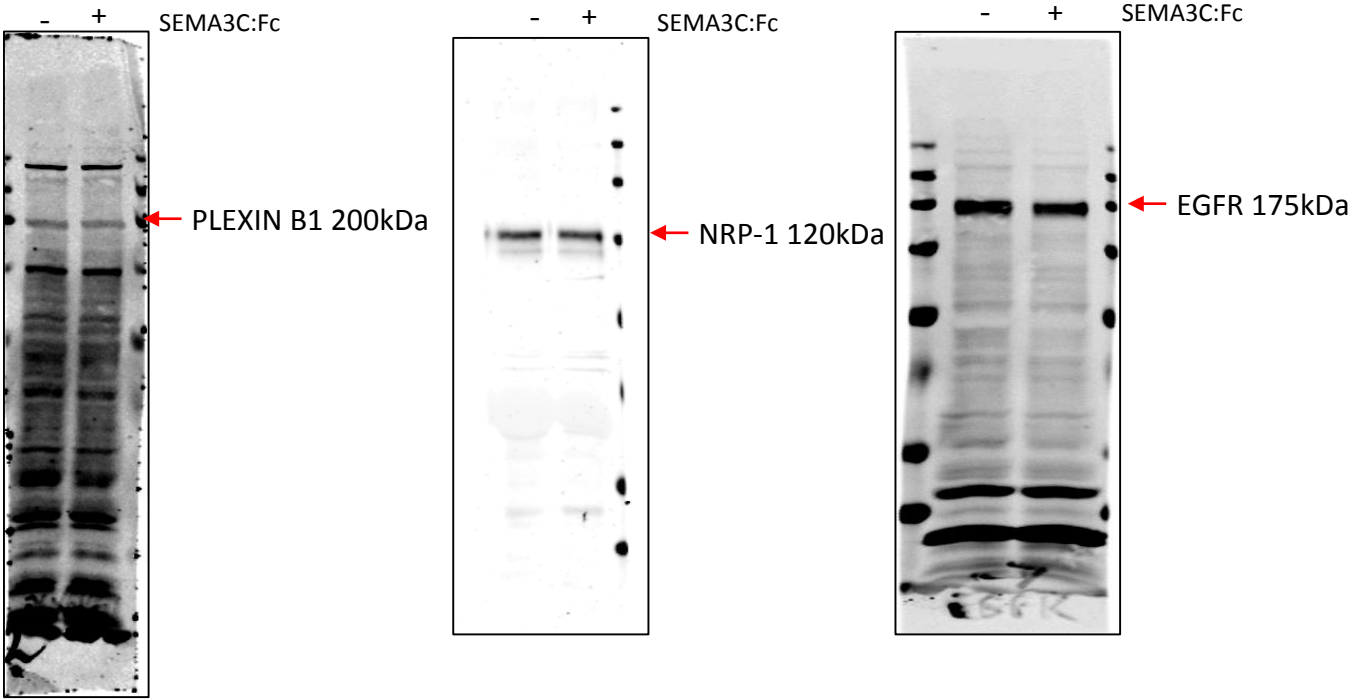

Figure 2 E

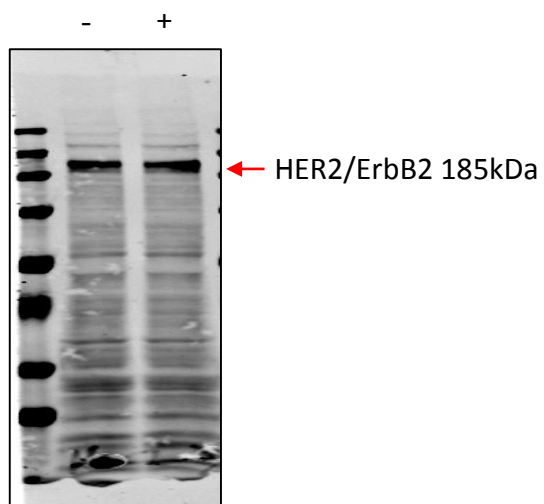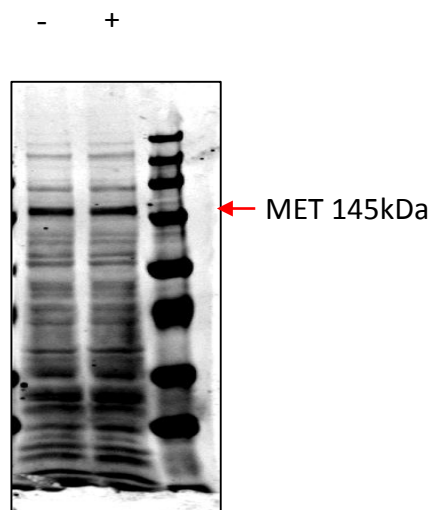

Supplement: Supplementary file 5 — Source Data for Figure 2 [file EMMM-10-219-s003.pdf]

Figure 3A

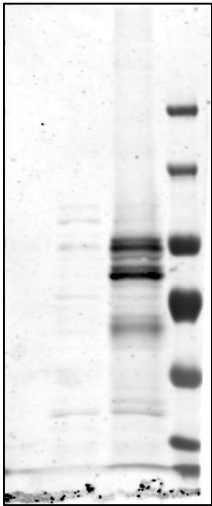

← Sema3C 83kDa

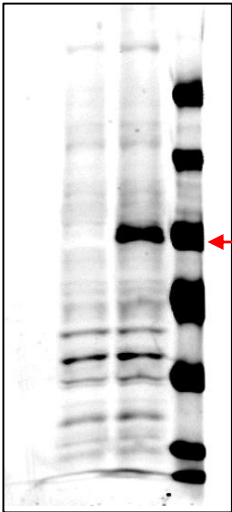

← HIS 83kDa

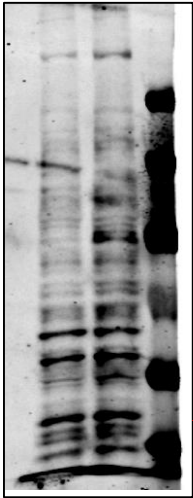

← ACTIN 42kDa

Supplement: Supplementary file 6 — Source Data for Figure 3 [file EMMM-10-219-s004.pdf]

Figure 7D

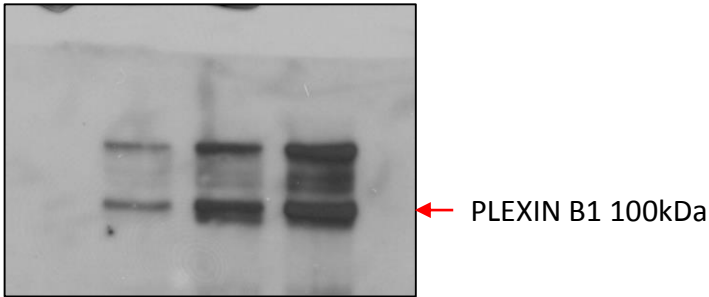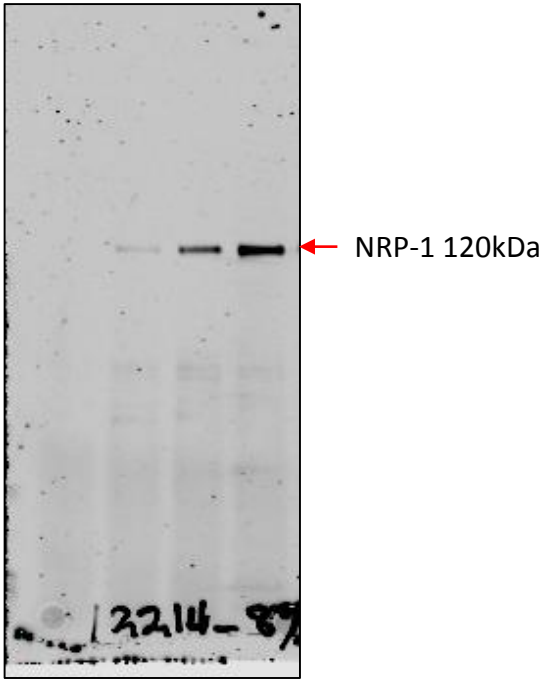

Supplement: Supplementary file 9 — Source Data for Figure 7 [file EMMM-10-219-s007.pdf]
