# Supplementary material for: SEMA3C drives cancer growth by transactivating multiple receptor tyrosine kinases via Plexin B1
Source: EMBO Mol Med. 2018 Jan 18;10(2):219–38. doi: 10.15252/emmm.201707689 (PMC5801490; doi:10.15252/emmm.201707689)

Figure 4A

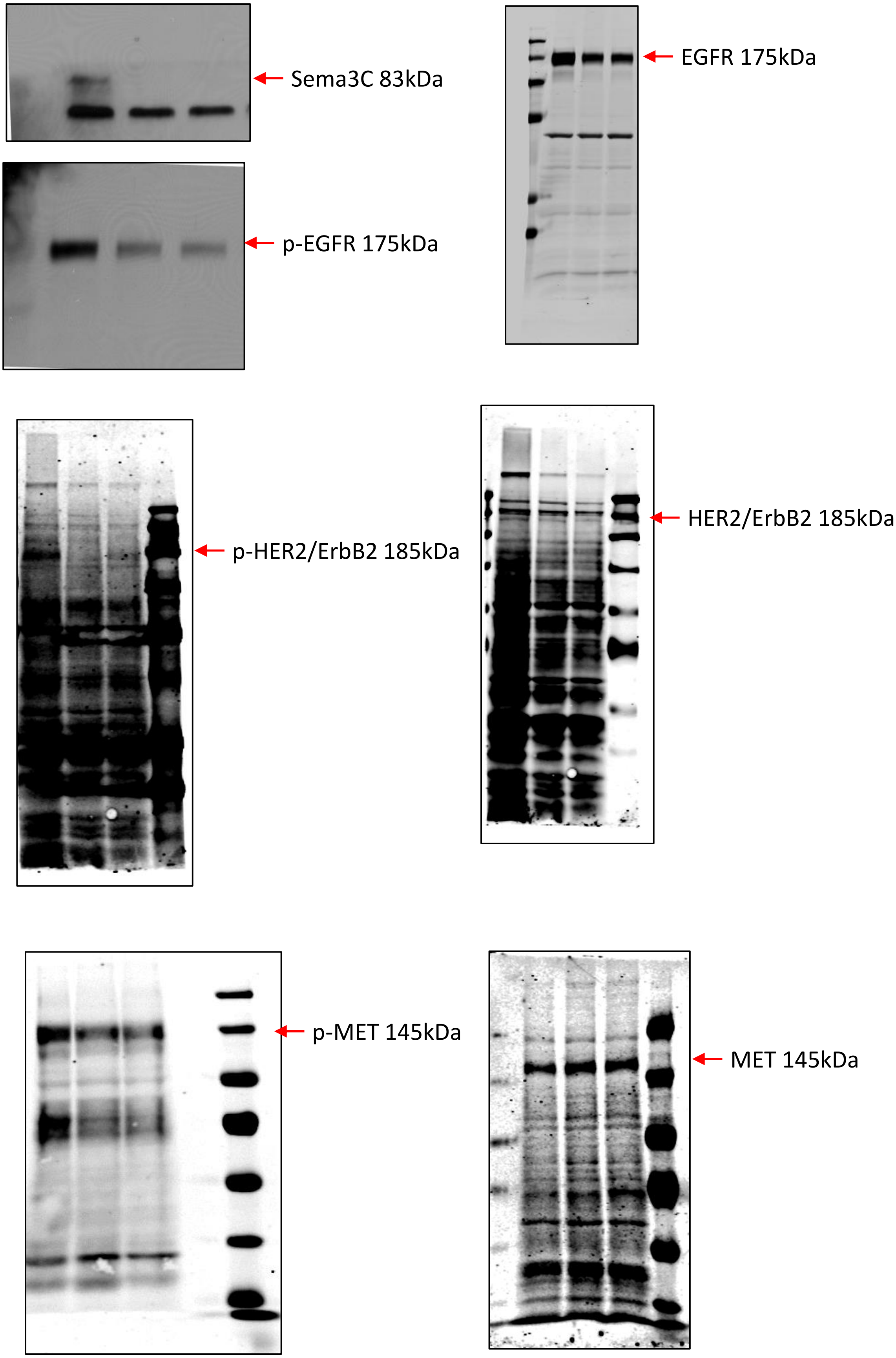
Sema3C 83kDa
EGFR 175kDa
p-EGFR 175kDa
p-HER2/ErbB2 185kDa
HER2/ErbB2 185kDa
p-MET 145kDa
MET 145kDa

Figure 4A

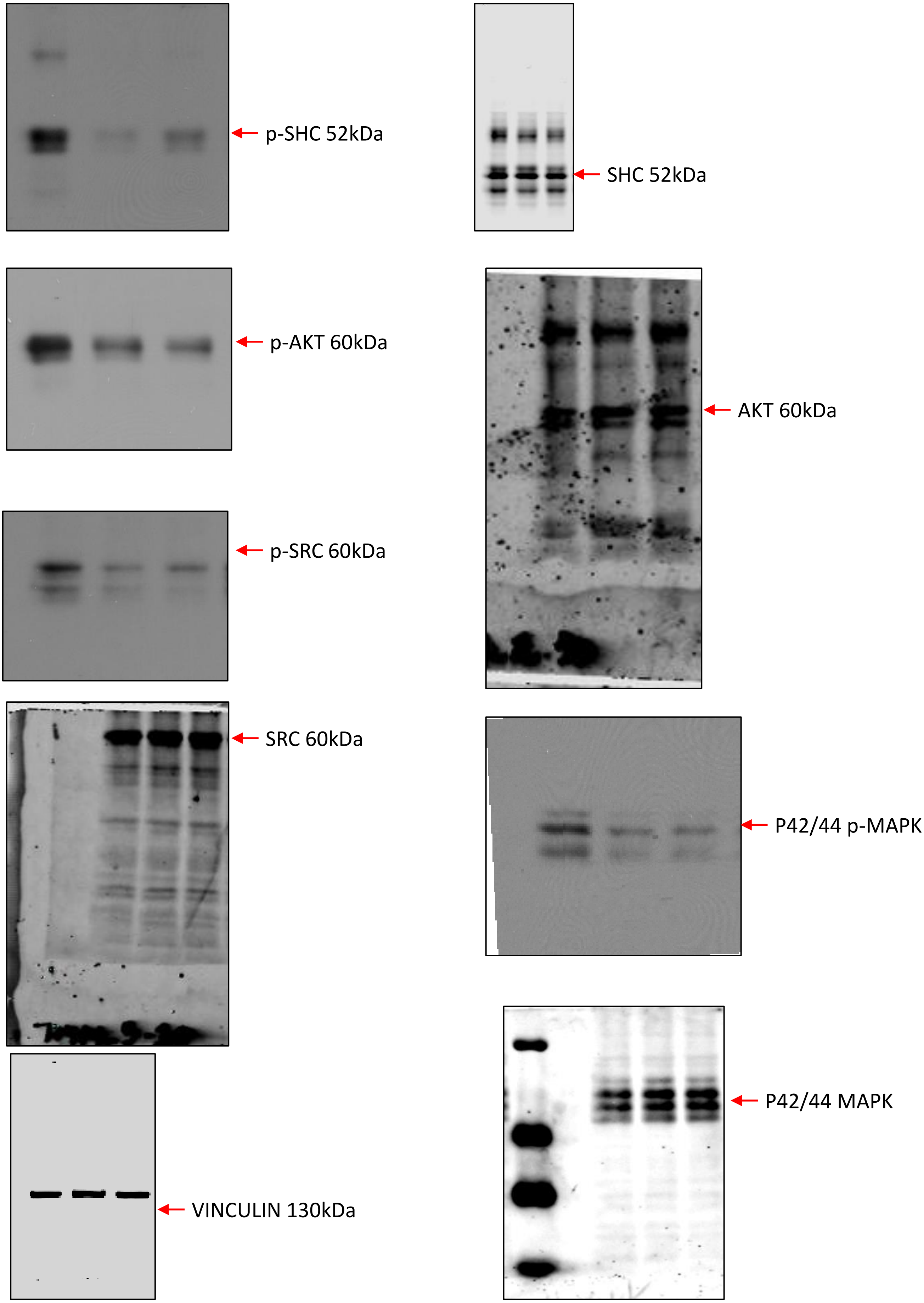

p-SHC 52kDa
SHC 52kDa
p-AKT 60kDa
AKT 60kDa
p-SRC 60kDa
SRC 60kDa
P42/44 p-MAPK
VINCULIN 130kDa
P42/44 MAPK

Figure 4B

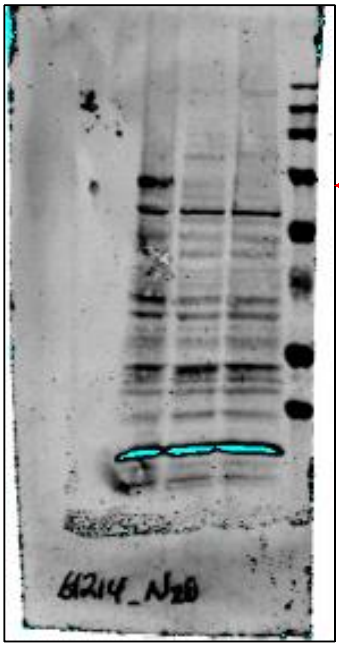

Sema3C 83kDa

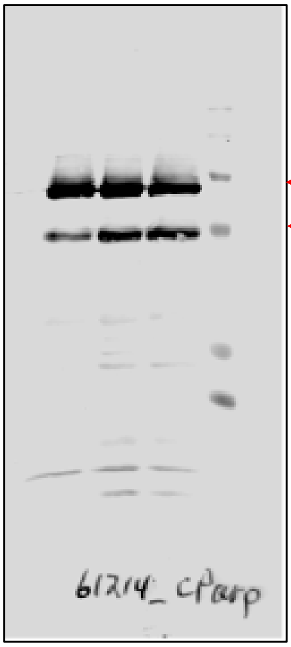

PARP 119kDa

c-PARP 89kDa

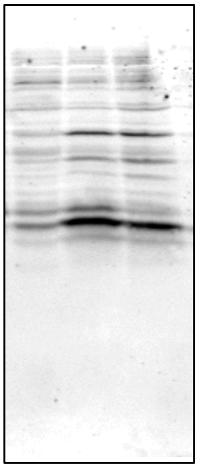

c-CASPASE-3 19 kDa

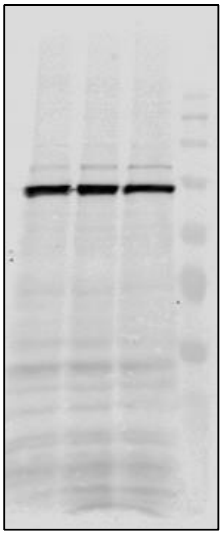

VINCULIN 130kDa

Figure 4C

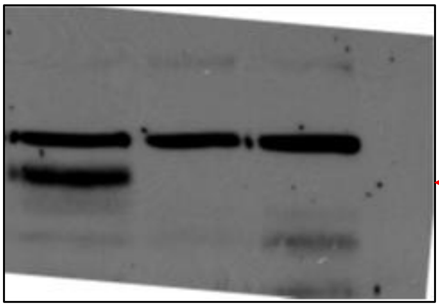

Sema3C 83kDa

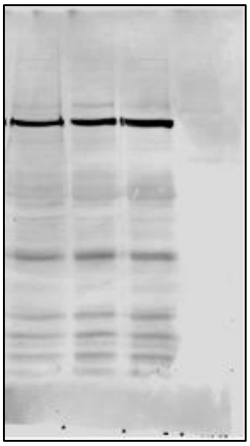

VINCULIN 130kDa

Figure 4D

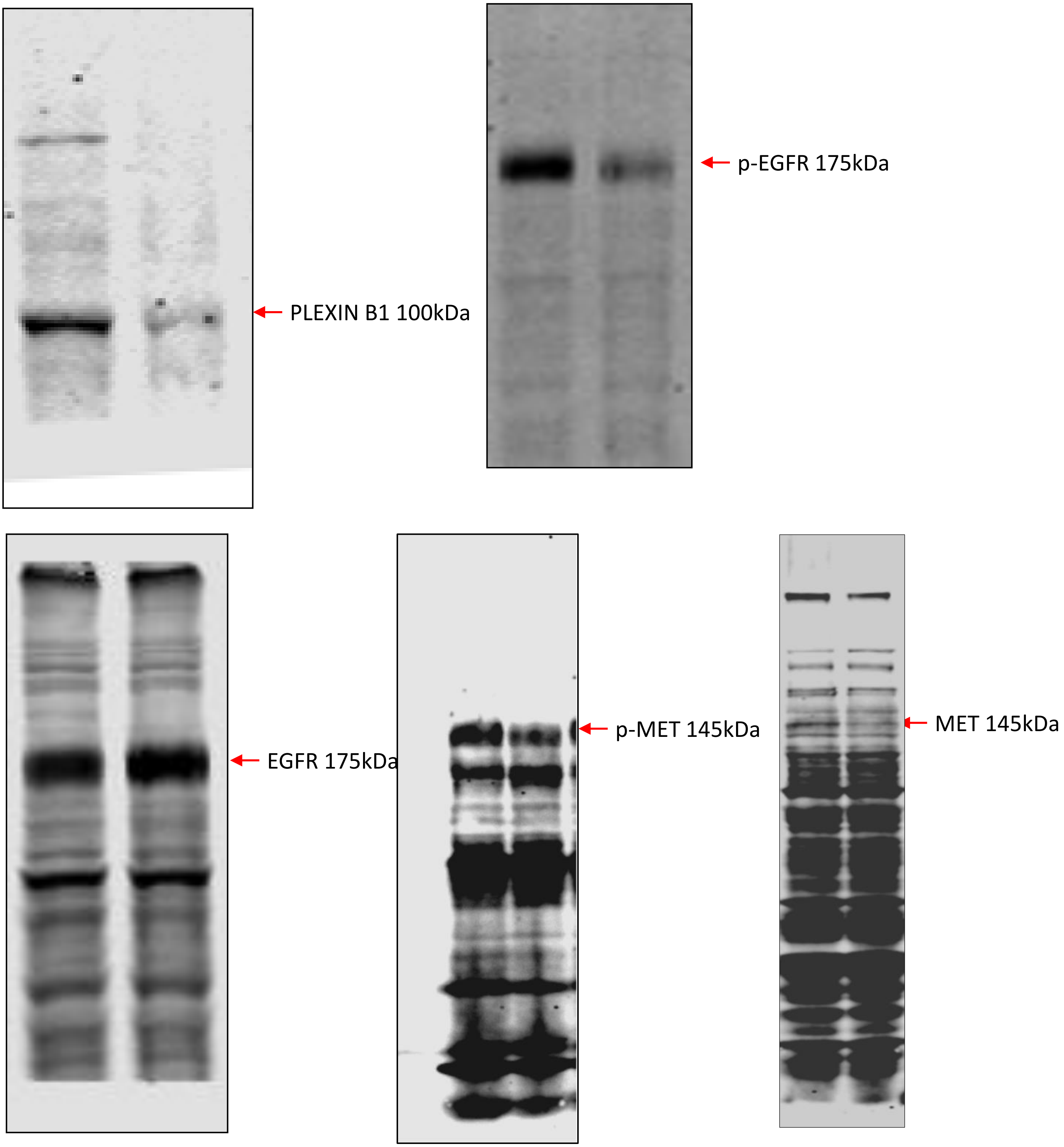
PLEXIN B1 100kDa
p-EGFR 175kDa
EGFR 175kDa
p-MET 145kDa
MET 145kDa

Figure 4D

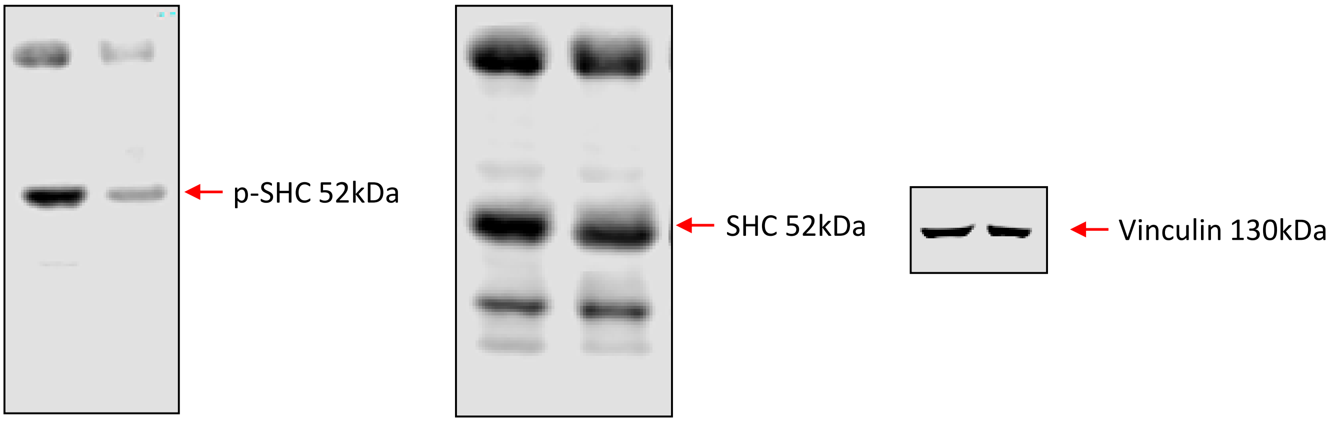

Figure 4H

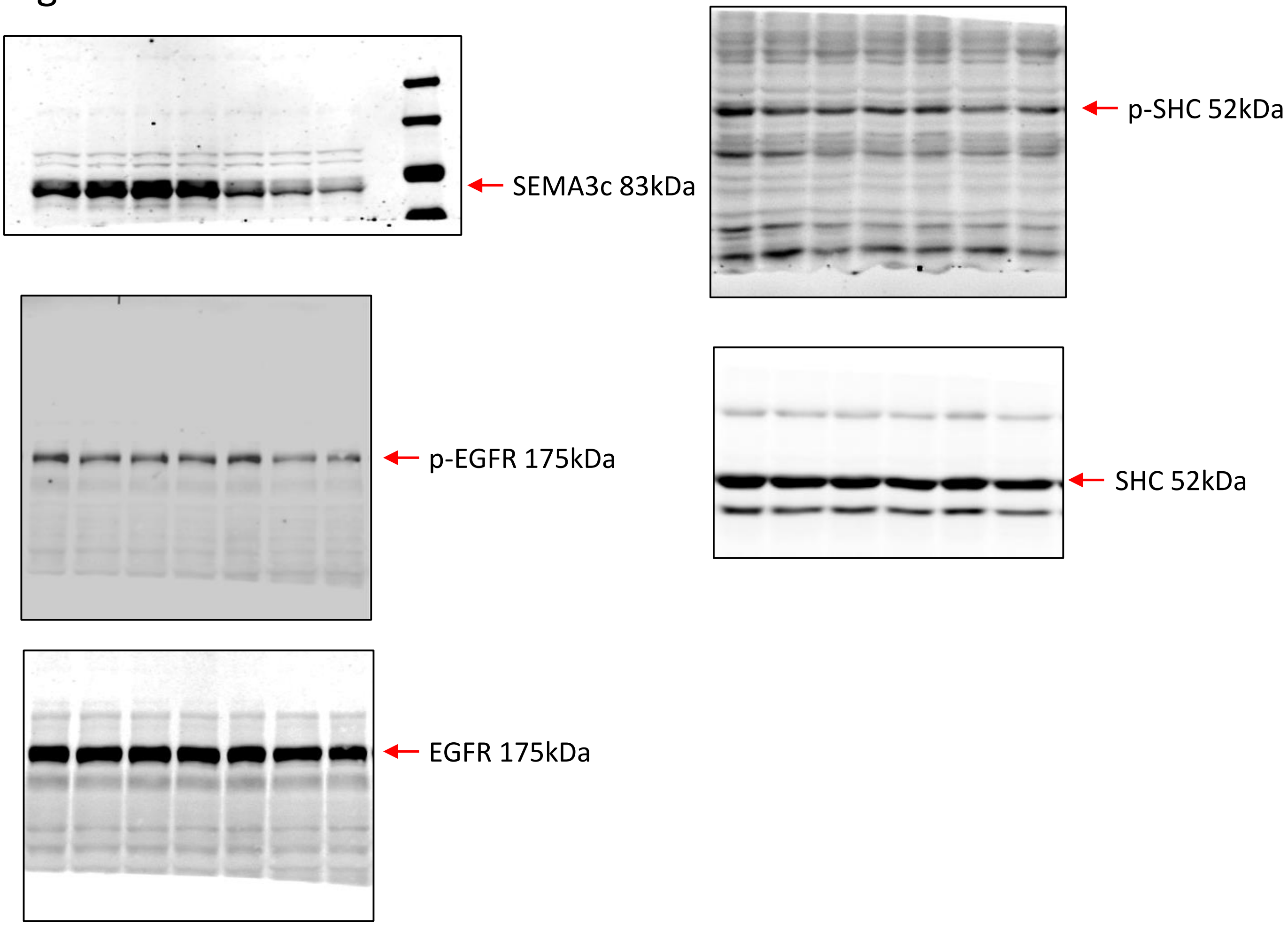

Figure 4H

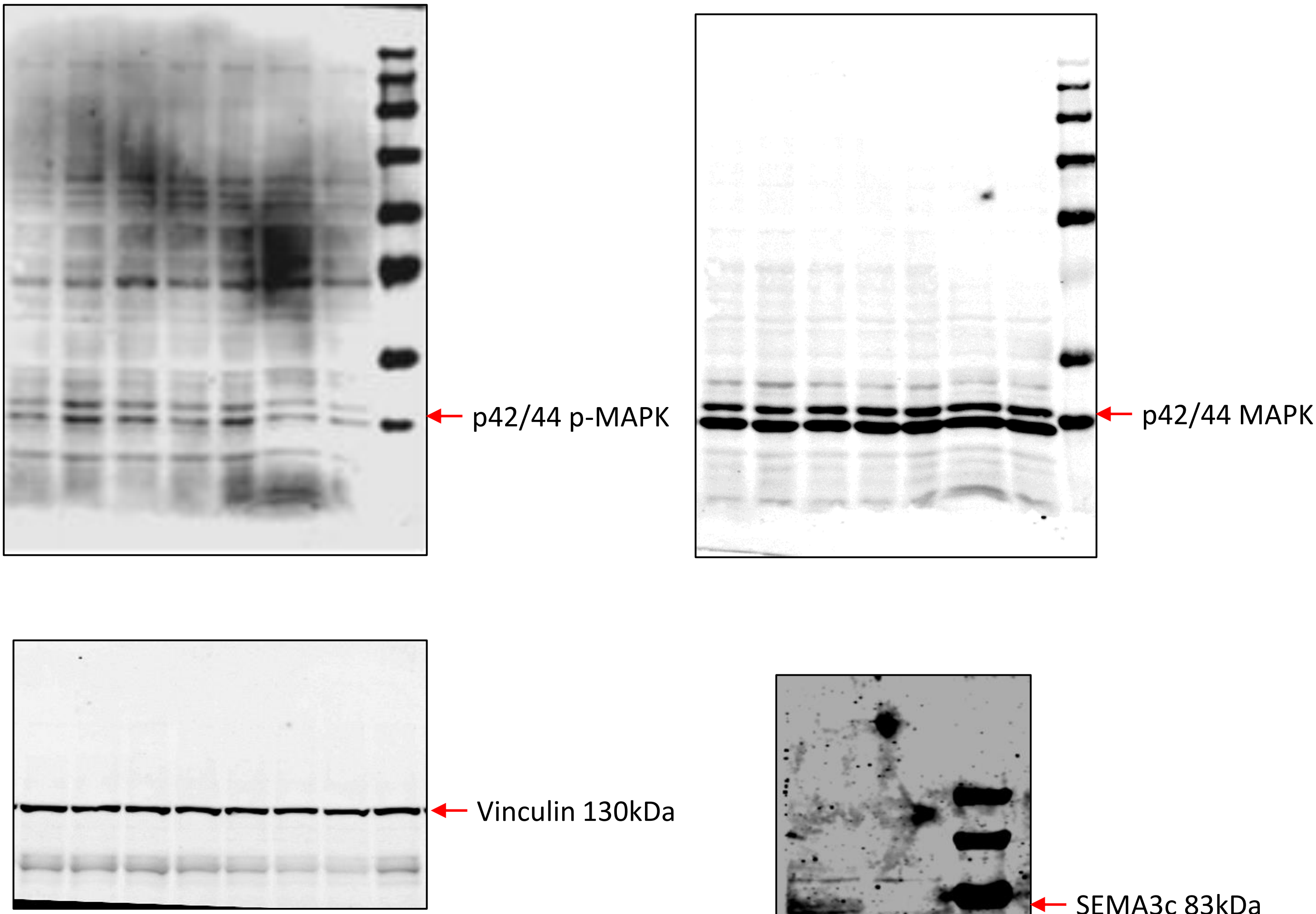

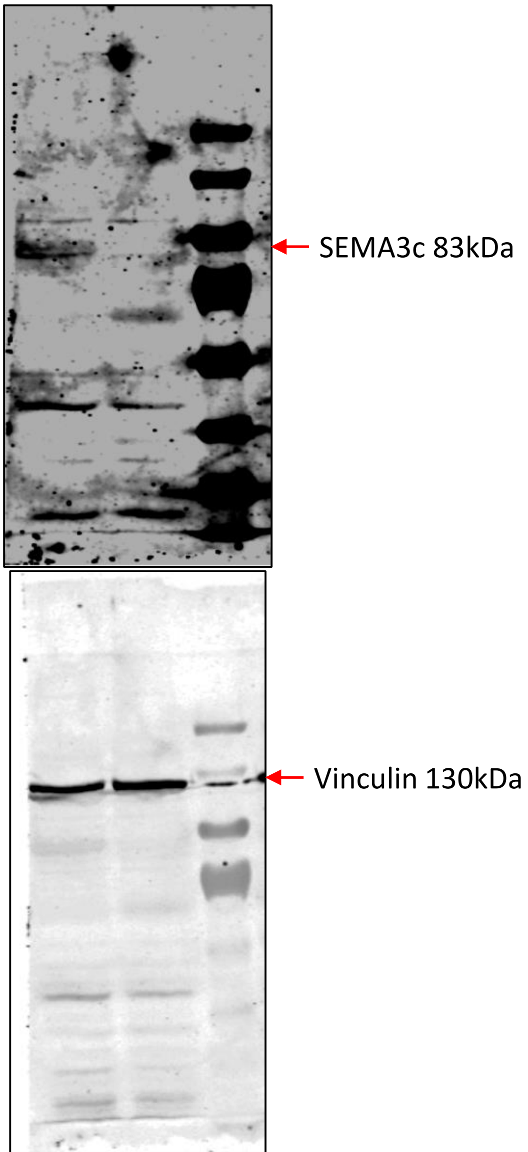

Figure 4I

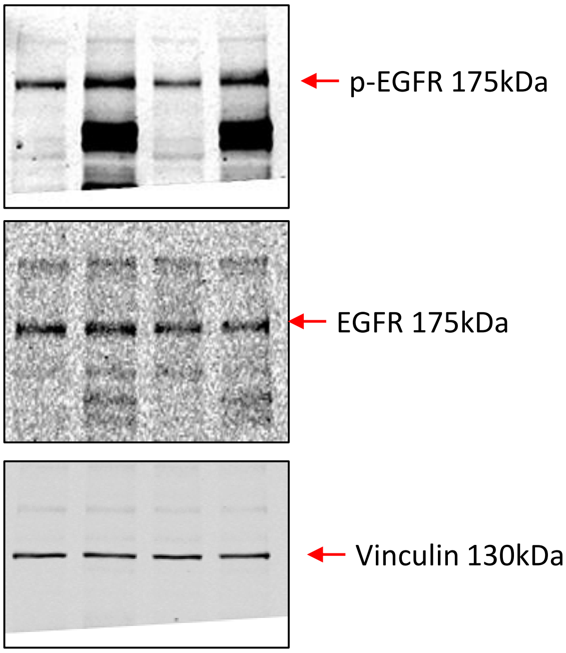

Supplement: Supplementary file 7 — Source Data for Figure 4 [file EMMM-10-219-s005.pdf]
