# Supplementary material for: SEMA3C drives cancer growth by transactivating multiple receptor tyrosine kinases via Plexin B1
Source: EMBO Mol Med. 2018 Jan 18;10(2):219–38. doi: 10.15252/emmm.201707689 (PMC5801490; doi:10.15252/emmm.201707689)

Experiment repeated independently to provide source data

Figure 6B

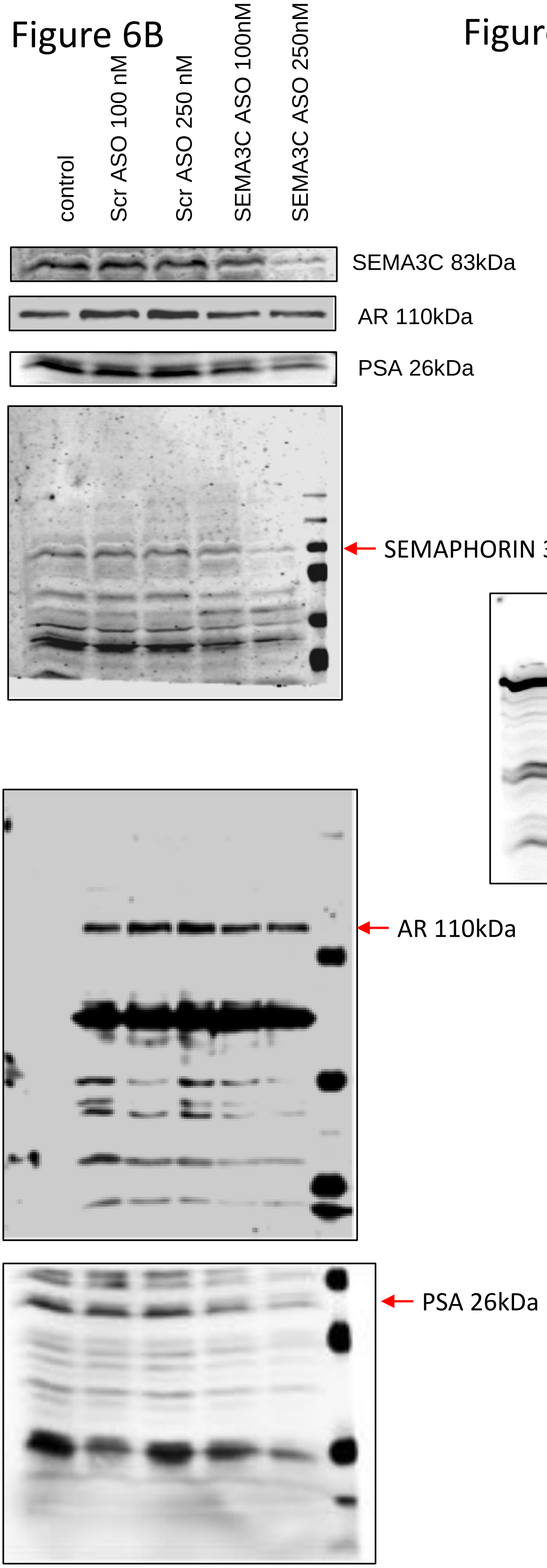

Figure 6C

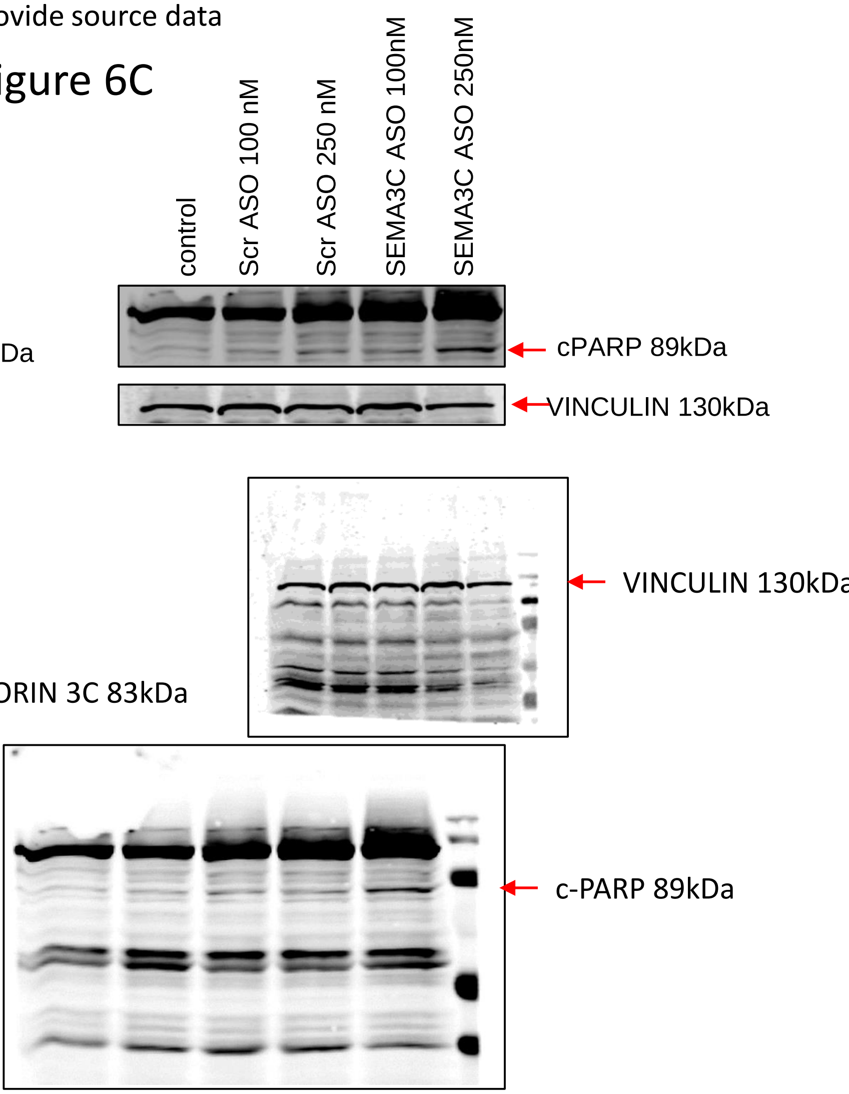

Supplement: Supplementary file 8 — Source Data for Figure 6 [file EMMM-10-219-s006.pdf]
