# Supplementary material for: SEMA3C drives cancer growth by transactivating multiple receptor tyrosine kinases via Plexin B1
Source: EMBO Mol Med. 2018 Jan 18;10(2):219–38. doi: 10.15252/emmm.201707689 (PMC5801490; doi:10.15252/emmm.201707689)

Figure 8D

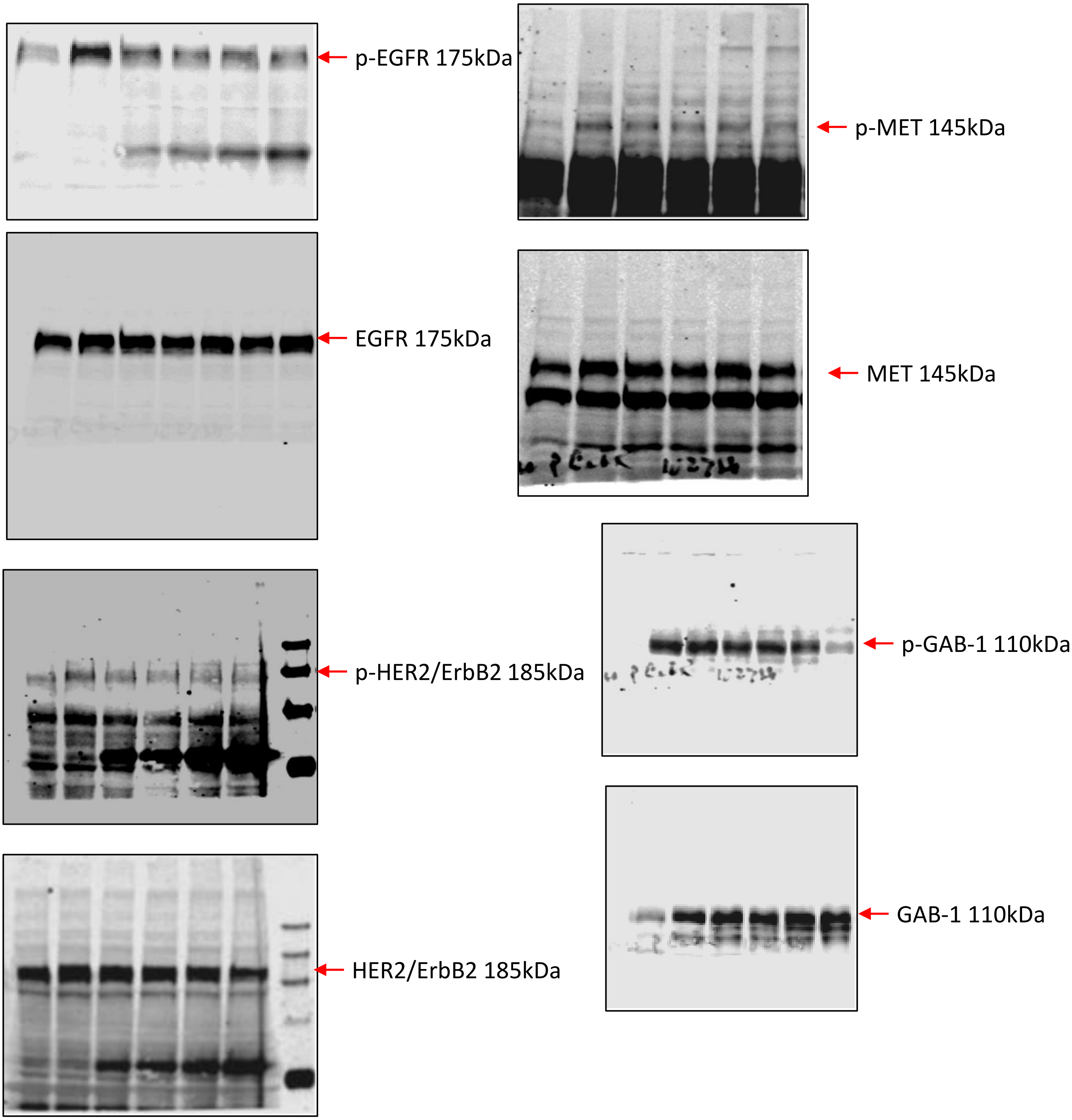
p-EGFR 175kDa
p-MET 145kDa
EGFR 175kDa
MET 145kDa
p-GAB-1 110kDa
p-HER2/ErbB2 185kDa
GAB-1 110kDa
HER2/ErbB2 185kDa

Figure 8D

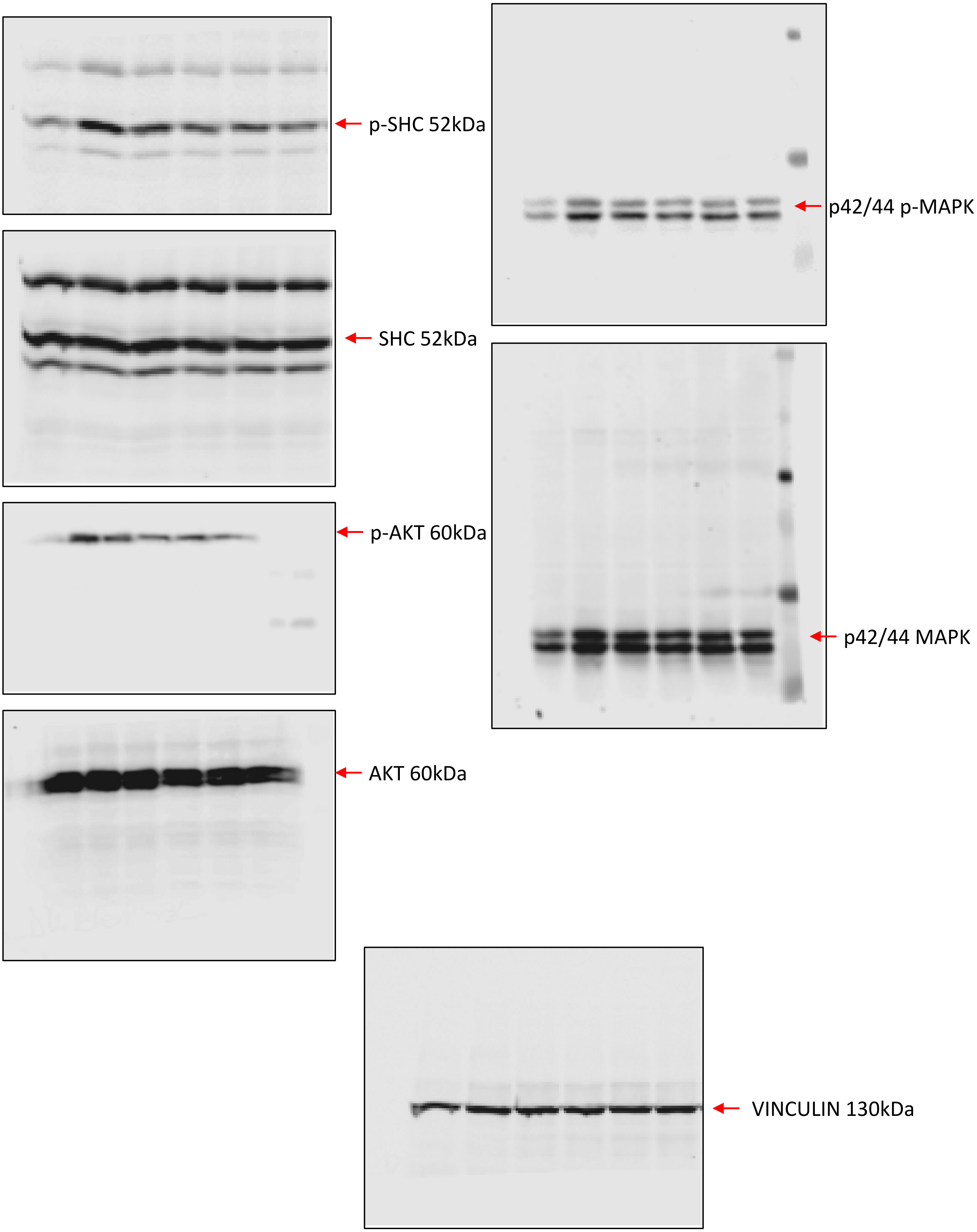
p-SHC 52kDa
SHC 52kDa
p-AKT 60kDa
AKT 60kDa
p42/44 p-MAPK
p42/44 MAPK
VINCULIN 130kDa

Figure 8E

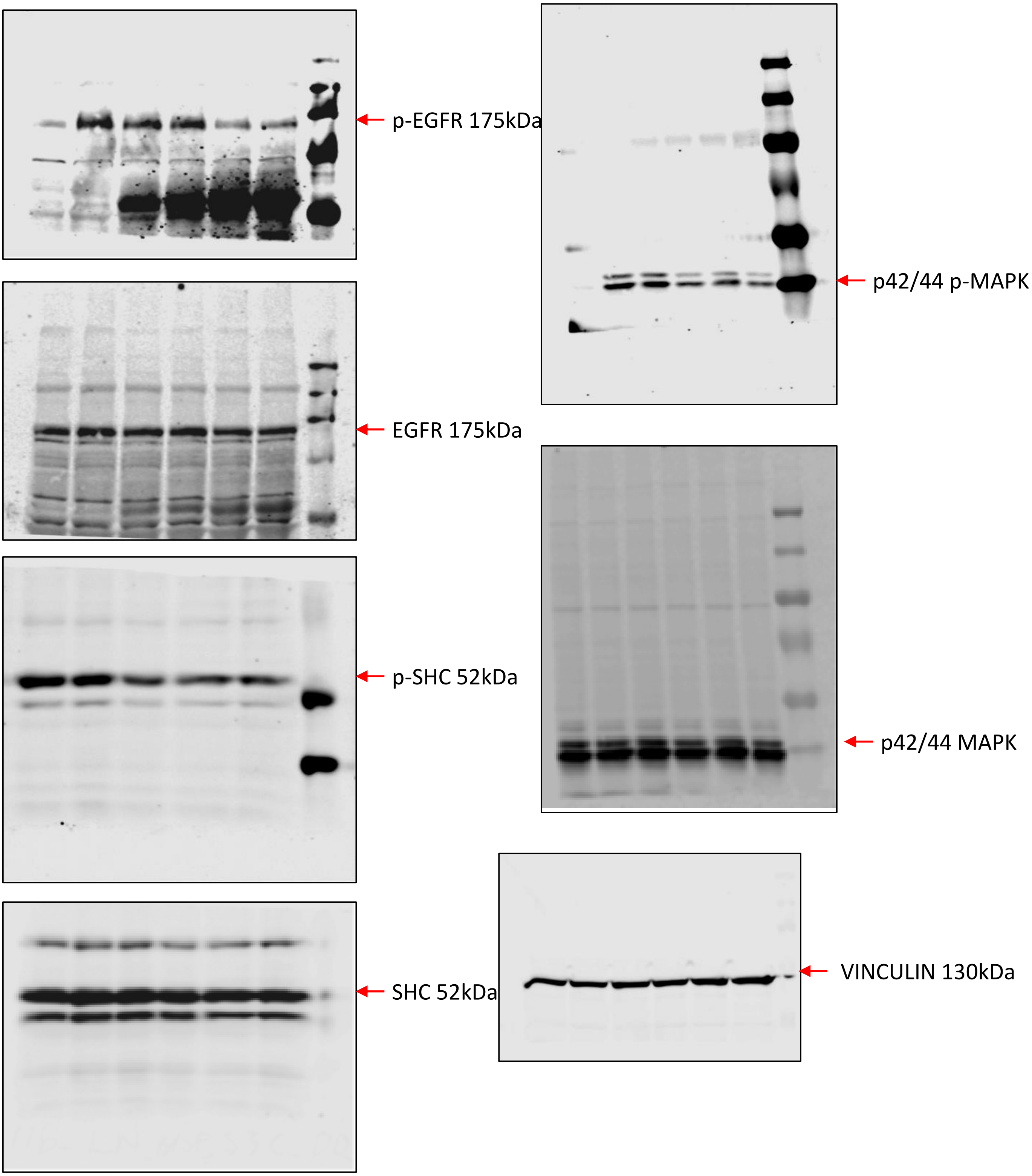

p-EGFR 175kDa
EGFR 175kDa
p-SHC 52kDa
SHC 52kDa
p42/44 p-MAPK
p42/44 MAPK
VINCULIN 130kDa

Figure 8F

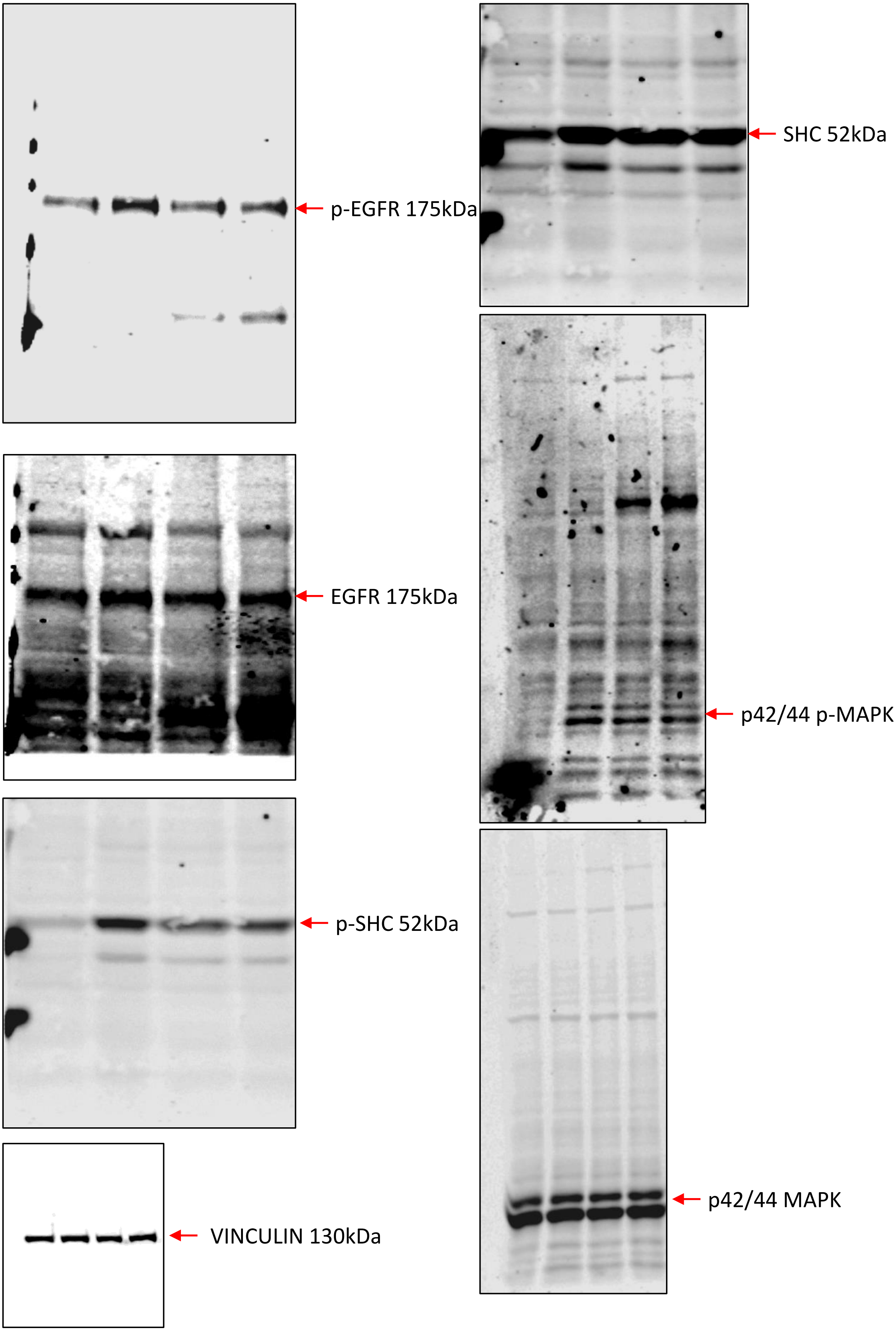
p-EGFR 175kDa
EGFR 175kDa
p-SHC 52kDa
VINCULIN 130kDa
SHC 52kDa
p42/44 p-MAPK
p42/44 MAPK

Figure 8G

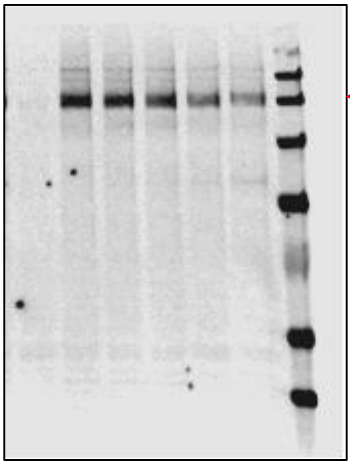

p-EGFR 175kDa

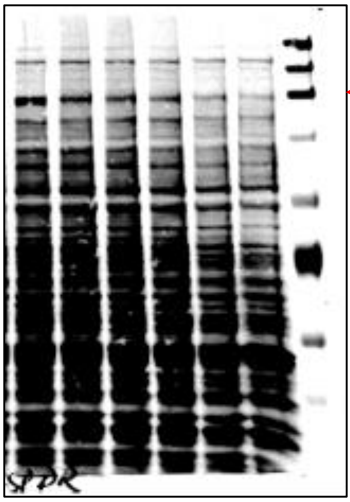

EGFR 175kDa

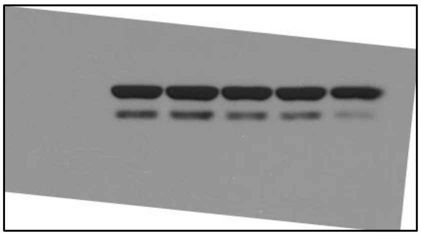

p-SHC 46kDa

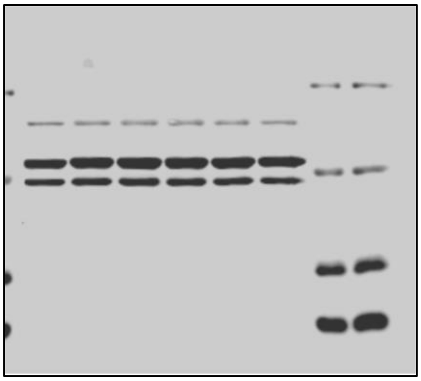

SHC 46kDa

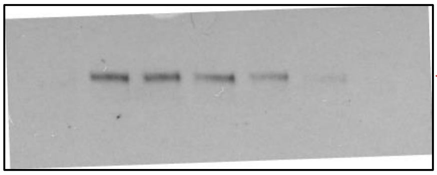

p-HER2/ErbB2 185kDa

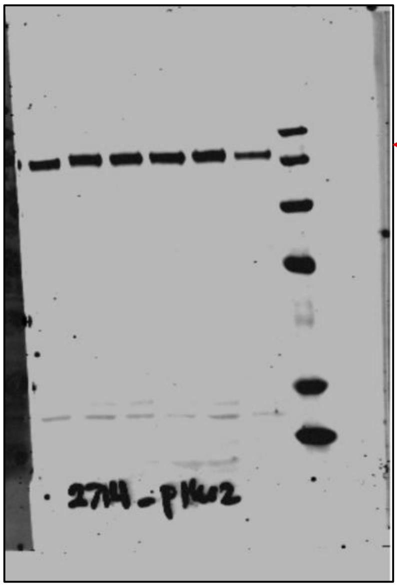

HER2/ErbB2 185kDa

Figure 8G

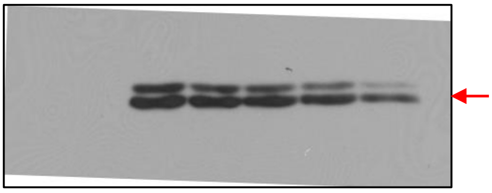

p42/44 p-MAPK

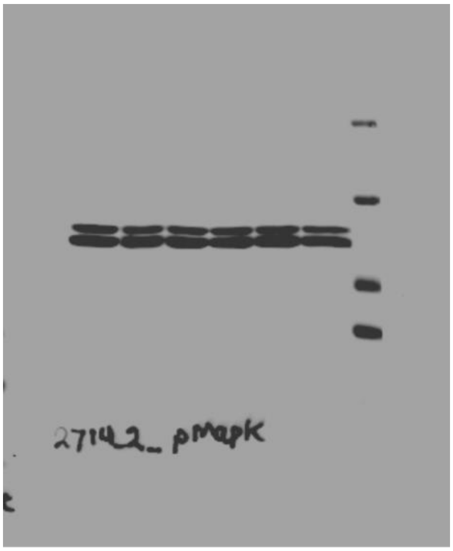

p42/44 MAPK

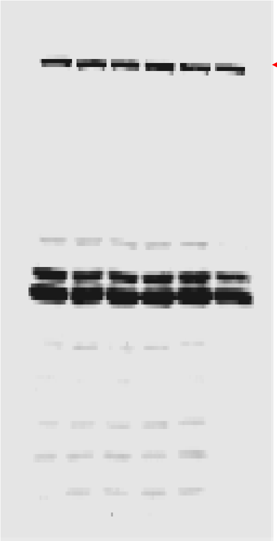

VINCULIN 130kDa

Figure 8H

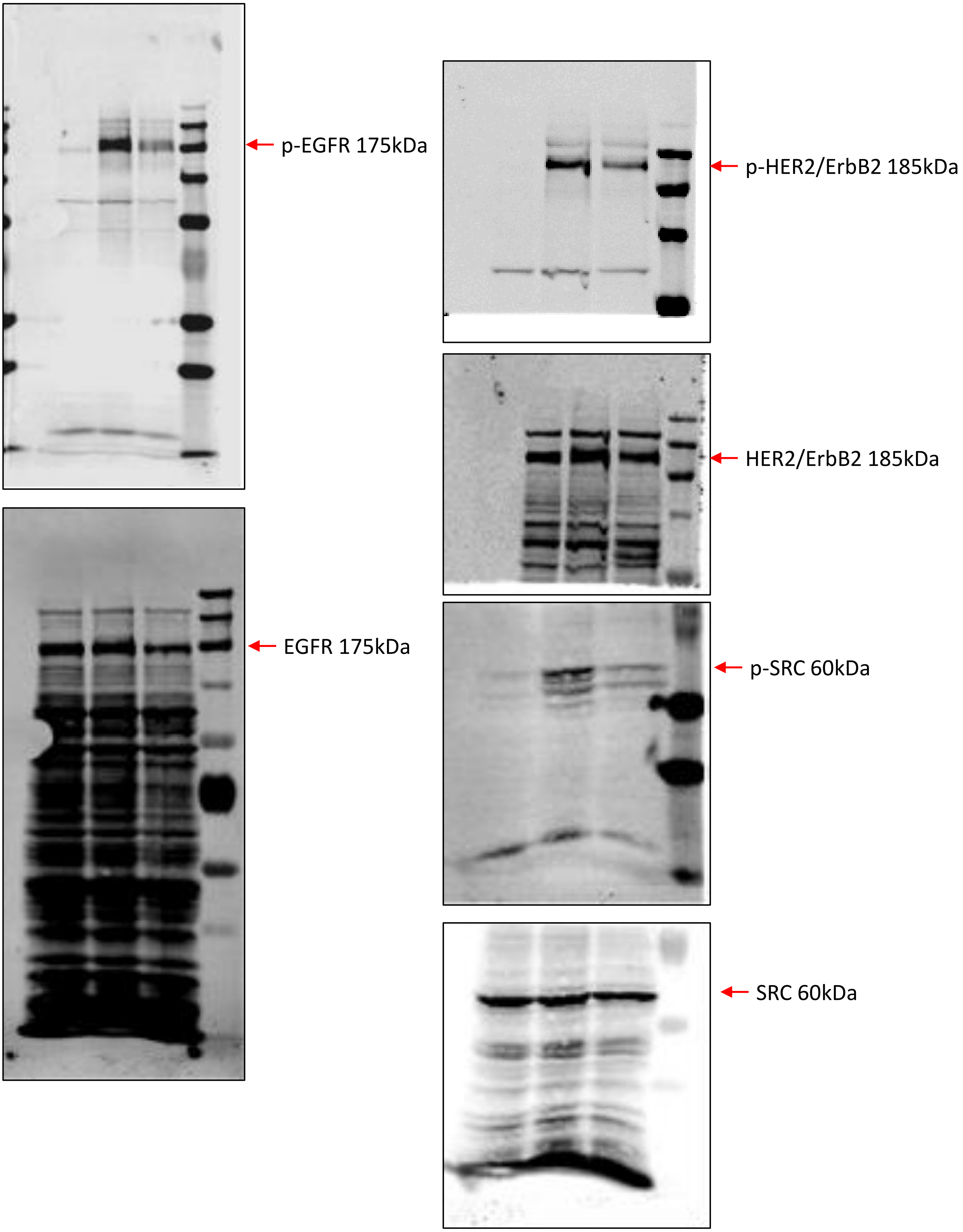

p-EGFR 175kDa
p-HER2/ErbB2 185kDa
HER2/ErbB2 185kDa
EGFR 175kDa
p-SRC 60kDa
SRC 60kDa

Figure 8H

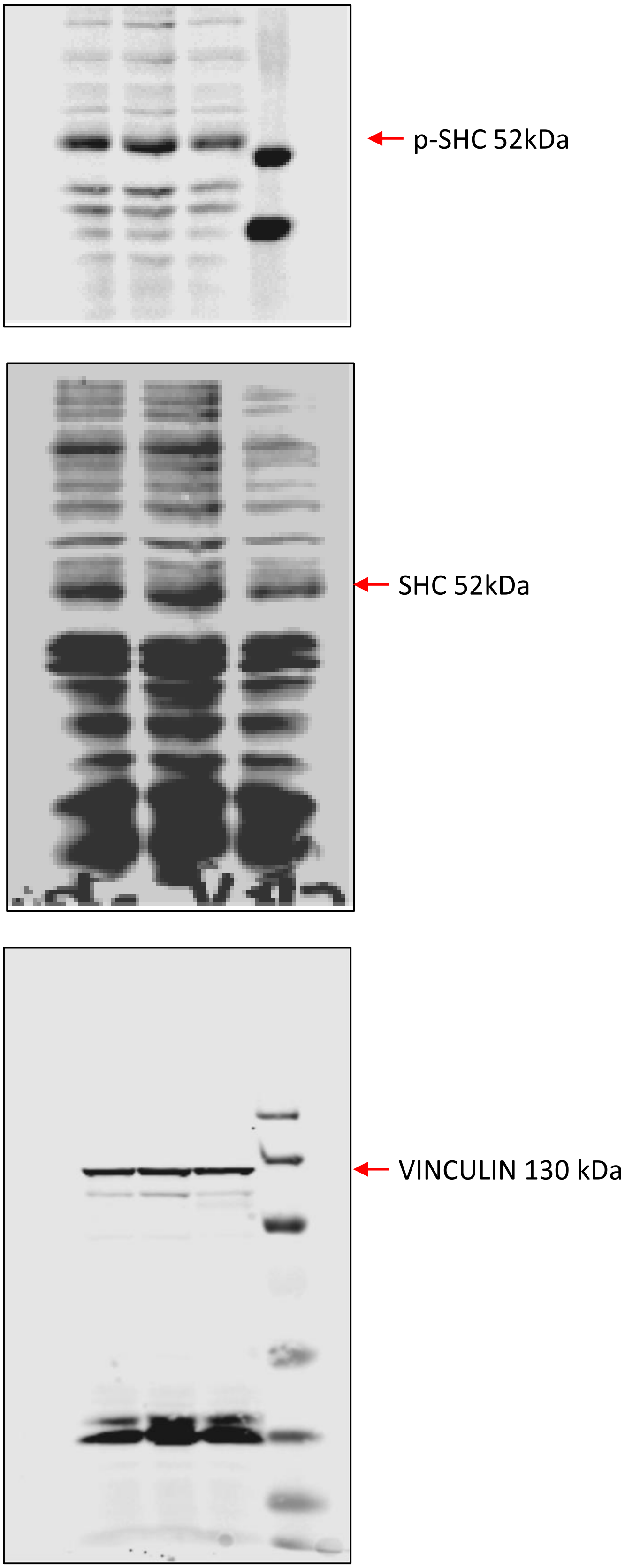

Supplement: Supplementary file 10 — Source Data for Figure 8 [file EMMM-10-219-s008.pdf]
